# A mass spectrometry guided approach for the identification of novel vaccine candidates in gram-negative pathogens

**DOI:** 10.1038/s41598-019-53493-8

**Published:** 2019-11-22

**Authors:** Daniel Hornburg, Tobias Kruse, Florian Anderl, Christina Daschkin, Raphaela P. Semper, Kathrin Klar, Anna Guenther, Raquel Mejías-Luque, Nicole Schneiderhan-Marra, Matthias Mann, Felix Meissner, Markus Gerhard

**Affiliations:** 1 Institut für Medizinische Mikrobiologie, Immunologie und Hygiene, Technische Universität München, Munich, Germany; 2 Max-Planck-Institute for Biochemistry, Martinsried, Germany; 3 ImevaX GmbH, Munich, Germany; 4 NMI Natural and Medical Sciences Institute, University of Tübingen, Reutlingen, Germany; 5 Present Address: Stanford University, School of Medicine, San Francisco, USA; 6 German Center for infection research, partner site Munich, Munich, Germany

**Keywords:** Protein vaccines, Protein vaccines

## Abstract

Vaccination is the most effective method to prevent infectious diseases. However, approaches to identify novel vaccine candidates are commonly laborious and protracted. While surface proteins are suitable vaccine candidates and can elicit antibacterial antibody responses, systematic approaches to define surfomes from gram-negatives have rarely been successful. Here we developed a combined discovery-driven mass spectrometry and computational strategy to identify bacterial vaccine candidates and validate their immunogenicity using a highly prevalent gram-negative pathogen, *Helicobacter pylori*, as a model organism. We efficiently isolated surface antigens by enzymatic cleavage, with a design of experiment based strategy to experimentally dissect cell surface-exposed from cytosolic proteins. From a total of 1,153 quantified bacterial proteins, we thereby identified 72 surface exposed antigens and further prioritized candidates by computational homology inference within and across species. We next tested candidate-specific immune responses. All candidates were recognized in sera from infected patients, and readily induced antibody responses after vaccination of mice. The candidate jhp_0775 induced specific B and T cell responses and significantly reduced colonization levels in mouse therapeutic vaccination studies. In infected humans, we further show that jhp_0775 is immunogenic and activates IFNγ secretion from peripheral CD4^+^ and CD8^+^ T cells. Our strategy provides a generic preclinical screening, selection and validation process for novel vaccine candidates against gram-negative bacteria, which could be employed to other gram-negative pathogens.

## Introduction

Vaccination is recognized as the most effective way to prevent infectious diseases. Despite the eradication or control of many diseases, vaccines against major human pathogens – especially gram-negatives-are still missing, as are streamlined approaches for their development. Among the key challenges in vaccine design is the selection of antigens that are capable of inducing protective immunity against the pathogen. For this purpose, antigens must be accessible for the immune system and be able to elicit specific humoral and cellular immune responses, without triggering an immune response against self-antigens or non-pathogenic microorganisms. Protein antigens are widely used for vaccination due to their sequence-encoded specific recognition by the immune system, however, from the thousands of expressed proteins e.g. in a bacterial pathogen, only few fulfill these criteria. Therefore, generic and rational selection processes for the identification of protein vaccine candidates are highly valuable.

To identify suitable vaccine candidates, screening approaches have to interrogate a pathogen’s proteome according to biochemical/biophysical and functional characteristics. Physical accessibility of the antigens is key for the host’s immune system to recognize immunogenic epitopes. Outer membrane proteins (OMPs) are therefore considered most promising targets to select vaccine candidates, especially due to their physical accessibility on the bacterial surface, and ability to be bound by opsonizing antibodies^1–4^. In a pioneering in silico approach, Pizza *et al*.^5^ predicted several OMPs with potentiality to confer protection against serogroup B *N. meningitides*, some of these antigens were finally included in the Novartis 4CMenB vaccine^6^.

State of the art approaches identify OMPs by time consuming membrane extraction or labeling strategies and subsequent mass spectrometry (MS). While these strategies delivered novel vaccine candidates, they are inherently limited in their capacity to differentiate between inner or outer membrane localization and probe actual surface accessibility. To map surface exposed proteins on bacteria, the “surface shaving” approach was introduced for gram-positive bacteria (Rodríguez-Ortega *et al*. 2006) and also evaluated for gram-negative bacteria, unicellular fungi, as well as pluricellular organisms^7,8^. Here, a protease is added to a live bacterial culture to release peptides of surface accessible proteins, which can be identified via MS. However, since modern MS can detect even trace amounts of proteins, the presence of few lysed bacterial cells hampers the efficient and exclusive identification of surface-exposed proteins, which poses a challenge in particular for gram-negative bacteria^9^. To overcome current limitations, we developed a streamlined MS-based surface shaving approach that simultaneously optimizes multiple experimental variables to identify cell surface exposed proteins and discriminate them from proteins derived from cell death. We further computationally prioritized proteins by high homology within the pathogenic bacterial genus and low homology to other bacterial species and the host.

In this study, we focused on the gram-negative human pathogen *H. pylori* for which to date no effective vaccine is available. *H. pylori* infects half of the world’s population and is the leading cause of gastric cancer^10^. Therapeutic options are increasingly limited by dramatically rising antibiotic resistances^11^, raising the demand for vaccines to combat this infection. In our study, we experimentally tested the immunogenicity of antigens identified through our pipeline in humans as well as efficacy in murine vaccination studies. As infections with gram-negative bacteria pose a major threat for human health, and eight out of the top ten WHO defined priority pathogens are gram-negatives for which no vaccines are available^12^, our approach establishes a paradigm blueprint for the streamlined identification of candidates against those pathogens.

## Results

### Establishment of a surfome-shaving based pipeline for vaccinology in gram-negative pathogens

**Figure 1:**
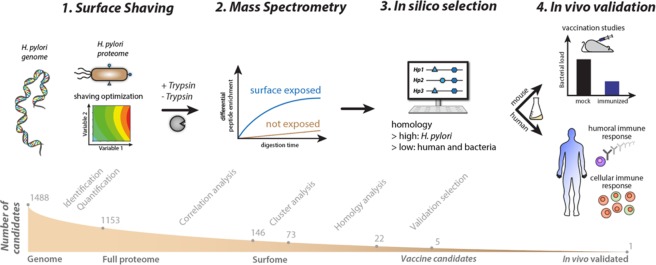
Workflow for discovery and validation of novel *H. pylori* vaccine candidates. (**1**) Design of experiment optimized shaving conditions are used to isolate surface exposed proteins. A live culture of *H. pylori* is treated with trypsin and the supernatant analyzed by (**2**) Quantitative mass spectrometry. After recombinant production, surface exposure of proteins is evaluated based on their enrichment profile. (**3**) Candidates are further selected based on their conservation and selectivity. (**4**) *In vivo* validation: candidates are evaluated for their efficacy in mice and for their potential to elicit B and T cell responses in humans.

**Figure 2:**
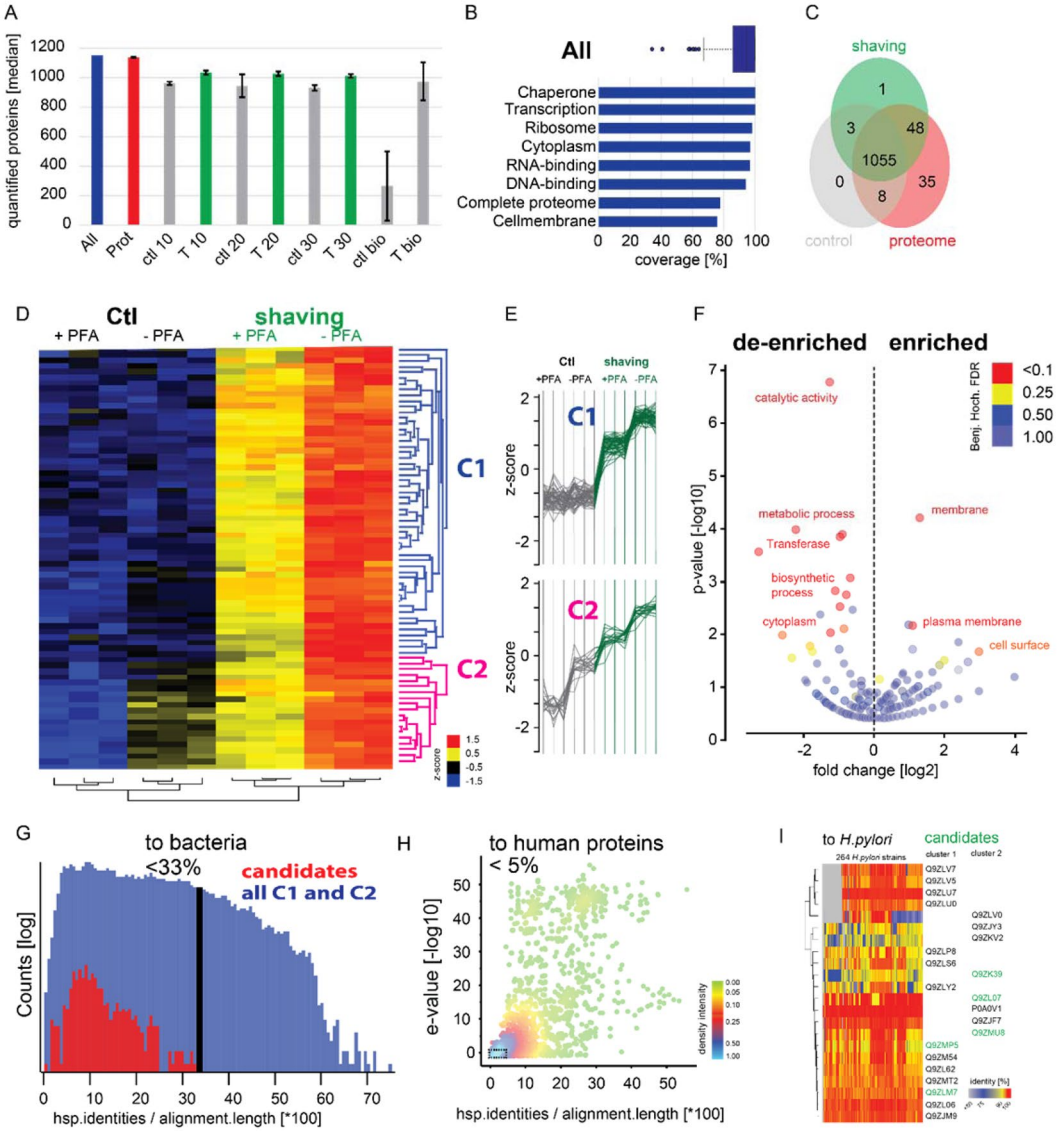
Identification of surface exposed proteins and selection of candidates. (**A**) Bar Plot depicting number of quantified proteins (median number of quantification events) for the respective conditions. Error bars denote standard deviation (Prot: whole proteome quantified, ctl: control, T: trypsin treatment for, 10, 20, or 30 min:, bio: biotinylation). Controls comprise samples without the addition of trypsin (ctl 10, 20, 30) and without biotinylation (ctl bio). (**B**) Percentage of coverage for individual functional groups (annotations, see Supplementary Table) with respect to all quantified proteins in this study (including all conditions). The box plot labeled ‘All’ depicts the percentage coverage of all annotations with a group size (number of annotated proteins in the entire *in silico* proteome) >4. (**C**) Venn diagram depicting the overlap of proteins quantified in at least 3 replicates (‘intensity’) for the samples. Proteins were filtered for contaminants, only identified by side and reverse. “Control” contains all time course control samples, shaving contains all time course shaving samples, proteome contains all complete proteome samples. (**D**) Unsupervised hierarchical clustering and heat map of significantly correlating proteins (median of replicates, z-scored). Clusters c1 (blue) and c2 (pink) show the expected pattern for surface association and/ or secretion of proteins and were selected as putative surface-associated vaccine candidates for subsequent analysis and prioritization (**E**) Extracted profile of proteins in cluster c1 and cluster c2. Samples with trypsin shaving indicated in green. (**F**) Volcano plot depicting annotations de-enriched and enriched in cluster 1 and cluster 2 compared to all proteins detected in the surfome over time (including control samples). Enrichment was calculated with a fisher exact test (see Supplementary Table cluster enrichment). Proteins annotated with membranes are enriched with a p-value <0.0001. (**G**) Histogram (log count) depicting the identity of the 72 surfome candidates compared to bacterial proteins (excluding *H. pylori*). Candidates were selected to have an identity lower than 33%. (**H**) Scatter plot showing the identity of 72 surfome proteins compared to human proteins. Candidates were selected to have an identity lower than 5% and an e-value [−log10] of less than 1. (**I**) Heat map of remaining candidate proteins indicating their identity compared to all 264 *H. pylori* strains. Protein vaccine candidates that were further characterized for their surface exposure through staining experiments (Fig. 3) are marked in green. The “cluster 1/2” annotation indicates associated protein cluster (**D**,**E**).

We established a combined experimental and computational vaccinology pipeline for gram-negative bacteria - using *H. pylori* as a model organism - that enables the identification of few promising vaccine candidates from thousands of expressed bacterial proteins. Our strategy combines statistical parameter optimization (*design-of-experiment* (DoE)^13^) guided surface shaving with quantitative MS followed by computational and functional validation (Fig. 1). First, we analyzed the abundance of the annotated *H. pylori* proteomes comprehensively and account close to 1,200 expressed proteins, which is, to our knowledge, the most comprehensive protein inventory of this pathogen to date (Fig. 2A, Supplementary Fig. 1). Our proteomics analysis covers functional protein classes well (on average 92%) and 80% of all putative membrane associated proteins (Fig. 2B).

Next, we tested experimental approaches to biochemically capture bacterial cell surface exposed proteins. We evaluated biotinylation of surface proteins and streptavidin purification^14^, which is the most frequently applied technology for surfome identification^15–18^, and identified 565 proteins (Supplementary Fig. 2). Since our MS pipeline detects minute amounts of proteins we cannot exclude that among these 565 proteins we identified a large proportion of cytoplasmic proteins e.g. due to cell lysis or diffusion of the linker into cells. To evaluate the efficiency of identifying surface proteins by biotinylation we performed an enrichment analysis. We did not observe a significant enrichment for membrane associated proteins (Supplementary Table Biotinylation), indicating that a proportion of the detected signals derives from labeling of cytoplasmic proteins, which reflects a limitation of this qualitative readout.

We therefore aimed at assessing protein surface exposure by quantifying enzymatically released peptides of accessible proteins by trypsin cleavage of live bacteria in comparison to bacteria not treated with trypsin. We employed rather mild buffer conditions and quantitative enrichment as it has been used to identify protein-protein interactions^19,20^. We set out to evaluate a similar strategy employing quantitative data instead of qualitative data to account for background signal from cytosolic proteins. To address the effect metabolic activity of the bacteria we included PFA fixation in our screen. This allows separation of peptides enriched from surface cleavage from those associated with e.g. secreted vesicles of metabolically active bacteria.

Since prolonged trypsin cleavage correlates with increased cell death and a concomitant release of cytosolic proteins, we optimized cell surface peptide shaving for reduced cytosolic protein release (Supplementary Fig. 3). For this purpose, we made use of fluorescently labeled bacteria, as cytosolic protein leakage can be measured quantitatively in a high throughput format. We modeled different experimental variables such as incubation time and buffer compositions simultaneously using DoE with cell lysis as a quantitative readout (Supplementary Fig. 3).

The high sensitivity of our mass spectrometer pipeline quantifies even small amounts of proteins released by background processes during our optimized shaving procedure, as indicated by the large number of quantified proteins throughout all conditions and overlap of quantified proteins (Fig. 2A,C). Qualitative surface candidate selection is based on presence or absence of a protein in the sample rendering background lysis, which is a conceptual problem. In contrast our quantitative label free proteomics workflow enables us to determine even small protein abundance difference across samples^21^. Based on that workflow, we devised a stepwise selection process leveraging a high quantitative accuracy to distinguish most promising surface-vaccine candidate proteins from background.

Proteins on the surface of *H. pylori* are expected to increase throughout the trypsin treatment. In a first step, we performed correlation analysis (see material and methods: Correlation analysis) of 1153 quantified *H. pylori* proteins resulting in the identification of 146 putative surface proteins – the *H. pylori* surfome –that correlated with the time course (10 min, 20 min 30 min) for the trypsin treatment. A hierarchical clustering of the 146 surfome candidate proteins indicated two clusters (cluster 1 and 2 indicated in blue and purple, respectively) of overall 73 proteins with a high signal under the shaving conditions and consistently low signal for the trypsin-free control samples (Fig. 2D, Supplementary Fig. 1B). Notably, many proteins reach their maximum signal before the 30 min time point. Proteins that showed higher abundances for metabolically inactivated *H. pylori* in the control groups (without trypsin) were excluded (Supplementary Fig. 1B). In an annotation enrichment analysis of the 73 surfome proteins, we observed a significant enrichment of membrane proteins and depletion for cytosolic proteins (Fig. 2F) as it is expected for surface associated proteins.

Intriguingly, among these 73 proteins of the *H. pylori* surfome only 54 proteins were exclusively enriched in the presence of trypsin (Fig. 2D,E, cluster 1). The remaining 19 proteins (cluster 2) showed an increased abundance in the metabolically active (PFA −) control samples in addition to the strong signal for surface exposure (high in trypsin conditions) Fig. 2D,E, cluster 2). Based on that profile, we hypothesized that cluster 2 might comprise proteins on the surface of vesicles secreted by *H. pylori*.

To further narrow down the list of candidate proteins for an *in vivo* vaccine study, we prioritized candidates *in silico* similar to Moffit *et al*.^22^. To minimize cross reactivity towards other bacterial species and humans, we filtered for proteins that share less than 33% amino acid sequence identity to any other bacterial species and less than 5% homology to human proteins (Fig. 2G,H). To favor pan-protectivity within the *H. pylori* genus, we confirmed the identity of the remaining 22 candidates across 264 UniProt annotated *H. pylori* proteomes (Fig. 2I and Table 1).

**Table 1:** Final vaccine candidates derived from surfome analysis and homology prioritization. Cluster column denotes the enrichment pattern from which the proteins were selected (see Fig. 2E).

Uniprot ID	Gene name	Cluster
Q9ZJM9	jhp_1276	1
Q9ZL06	jhp_0776	1
Q9ZL62	jhp_0718	1
Q9ZLM7	jhp_0552	1
Q9ZLP8	jhp_0530	1
Q9ZLS6	jhp_0501	1
Q9ZLU0	cagN	1
Q9ZLU7	orf17	1
Q9ZLV5	orf8	1
Q9ZLV7	orf6	1
Q9ZLY2	jhp_0444	1
Q9ZM54	jhp_0369	1
Q9ZMP5	jhp_0173	1
Q9ZMT2	jhp_0136	1
P0A0V1	lpp20	2
Q9ZJF7	jhp_1355	2
Q9ZJY3	babB	2
Q9ZK39	jhp_1103	2
Q9ZKV2	babA	2
Q9ZL07	jhp_0775	2
Q9ZLV0	14	2
Q9ZMU8	jhp_0119	2

### Validation of surfome exposed vaccine candidates *in vitro* and *in vivo*

**Figure 3:**
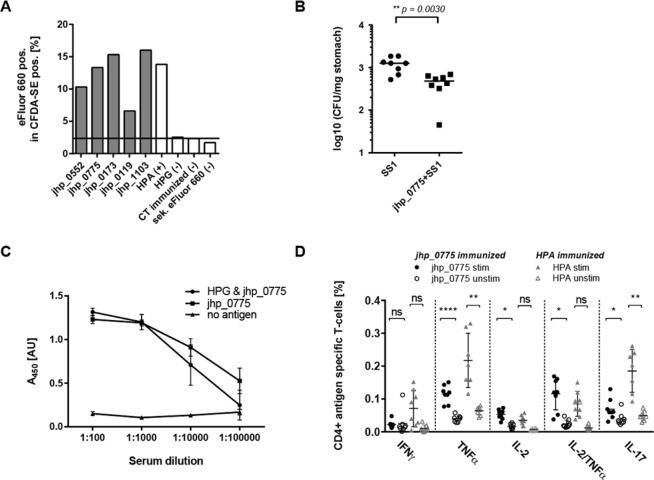
Confirmation of surface exposure of vaccine candidates and therapeutic efficacy of jhp_0775 in an *H. pylori* mouse infection model. (**A**) Staining intensities of vaccine candidates (grey bars) and controls (white bars) detected by purified antisera raised against the vaccine candidates and employed to stain the surface of CFDA-SE labeled *H. pylori*. Antibodies derived from HPA immunized mice served as positive control. The *H. pylori* gamma-glutamyltranspeptidase (HPG) served as additional control, as it is identified as periplasmic and secreted protein [68]. HPA and HPG were stained with high and low intensities, respectively, validating the experimental setup. (**B**) Mice were infected with *H. pylori* SS1 and immunized with jhp_0775 and cholera toxin (CT) or with CT alone as control. Gastric *H. pylori* colonization was determined and depicted as colony forming units (CFU) per mg stomach. (**C**) Antigen-specific serum IgG responses to jhp_0775 measured by ELISA. (**D**) CD4+T cells producing indicated cytokines were analyzed by flow cytometry before (filled, unstim) and after splenocyte restimulation (unfilled, stim) with jhp_0775 from mice immunized with CT as adjuvant and jhp_0775 and HPG as antigens (circles) as well as with HPA from mice immunized with CT as adjuvant and HPA and HPG as antigen (triangles). Data are shown as mean ± SD. p-values were determined by the nonparametric Mann-Whitney U test and the Šidák multiple comparison test for colonization levels and cytokine responses, respectively. Asterisks show significant differences between groups (****p < 0.0001, **p < 0.01, *<0.05).

As proof of concept, we next aimed at confirming the surface exposure and immunogenicity of selected identified vaccine candidates. We selected five vaccine candidates (jhp_0775, jhp_0173, jhp_0119, jhp_0552, jhp_1103) and recombinantly expressed and purified them from *E. coli* (Supplementary Fig. 4A–J). We immunized mice with the recombinant proteins, and used the purified polyclonal antisera (Supplementary Fig. 4K) to stain CFDA-SE (Carboxyfluorescein Diacetate Succinimidyl Ester) labeled bacteria (gating strategy see Supplementary Fig. 6A). All analyzed candidates showed robust staining frequencies by flow cytometry well above the mock-control or cytoplasmic proteins, confirming their surface exposure (Fig. 3A). Next, we selected the previously uncharacterized protein jhp_0775 for *in vivo* efficacy experiments because it exhibits the highest homology to all *H. pylori* strains (98.5%) and is among the 200 most abundant proteins in the *H. pylori* proteome (Supplementary Fig. 1). We immunized mice therapeutically with jhp_0775 to address to which extent the immune response in a chronic infection setting can be increased. To this end, we first infected BALB/c mice with *H. pylori* strain SS1 and then immunized them with jhp_0775 and cholera toxin (CT) as adjuvant. Four weeks after immunization, jhp_0775 immunized mice showed significantly reduced *H. pylori* colonization levels as compared to control (mock/CT) immunized mice (Fig. 3B). We quantified the humoral immune response by antigen-specific ELISA, and detected a strong increase in antigen-specific IgG levels after immunization (Fig. 3C). To characterize cellular immunity and quantify Th1 and Th17 immune responses, splenocytes from immunized mice were restimulated with recombinant jhp_0775 and the frequencies of CD4+ T cells producing the cytokines IFNγ, TNFα, IL-2 and IL-17 were measured by flow cytometry (Fig. 3D and Supplementary Fig. 6B). In agreement with a strong cellular immune response, we observed significantly increased levels of all cytokines except IFNγ. This cellular immune response was comparable to the response against HPA, a well-described *H. pylori* vaccine candidate known to induce T-cell responses in HPA immunized mice (Fig. 3D)^23^.

### Confirmation of immunogenicity in human samples

**Figure 4:**
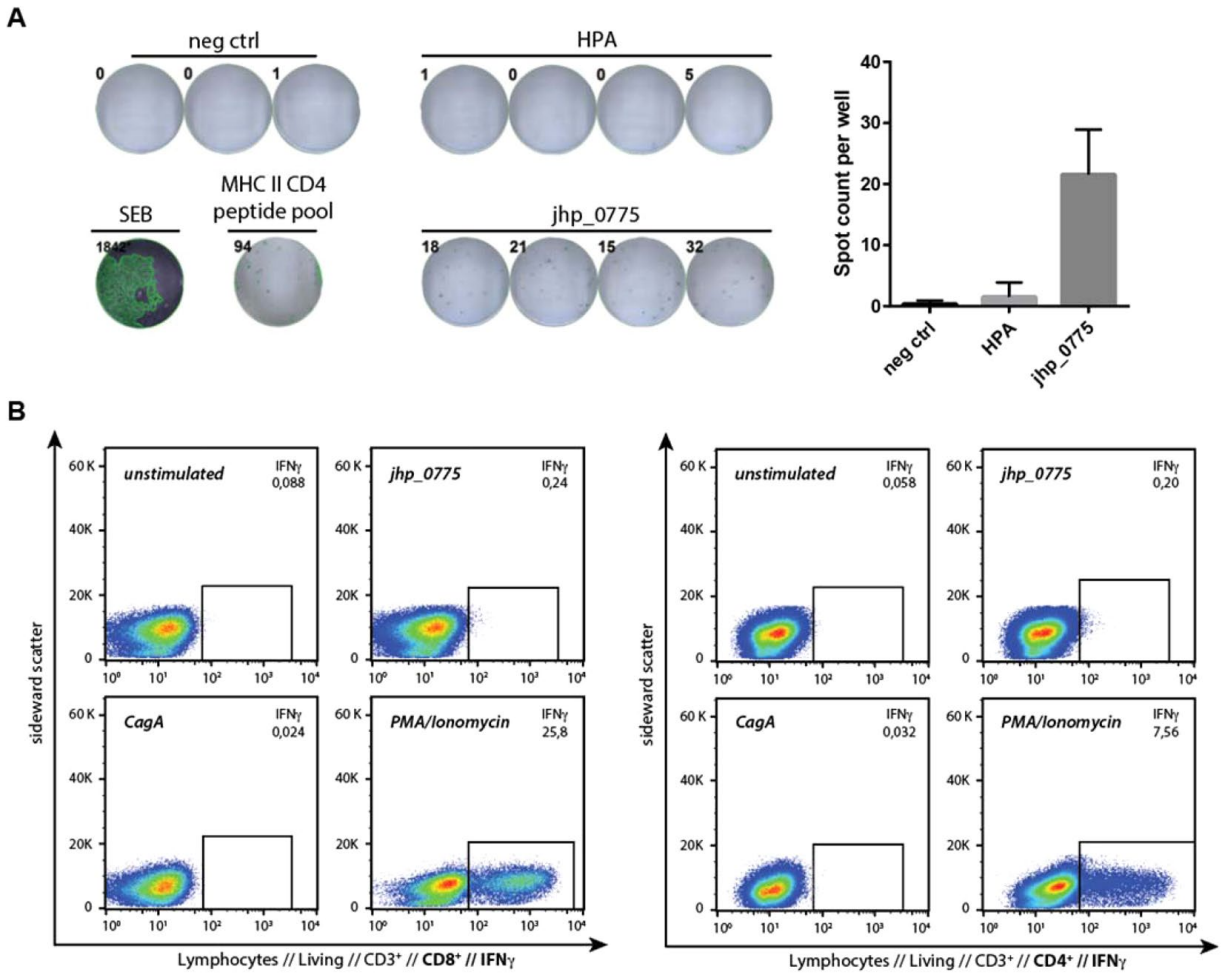
Immune response against jhp_0775 in humans. (**A**) ELISPOT analysis indicating cellular immune response against HPA and jhp_0775. Staphylococcal enterotoxin B (SEB) and MHC II CD4 peptide pool served as controls. The numbers at the top left of each well indicate the corresponding spot-counts per well. Neg ctrl, negative control without stimulation (**B**) Human PBMCs from infected patients were stimulated with jhp_0775, the highly immunogenic *H. pylori* antigen CagA and PMA/Ionomycin as positive control, and CD4^+^ and CD8^+^ IFNγ producing cells analyzed by flow cytometry. The percentage of IFNγ producing cells is indicated within each plot. PBMCs were not stimulated in the negative control, PMA/Ionomycin stimulated as positive control and stimulated with the highly immunogenic *H. pylori* antigen CagA as well as jhp_0775 to test the specificity of the IFNγ secretion.

To validate the immunogenicity of a panel of 5 selected candidates, and specifically jhp_0775, in human, we analyzed the humoral and cellular responses in samples from volunteers infected with *H. pylori*. While a strong immune response towards a specific antigen in the context of a chronic infection may indicate that such response does not achieve eradication of the pathogen, no immune response against a conserved antigen may suggest that the candidate protein is not immunogenic in human, or not properly presented. We therefore initially quantified the degree of a preexisting humoral response in infected humans by analyzing sera from a characterized human patient cohort with a LUMINEX^®^ bead-based assay (Table 2). FliD, a flagellum-associated protein, which was previously identified by our group and validated as a highly sensitive and specific biomarker for *H. pylori* infection, served as positive control^24^, as well as HPA, a surface protein which is often used as vaccine candidate^25^. Intriguingly, despite bacterial surface exposure of the antigens, most vaccine candidates showed only a low prevalence of antibody responses. In *H. pylori* infection, as well as for many other pathogens, the cellular immune system is key to mount an effective immune response, and efficacy of vaccination against *H. pylori* highly depends on T cell responses^26^. Thus, we tested the capacity of our selected candidate jhp_0775 to induce a cellular response in PBMCs derived from infected patients by measuring IFNγ secretion in an ELISpot assay (Fig. 4A). Upon restimulation with recombinant jhp_0775, one out of four tested patients showed a significant number of IFNγ secreting cells. This patient reacted specifically to jhp_0775, while not reacting to HPA, a protein known to contain T cell epitopes^27^. As expected, the unstimulated negative control did not show any spots, while the Staphylococcal enterotoxin B (SEB) and MHC class II CD4 peptide pool-stimulated positive controls confirmed the functionality of the immune cells and CD4 memory T cells, respectively. Therefore, as a proof of concept for our approach, jhp_0775 is recognized *in vivo* and induces antigen-specific cellular responses in infected patients, but is not readily detected in all infected patients. Several cell types are capable of producing IFNγ upon stimulation by antigen presenting cells. To further dissect the capacity of jhp_0775 to trigger a cellular immune response, we analyzed the cellular origin of human PBMC-derived IFNγ secretion using intracellular cytokine staining. PBMCs from the ELISpot reactive donor showed a specific increase in IFNγ producing CD4^+^ and CD8^+^ T cells in flow cytometry, in comparison to the non-reactive donor (Fig. 4B; gating strategy: Supplementary Fig. 6C). Thus, *in vivo*, both CD4^+^ and CD8^+^ T cells are capable of responding to jhp_0775-derived epitopes after infection as well as vaccination.

**Table 2:** Humoral immune response measured by Luminex^®^ multiplex bead assay.

Uniprot ID	Gene name	positive n = 378	negative n = 299	Sensitivity (Prevalence)	Specificity
Q9ZLM7	jhp_0552	145	265	38.4	88.6
Q9ZMP5	jhp_0173	164	258	43.4	86.3
Q9ZL07	jhp_0775	141	261	37.3	87.3
Q9ZMU8	jhp_0119	144	256	38.1	85.6
Q9ZK39 (HopQ)	jhp_1103	227	263	60.1	88.0
Q9ZL91 (FliD)	jhp_0689	361	274	95.5	91.6
Q9ZL47 (HPA, HpaA)	jhp_0733	319	257	84.4	86.0

The specificity (negative sera) and sensitivity (prevalence of antibody responses in positive sera) show the percentage of true negatives and true positives, respectively. Positivity-cutoffs for each antigen were defined at 2.5 times or 9 times (for jhp_0689) their standard deviation of the negative median fluorescence intensity (MFI).

In summary, our novel vaccine discovery pipeline, which combines quantitative MS, computational candidate characterization and selection, as well as *in vivo* validation, revealed several proteins as promising *H. pylori* vaccine candidates. In mice, jhp_0775 significantly reduced colonization levels after immunization correlating with an increased humoral and cellular immune response. In humans, jhp_0775 is capable of eliciting a cellular immune response leading to IFNγ producing CD4^+^ and CD8^+^ T cells, both of which are considered important correlates of protection.

## Discussion

The history of vaccines is a success story. Vaccines led to the eradication of smallpox and significantly reduced the number of fatalities from other diseases like measles, tetanus, diphtheria and pertussis. However, effective vaccination strategies are still missing for many infectious diseases with major clinical and socioeconomic impact. Moreover, spreading antibiotic resistance escalates the demand for effective disease prevention and underlines the importance of vaccination. Therefore, effective and fast strategies to screen for novel vaccine candidates are needed. This is particularly important for gram-negative pathogens, which dominate the WHO list of priority pathogen threads, but for which no vaccines are available.

To select the most promising targets from thousands of bacterial proteins, a promising strategy is to focus on proteins on the exposed surface of the pathogen^7^ or, in case of intracellular pathogens, on the host cell^8^. A variety of protocols have been developed over the years for isolating proteins exposed on bacterial surfaces. These surfome approaches have been applied to identify potential vaccine candidates^7,28^. A remaining challenge is to provide a generic and adaptable strategy that accounts for background lysis, especially of gram-negative bacteria, which are more sensitive to lysis^9^.

The high dynamic range and high abundance of contaminating cytosolic proteins require labeling- and orthogonal enrichment strategies or bioinformatics filtering e.g. based on prediction of signal peptides or localization to eliminate proteins derived from cell lysis^7^. While a missing experimental dissection of proteins derived from background lysis increases the amount of false positive (not surface-exposed) proteins, computational filtering is usually associated with false negatives as many proteins are excluded based on incomplete or incorrect annotations in public domain databases. Consequently, and in contrast to our strategy, previous attempts favor the identification of very few highly abundant surface proteins^28,29^.

Our pipeline facilitates an easy adaption to screen other pathogens for vaccine candidates. A DoE based optimization strategy generically improves the surfome mapping while reducing background lysis, thereby providing conceptual advantages to common strategies^30^. In addition, we outline a strategy to discriminate surface-exposed from secreted proteins by comparing the gradual increase of proteins in metabolically active bacteria (PFA − in this study) to those that are inactivated (PFA+). However, additional studies separating vesicles by filtration or density centrifugation are required to test that hypothesis. In comparison to widely used cell surface biotin labelling^15–18^, our approach results in lower cytosolic background detection *for H. pylori* and presumably also other gram-negative pathogens. However, DoE based experimental optimization of surface biotinylation with minimal co-labeling of cytosolic proteins may present an interesting future research strategy.

In our surface shaving strategy, we use trypsin to digest proteins on the bacterial surface. Cleaving after arginine and lysine residues, trypsin produces peptides with an average length of 14 amino acids. The advantage is that these peptides have a high chance of a unique sequence within the proteome^31^. A proteomics analysis of the bacterial surface may, however, be biased toward proteins exhibiting trypsin cleavage sites since arginine and lysine – the amino acids after which trypsin cleaves – are not evenly distributed^32^. We and others have previously shown that a combination of multiple enzymes presents an experimental strategy to overcome this bias^33–35^. Although our surfome approach does not require complete protein sequence coverage as differential protein abundances are inferred from pairwise peptide comparisons by the MaxLFQ algorithm^21^, it would be interesting for future studies to employ multiple enzymes to map exposed protein domains more comprehensively.

A potential limitation of this study is that *H. pylori* is exposed to very harsh conditions in the host’s stomach and might remodel the proteome *in vivo*. Since we overall have a very high coverage of close to 80% of the *H. pylori* proteome (80% of membrane proteins) we do not expect to miss a large proportion of candidate proteins. However, we cannot exclude that some proteins, in particular those that are important in immune evasion, are only expressed in the host environment. It would be very interesting to study *H. pylori* exposed to conditions mimicking the human stomach.

The experimental determination of sequence-conservation and commonality of antigens among strains presents a common challenge for achieving broad cross-protection. Our computational filtering strategy for conservation increases the success towards identifying suitable vaccine candidates for three reasons. First, it increases the likelihood to achieve pan protection, which is desirable in particular for pathogens with high genetic variability such as *H. pylori*^36^. Second, highly conserved proteins within the genus are presumably essential for specific bacterial pathogenesis. Third, low conservation compared to other species will reduce the risk of inducing an immune response against commensal bacteria. In any case, limited accessibility of surface proteins due to mucoid capsules, polysaccharide side chains and glycolipids present in many gram-negative bacteria should be considered and experimentally excluded, as done in this report by surface staining with specific antisera.

A vaccine candidate suitable for clinical development should be efficacious in animal models and be able to trigger B- and T-cell immune responses in humans. Especially for *H. pylori*, a broad immune response comprising B- and T-cell responses seems necessary for protection^37^. We first showed in a mouse vaccination study that the antigen jhp_0775 induces specific B and T cell responses and significantly reduces colonization levels, validating the protective capacity of this antigen. However, targeting antigenic surface proteins may not be sufficient to prevent or eradicate infection due to complex host immune responses, where the effectiveness of vaccine candidates will depend on the interaction of host immune cells, antibodies, replicating organisms, and evasion of host defenses by the pathogen in chronic infections. It must be noted that rodents are not the natural hosts for *H. pylori*, and “artificial” colonization requires relatively high doses of bacteria which were adapted to the mouse^38^. Thus, in immunization studies, sterility is almost never observed, and a reduction of colonization by 1 log is usually considered as protection^39^. The lack of IFNγ in our model can be attributed to the inbred BALB/c mouse strain, in which IFNγ responses are generally low with the utilized immunization strategy^40^. Indeed, when we assessed the T cell responses in humans, CD4+ and CD8+ T cells derived from infected patients secreted IFNγ as shown by ELISPOT and ICCS. On the other hand, the selected antigens revealed low to medium sero-reactivity in a well-characterized human cohort.

Such low but detectable baseline sero-reactivity and T cell response is a desired property of novel vaccine candidates in the context of chronic infections. In chronic infections, the immune system is not capable to eradicate the pathogen, which in part could be mediated by two functionally distinct protein classes: (1) proteins that are exposed on the bacterial surface but are only weakly recognized by the host immune system and (2) proteins that actively attenuate the host’s immune system. In both cases these proteins contribute to the immune evasion and thus, to establish and maintain a chronic infection. Specifically targeting the immune system to proteins derived from these two classes could provide an unexploited potential to eradicate pathogens causing chronic infections.

In summary, we established a streamlined combination of a DoE guided shaving proteomics with a computational candidate prioritization and functional immunogenicity assays, to identify jhp_0775 as a novel *H. pylori* vaccine candidate. Our study thereby provides a generic roadmap to facilitate a rapid vaccine candidate discovery with widespread applications for other bacterial pathogens.

## Methods

### Recombinant protein production in *Escherichia coli*

#### Preculture

Chemically competent *E. coli* BL21(DE3) were transformed with the production plasmid, plated on LB plates containing the suitable antibiotic and incubated until single colonies were visible. A single colony was picked to inoculate LB medium containing the suitable antibiotic. The culture volume was at least 1/50th of the production culture. After incubation overnight at 37 °C under vigorous shaking, the main production culture was inoculated to an OD_600_ of 0.1 and incubated depending on the expression system and induction method employed.

#### T7 promoter system using autoinduction

Autoinduction was performed essentially as described by (Studier 2005). In brief, after inoculation, bacteria were grown at 37 °C with 250 to 275 rpm (shaking diameter of 2,5 cm) - trying to ensure a maximum oxygen transfer rate for high cell density growth^41^- in auto-inducing terrific broth (TB) medium supplemented with 2 mM MgSO_4_, 100 mg/L Kanamycin-Sulfate (Carl Roth), 0.2 g/L PPG2000 (Sigma Aldrich) and 0.2% (w/v) Lactose-monohydrate (Sigma Aldrich), until an OD_600_ of 1–2 was reached. Afterwards, the temperature was lowered to 25 °C and auto-induced overnight, typically reaching a final OD_600_ of 10 to 15 the following morning. To facilitate insoluble protein production, the induction temperature was left unchanged at 37 °C. Bacteria were harvested by centrifugation at 6000 g for 15 min at 4 °C using an SLA-3000 rotor in a pre-cooled Sorvall RC-6 Plus centrifuge (Thermo Fischer) and either directly processed for protein purification or scraped into sealable plastic bags and stored at −20 °C.

#### T7 promoter system using IPTG induction

Bacteria were grown as described for autoinduction but with minor modifications. The TB medium did not contain Lactose-monohydrate. After reaching an OD_600_ of 2–3, the temperature was lowered to 25 °C, incubated for another 1 to 1,5 hours and subsequently induced by addition of 1 mM Isopropyl-β-D-thiogalactopyranoside (IPTG, stock concentration 1 M).

#### Tet promoter system using AHT induction

Proteins were produced as described for IPTG, but using 200 µg Anhydrotetracycline hydrochloride (AHT) per liter of culture as inductor (stock concentration 2 mg*ml^−1^ in ethanol).

### Recombinant protein purification

#### Cell disruption & feed preparation

Bacteria were resuspended in 10 ml cold NiNTA buffer A (100 mM Tris, 500 mM NaCl, pH 8.0) per gram of biological wet weight (BWW), supplemented with 0.1 mM AEBSF-HCl, 150U/g BWW DNase I and 5 mM MgCl_2_ with an Ultra-Turrax T25 digital (IKA) at 10 000 to 14 000 rpm for 30 to 60 seconds. Cell disruption was performed by high-pressure homogenization with a PANDA2000 (GEA Niro Soavi) at 800–1200 bar in 3 passages at 4 °C. The cell lysate was clarified by centrifugation at 25000 g for 30 min at 4 °C in an SLA-1500 rotor. If supernatants contained the recombinant protein, remaining particles were removed by filtration with a 0.2 µM filter. If the protein was located in the pellet, the supernatant was discarded and the pellet resuspended with an Ultra-Turrax T25 digital (IKA) at 10 000 to 14 000 rpm for 30 to 60 seconds in ten volumes per gram pellet “NiNTA unfold red A” (100 mM Tris, 500 mM NaCl, 6 M GuaHCl, 1 mM DTT, pH 8.0) buffer, containing guanidine hydrochloride as denaturing and DTT as reducing agent. Then, the solution was stirred for 30 minutes at 4 °C and clarified by centrifugation at 25000 g for 30 min at 4 °C.

#### Chromatography setup

For chromatography, an automated FPLC system was used at 2 to 8 °C (ÄKTA^TM^ avant 25, GE Healthcare). A standardized inlet system was developed, using inlet A5, A6 and A7 for 20% ethanol, 1 M NaOH and H_2_O, respectively, and inlet B5, B6 and B7 for 20% ethanol with 0.2 M acetate, 2 M NaCl and H_2_O, respectively. Outlet 1 was connected to a nickel waste container. This setup facilitates two functions: almost any column can be cleaned in place (CIPed) right after use without switching buffers using universally applicable CIP programs; NiNTA chromatography can be automated for sequential runs of different proteins. Therefore, this setup allows sequential, automated and unattended multiple protein purification.

#### NiNTA chromatography from cell lysate supernatants

Proteins were purified by consecutive nickel affinity and size exclusion chromatography. Briefly, the clarified cell lysate was loaded onto a 5 ml pre-packed NiNTA HisTrap FF crude column (GE Healthcare) pre-equilibrated with NiNTA buffer A, washed with ten column volumes (CV) of NiNTA buffer A mixed with 2% NiNTA buffer B (100 mM Tris, 500 mM NaCl, 500 mM Imidazole, pH 8.0) and the bound protein eluted with a 15 CV linear gradient to 75% NiNTA buffer B. Eluted peak fractions were collected, pooled and concentrated to a final concentration of 3 to 10 mg*ml^−1^ using a 10 kDa molecular-weight cutoff spin concentrator. After each use, columns were completely stripped according to manufacturer’s instructions, cleaned in place (CIP) by 5 CV 1 M NaOH with a residence time of at least 30 minutes, recharged with NiSO_4_ and stored in 20% ethanol. All nickel-containing liquids were collected in a special waste flask.

#### NiNTA chromatography from insoluble protein fractions

Insolubly produced proteins were purified as described for soluble proteins with minor changes. Before loading the DTT containing supernatant, the HisTrap FF crude column (GE Healthcare) was prepared for reducing conditions according to the manufacturer’s instructions, as care has to be taken to remove all non-chelated nickel ions. Briefly, the column was equilibrated with 5 CV NiNTA buffer A, blank eluted with 5 CV NiNTA buffer B, and re-equilibrated with 5 CV “NiNTA unfold red A”. Then, the feed was loaded, washed and eluted as described above, except using “NiNTA unfold red B” (NiNTA unfold red A + 500 mM Imidazole) as elution buffer. Eluted peak fractions were collected, pooled and concentrated to a final concentration of at least 10 mg*ml^−1^ using a 10 kDa molecular-weight cutoff spin concentrator before refolding by rapid dilution.

#### Protein refolding by rapid dilution

After denaturing NiNTA chromatography and concentration, unfolded and reduced proteins were refolded by rapid dilution. The refolding buffers used herein contain high amounts of arginine - an efficient enhancer of protein refolding (Baynes *et al*. 2005; Arakawa *et al*. 2007; Tischer *et al*. 2010) - with DTT maintaining reducing conditions. After protein concentration, a 20-fold excess of pre-cooled refolding buffer (330 mM ArgPO_4_, 10 mM DTT, pH 7.4) was placed on a magnetic stirrer. At 4 °C, the protein was slowly added dropwise to the refolding buffer under slight stirring, incubated for 45 minutes and sterile filtered. The filtrate was reapplied to a NiNTA column as described above with minor changes. The column was prepared for reducing conditions with re-equilibration by “NiNTA reducing A” (NiNTA buffer A + 5 mM DTT), the refolded protein loaded and washed by NiNTA buffer A with a subsequent residence time of 45 minutes, allowing controlled oxidation of disulfide bonds. Afterwards, the protein was eluted with NiNTA buffer B, pooled, concentrated and polished by size exclusion chromatography.

#### Size exclusion chromatography

5 ml of the NiNTA purified and concentrated proteins were loaded onto a HiLoad 16/600 Superdex 75 pg or 200 pg column (GE Healthcare), pre-equilibrated with formulation buffer (330 mM arginine/H_3_PO_4_, pH 7.4) and eluted at a flow rate of 1 ml*min^−1^. Finally, protein containing fractions corresponding to a monomer peak or a defined multimer peak were pooled, concentrated to at least 1 mg*ml^−1^ and stored at −20 °C or −80 °C. For calibration, the gel filtration calibration kits were used according to manufacturer’s instructions (GE Healthcare). The resulting calibration curve values were fitted and the resulting equations used for subsequent molecular weight calculation. After purification of a protein, the column was CIPed by 0.5 to 1 CV 1 M NaOH with a residence time of at least 60 minutes, washed with 2 CV of H_2_O and stored in 20% ethanol.

#### Polyclonal antibody purification from serum

Polyclonal IgG antibodies were purified from mouse serum by Protein A affinity chromatography. Therefore, a 1 ml MabSelect^TM^ SuRe column (GE Healthcare) was equilibrated with 5 CV of Protein A buffer A (20 mM Na_2_HPO_4_, 140 mM NaCl, pH 7.2). Serum from one mouse was diluted to 10 ml in Protein A buffer A, particles removed by sterile filtration and applied to the column with a residence time of 2 to 3 minutes, ensuring greater dynamic binding capacity. Then, the column was washed with 15 CV of Protein A buffer A. Bound antibodies were eluted isocratically with a step to 100% Protein A buffer B (100 mM citric acid, pH 3.0). To neutralize the pH of the elution buffer, 200 µl of 1 M Tris pH 9.0 per 1 ml of eluted fraction were pre-filled into a 96 deep well collection plate. Afterwards, fractions containing the antibodies were pooled, dialyzed against PBS, concentrated to 2 mg*ml^−1^ and stored at −80 °C.

#### SDS-PAGE

Gels were casted into 1.0 mm Novex^®^ gel cassettes (Life Technologies) consisting of a separating gel (8 to 15% (w/v) acrylamide, 375 mM Tris-HCl pH 8.8, 0.1% SDS, 0.1% APS and 0.1% TEMED) and a stacking gel (5% (w/v) acrylamide, 125 mM Tris-HCl pH 6.8, 0.1% SDS, 0.1% APS and 0.1% TEMED) Protein samples were prepared by adding 4x sample buffer and heating at 95 °C for 5 minutes. For recombinant proteins, 1 to 10 µg per lane was applied to estimate purities. Gels were placed into an XCell SureLock™ Mini-Cell electrophoresis system chamber (Life Technologies) and proteins separated at 170 V for 90 to 120 minutes. Alternatively, the samples were prepared and separated using the Bolt^®^-system (Thermo Fisher Scientific) with 4–12% Bis-Tris Plus precast gels. Subsequently, gels were stained either with Coomassie staining solution, having a detection limit of approximately 100 ng per band (Shevchenko *et al*. 1996), or with Quick Coomassie™ Stain (Serva Electrophoresis GmbH, Heidelberg, DE), having a detection limit of 5 ng per band.

#### Quantification of endotoxin content

The endotoxin content of recombinantly produced proteins was determined using the EndoLISA^®^ ELISA-based Endotoxin Detection Assay (Hyglos) according to manufacturer’s instructions. Fluorescence was measured using an Infinite^®^ F200 pro fluorescence reader (Tecan) equipped with a 380/20 nm and a 465/35 nm excitation and emission band-pass filter, respectively. To ensure measurements within the standard curve, proteins were diluted 10, 100, 1000 and 10 000-fold with endotoxin-free water. For data analysis, a sigmoidal curve fit was used to fit the standard curve, where fluorescence values ranging from 0.05 to 100 EU*ml^−1^ were within the dynamic range.

#### Luminex® analysis of patient sera

The Luminex^®^ assay was used to measure the serological IgG response of human serum samples to the vaccine candidates. Antigens were dialyzed against a theoretical buffer excess of >1000 against an amine free buffer (20 mM NaP_i_, 500 mM NaCl, pH 7.4) to enable subsequent immobilisation to carboxylated MagPlex microspheres using a standard EDC-NHS coupling strategy. The concentration of the antigens was greater than 0.1 mg*ml^−1^. Antigen immoblilisation and Luminex^®^ multiplex bead array experiments were essentially performed as described previously (Filomena *et al*. 2015; Planatscher *et al*. 2013), using a well characterized patient cohort of *H. pylori* 378 positive and 299 negative sera according to urea breath test and histology.

#### Murine infections and immunizations

Mouse strains and housing. Six to eight-week old female BALB/c mice were purchased (Harlan Winkelmann GmbH) and housed at the MIH (Technische Universität München, Medical Microbiology, Immunology and Hygiene, Munich, Germany) under specific pathogen free conditions. All animal experiments were conducted in compliance with European guidelines for the care and use of laboratory animals. Experiments were approved by the animal welfare committee of the “Regierung von Oberbayern” (Az. 55.2-1-54-2532-107-16).

Experimental infection with *H. pylori*. *H. pylori* infections were essentially carried out as described previously (Semper *et al*. 2014). Briefly, four to six hours before infection, mice were fasted. 1 × 10^9^ *H. pylori* SS1 were suspended in 200 µl BB medium supplemented with 10% FCS and were given orogastrically in a volume of 200 µl three times at an interval of two days. Groups of eight mice were used. Upon termination, mice were sacrificed by CO_2_ inhalation.

Quantitative assessment of colonization. Colonization was quantified by detaching *H. pylori* from murine stomachs, plating and counting the colony forming units (CFU). Therefore, stomachs were opened and residual chow removed by washing in PBS. Afterwards, a piece of 15 to 30 mg was removed, immediately immersed in 1 ml BB medium supplemented with 10% FCS. Bacteria were detached from the stomach by vortexing for 5 minutes. Subsequently, 100 µl of supernatant was plated directly (1:10) on WC-dent special plates and another 100 µl serially diluted, additionally plating dilutions of 1:100 and 1:1000. Afterwards, plates were incubated under microaerophilic conditions for five days at 37 °C before counting CFU. Plates containing one to 400 colonies were considered for counting, usually spanning two dilutions, and the corresponding mean of CFU per mg stomach tissue calculated.

Immunogenicity of vaccine candidates. The immunogenicity of vaccine candidates was tested and antiserum raised by immunizing wild type BALB/c mice *intraperitoneally* (i.p.) with 30 µg antigen and 10 µg Cholera Toxin (CT, Sigma Aldrich) as adjuvants four times at an interval of one week. Antigens and adjuvants were administered in a volume of 200 µl diluted in formulation buffer. One week after the last immunization, mice were sacrificed and blood was withdrawn with needles pretreated with heparin. The collected blood was centrifuged at 10 000 g for 10 minutes and the plasma collected. The plasma was either stored at −20 °C or directly used to purify the antibodies.

Therapeutic efficacy of vaccine candidates. To test the efficacy of selected vaccine candidates, mice were vaccinated in a therapeutic setup. Wild type female BALB/c mice were infected orally by 10^8^ *H. pylori* SS1 on day 0, 2 and 4. Subsequently, the animals were immunized orally on day 28 and orally combined with i.p. on day 35 and 42. For oral immunization with a single antigen, 100 µg were administered combined with 10 µg CT, whereas 30 µg for each antigen was used in case of combining multiple antigens. For i.p. immunization, 30 µg for each antigen was administered with 10 µg CT. Antigens and adjuvants were administered in a volume of 200 µl diluted in formulation buffer (330 mM ArgPO4, pH 7.4). On day 70, mice were sacrificed, bacteria extracted from the stomach and plated on agar plates.

### Immunochemical methods

#### Western blot

Western blot was used to specifically detect affinity-tags or test functionality of immune serum raised against the recombinantly produced proteins. After SDS-PAGE and equilibration in Semi dry blotting buffer, the gel was placed between soaked Whatman paper onto a methanol activated 0.45 µM PVDF-membrane. After removing air bubbles, proteins were blotted in a Trans Blot^®^ SD semi-dry transfer cell (Bio-Rad) with 2 mA per cm^2^ for 110 minutes. Subsequently, membranes were blocked with TBS-T supplemented with 5% (w/v) skimmed milk powder for 1 hour, washed, primary antibody added in TBS-T supplemented with 1% (w/v) skimmed milk powder and incubated for 1 hour at room temperature or at 4 °C overnight. After washing, a secondary antibody-HRP conjugate was added, incubated for 1 hour at room temperature and washed. Washing in between incubations steps was carried out four times with TBS-T for 10 minutes. For detection, ECL Western Blotting Substrate (Pierce Biotechnology) was prepared according to manufacturer’s instructions and evenly spread onto the membrane. After 1-minute incubation, the signal was detected incrementally by either photographic film or in the ChemoCam ECL Imager (Intas) CCD-imager at incubation times spanning 30 seconds to 10 minutes.

#### ELISA

For analyzing the humoral immune response by antigen specific ELISA, the corresponding antigen (1 µg*ml^−1^) in PBS was coated onto a MaxiSorb^TM^ 96-well microtiter plate (Thermo Fischer) at 4 °C overnight. Subsequently, wells were blocked with SmartBlock (Candor), serum added in a serial dilution ranging from 1:100 to 1:100000 and incubated for 1 hour at 37 °C. Next, a secondary anti-mouse-IgG-HRP conjugate (AbD Serotec) was added in a dilution of 1:10000 and incubated for 1 hour at 37 °C. After incubation, TMB substrate solution was added and the enzymatic reaction stopped with 2 N H_2_SO_4_ after 3 minutes. Washing in between incubation steps was carried out four times with PBS/0.05% Tween20. Absorbance was detected at 450 nm with background correction at 620 nm in an Infinite F200 Pro (Tecan).

### Flow cytometry analysis

#### Intracellular cytokine staining - mouse

Antigen-specific cellular immune response was analyzed by intracellular cytokine staining (ICCS) of restimulated splenocytes from immunized and control groups. Spleens were transferred into 3 ml RPMI+ medium and kept on ice until preparation was finished. Spleen single cell suspension was prepared using a 70 µM cell strainer. For erythrocyte lysis, cells were incubated in 3 ml ACT buffer (17 mM ammonium chloride, 153 mM Tris) for 7 minutes. After washing, cells were resuspended in 8 ml medium. 1 ml cell suspension was restimulated with 75 µg of antigen in a 24-well plate for 2 hours at 37 °C. Subsequently, GolgiPlug^TM^ was added overnight to suppress cytokine secretion, leading to intracellular cytokine accumulation. For cell staining, cells were first labeled with EMA to enable live/dead discrimination. After washing, the surface marker CD4 was stained. After permeabilization with Cytofix/Cytoperm™ Plus (BD Biosciences), intracellular cytokines were stained with an antibody-panel against IFNγ, TNFα, IL-2 and IL-17. Subsequently, cells were fixed with 1% PFA, filtered and analyzed by flow cytometry on a CyAN ADP 9 color analyzer (Beckman Coulter), gating on living lymphocytes positive for CD4, counting at least 100 000 events. Single color controls were used for live compensation. Data were analyzed with the FloJo X software (Treestar).

#### Intracellular cytokine staining - human

Blood samples were taken from healthy and *H. pylori* infected patients after informed consent was obtained, in accordance with ethical regulations (approved by the ethical committee of the faculty of Medicine TUM # 5662/13). PBMCs were isolated by Ficoll^®^ gradient and cryopreserved in 10% DMSO from. Cells were quickly thawed, resuspended in pre-warmed PBS, centrifuged at 700 g for 10 minutes and resuspended in 5 ml AIM-V T cell medium. After counting, cell concentration was adjusted to approximately 1 × 10^7^ cells per ml and 1,5 × 10^6^ cells seeded into a round bottom 96-well plate. For restimulation, 50 µg*ml^−1^ antigen with an endotoxin content of less than 30 EU*mg^−1^ was added and incubated for 1 hour at 37 °C. Subsequently, GolgiPlug^TM^ was added (1:500) to suppress cytokine secretion, leading to intracellular cytokine accumulation, and incubated for 3.5 hours. As controls, cells were left untreated or stimulated with PMA/Ionomycin.

For cell staining, Fc receptors were blocked with the Fc receptor blocking reagent (Miltenyi Biotech). Then, cells were labeled with Fixable Viability Dye eFluor^®^ 506 (eBioscience) to enable live/dead discrimination. After washing, the surface markers CD3, CD4 and CD8 were stained. Then, cells were fixed with 4% PFA, washed twice in FACS buffer and stored at 4 °C overnight. On the next day, cells were permeabilized with Cytofix/Cytoperm™ Plus (BD Biosciences) and intracellular IFNγ was stained. Subsequently, cells were washed twice, filtered and analyzed by flow cytometry on a CyAN ADP 9 color analyzer (Beckman Coulter), gating on living lymphocytes positive for CD3, counting at least 100 000 events. Single color controls were used for post-acquisition compensation. Data were analyzed with the FloJo X software (Treestar).

#### Surface protein staining of *H. pylori*

Serum from immunized mice was prepared from whole blood, further purified by Protein A affinity chromatography and isolated polyclonal IgG antibodies adjusted to a concentration of approximately 2 mg*ml^−1^. *H. pylori* J99 were CFDA-SE labeled, washed and blocked with heat inactivated FCS for 15 minutes at 37 °C under shaking. Subsequently, bacteria were washed with PBS with 0.5% (w/v) BSA, adjusted to an OD_600_ of 0.5 and 100 µl seeded into a round bottom 96-well cell culture plate. Purified antibodies were diluted in PBS with 0.5% (w/v) BSA. Bacteria were pelleted by centrifugation, the supernatant discarded and the pellet resuspended with 100 µL the corresponding primary antibody dilution and incubated for 30 min on ice. After washing twice, the eFlour^®^660 labeled secondary anti-mouse IgG1-HRP conjugate (eBioscience) was added and incubated for 30 minutes on ice. After washing twice, bacteria were fixed with PBS with 1% PFA, filtered and analyzed by flow cytometry on a CyAN ADP 9 color analyzer (Beckman Coulter), gating on CFSE positive cells. The experiment was controlled by antibodies derived from the following immunizations: HPA (UniProt ID B5Z7F9), an outer membrane protein of *H. pylori*; HPG (UniProt ID O25743), a protein located in the cytoplasm and inside of outer membrane vesicles; CT without antigen. Data were analyzed with the FloJo X software (Treestar).

#### ELISpot

IFNγ ELISpot analysis was performed with the human T-Track^®^ basic IFNγ kit (Lophius Biosciences) according to manufacturer’s instructions. In brief, PBMCs were isolated, cryopreserved and thawed as described for human ICS. In total, 6 × 10^5^ cells were seeded onto pre-coated 96 well stripes. For restimulation, 50 µg*ml^−1^ antigen with an endotoxin content of less than 30 EU*mg^−1^ was added. As positive control, the *Staphylococcus* enterotoxin B (SEB) was used, demonstrating cell functionality. In addition, 5 µg*ml^−1^ of an EFT-MHC Class II control peptide pool (Cellular Technology Limited) was used to specifically stimulate CD4 memory T cells. Cells were incubated for 20 hours at 37 °C. IFNγ was detected and quantified by a CTL-ImmunoSpot^®^ S6 FluoroSpot Line reader (Cellular Technology Limited). Quality control and background subtraction were done by CTL ImmunoSpot^®^ Academic Software.

### Surfome preparation and quantitative mass spectrometry

#### Design of experiments

In experiments, the measured outcome typically depends on multiple variables. To measure their impact, scientists usually employ the one factor at a time (OFAT) approach, changing one variable at a time while holding all others constant. But this approach requires a lot of experiments, and does not quantify intervariable and higher-order dependencies. Here, the Design of Experiments (DoE) approach offers a solution^42^. Experiments are mathematically planned and evaluated, reducing the number of experiments while quantifying the impact of all variables and their dependencies on the readout. In the last decade, DoE has become increasingly popular in the field of chromatography^13^.

Here, DoE was employed to assess the impact of buffer components and time of incubation on autolysis by the CFDA-SE labeling assay. The software MODDE (Umetrics, Malmö, SE) was used for experimental planning, analysis and subsequent visualization. For response surface optimization (RSM) the suggested experimental design central composite face centered (CCF) with a star distance of 1 was chosen. The experiment included 19 conditions, including 5 center points, and was executed in the randomized order as suggested by the software. For evaluation, insignificant terms were removed until the model was not further improved. The experiment was valid for interpretation when R^2^, Q^2^, model validity and reproducibility were greater than 0.5, 0.2, 0.25 and 0.6, respectively. In case of very high reproducibility, a negative value for the model validity was accepted, as this represents an artefact if all other values match the criteria^43^.

### CFDA-SE labeling and autolysis assay

#### E. coli

*E. coli* DH5α were transformed with pET30b to confer kanamycin resistance, plated on LB-kanamycin plates and incubated overnight at 37 °C. A single colony was picked and grown in 5 ml LB-kanamycin overnight. The preculture was inoculated 1:200 and expanded in 1 l LB-kanamycin under shaking at 200 rpm at 37 °C until reaching an OD_600_ of 1.0. Cells were harvested by centrifugation at 4400 g for 15 minutes at room temperature, washed twice and resuspended in PBS, adjusting an OD_600_ of 20.

CFDA-SE labeling was performed as described previously with minor modifications (Logan *et al*. 1998). In brief, a 10 mM stock of CFDA-SE dissolved in DMSO was added to a final concentration of 5 µM. As background control, only DMSO was added. Then, bacteria were incubated at 37 °C for 20 minutes at 200 rpm protected from light, washed once in PBS, split into 1 ml aliquots and transferred into 1.5 ml reaction tubes.

For DoE experiments, buffers were prepared in the meantime. The bacteria were centrifuged at 5000 g for 5 minutes. The pellets were treated according to the DoE worksheet, providing the experimental conditions for the resuspension buffer, resuspension sequence and time of incubation. Afterwards, bacteria were pelleted by repeated centrifugation and the supernatant withdrawn, taking care not to disturb the pellet. The supernatant was transferred into white 96 well assay plates. Finally, the fluorescence of the supernatant was measured using the FITC filters with excitation and emission passband window at 485 and 530 nm, respectively. Before DoE analysis, the fluorescence of the CFDA-SE labeled samples was background-corrected by subtraction of the fluorescence from control samples and buffers.

#### H. pylori labeling

For CFDA-SE labeling of *H. pylori*, the strain J99 was plated on WC-dent plates, expanded once and harvested by scraping. All subsequent steps were performed as described for *E. coli*.

#### H. pylori PFA fixation

A PFA solution was freshly prepared by adding 1% PFA to PBS and heating to 70 °C in a water bath until dissolved. After cooling to room temperature, the solution was used to resuspend a bacterial pellet at room temperature adjusting the OD_600_ to 30 and incubated for 5 minutes. Fixed bacteria were washed twice with PBS before further treatment.

### Surface shaving with trypsin

The *H. pylori* strain J99 was grown on WC dent agar plates. Then, bacteria were harvested by scraping and split with regard to unfixed and PFA fixed conditions. Subsequently, bacteria were washed twice with PBS and resuspended in shaving buffer (1x PBS, 20% (w/v) sucrose, 10 mM DTT) to a final OD_600_ 30. 1 ml of the bacterial solution was distributed into 1.5 ml reaction tubes on ice. 10 µg sequencing grade Trypsin was added to one half, and the trypsin’s formulation buffer (50 mM acetic acid) added to the other half. Subsequently, bacteria were incubated at 37 °C under shaking in a Thermomixer (Eppendorf) at 500 rpm. After 10, 20 and 30 minutes, shaving was stopped by placing the corresponding tube on ice. Bacteria were pelleted by centrifugation and the supernatant filtered through a 0.22 µM syringe filter (sterilized by gamma irradiation, Millex^®^, Millipore). The supernatant was shock frozen in liquid nitrogen and stored at −20 °C until preparing the samples for MS analysis.

After each step viability was measured by counting CFU in triplicates and at the end morphology analyzed by microscopy. The experiment was performed independently in biological quadruplicates.

### Surface protein biotinylation

The biotinylation was performed with the Pierce™ Cell Surface Protein Isolation Kit according to manufacturer’s instructions and with the manufacturer’s proprietary buffers. Briefly, the *H. pylori* strain J99 was prepared unfixed and PFA fixed from the same batch of bacteria for the shaving experiment. After washing, bacteria were resuspended in PBS to a final OD_600_ of 30. The content of one vial Sulfo-NHS-SS-Biotin was dissolved in 48 ml ice-cold PBS. For biotinylation, 1 ml of bacterial suspension was added and incubated for 30 minutes at 4 °C under gentle agitation on an orbital rocking platform. Controls were treated similarly without the crosslinking reagent. To stop the reaction, 500 µl of the isolation kit’s quenching solution was added. Subsequently, cells were washed with TBS and lysed by addition of 750 µl of the isolation kit’s biotinylation lysis buffer with sonication for 4 × 1 minute on ice. Subsequently, the lysate was cleared by centrifugation and the clarified supernatant filtered through a 0.22 µM syringe filter (sterilized by gamma irradiation, Millex, Millipore). The supernatant was shock frozen in liquid nitrogen and stored at −20 °C. Afterwards, biotinylated proteins were affinity-enriched by NeutrAvidin Agarose Resin. The slurry was added to a centrifuge column assembly and washed three times with 500 µl of wash buffer by centrifugation for 1 minute at 1000 g. Then, the supernatant was added and incubated for 60 minutes at room temperature with end-over-end mixing. After removing the supernatant by centrifugation, the slurry was washed four times. Bound proteins were eluted by adding 400 µl the isolation kit’s elution buffer containing 50 mM DTT, incubating the suspension for 60 minutes at room temperature and collecting the supernatant by centrifugation. The samples were shock frozen in liquid nitrogen and stored at −20 °C until preparing the samples for MS analysis.

Viability and morphology were analyzed as described for surface shaving. The experiment was performed independently in biological quadruplicates alongside the surface shaving.

### Full proteome analysis

To analyze the *H. pylori* proteome, bacterial lysates were prepared with minor adaptations as described for cell lines (Hornburg *et al*. 2014). Briefly, an OD_600_ of 5 was taken from the harvested bacteria, washed twice in ice-cold PBS containing proteinase inhibitors and lysed in lysis buffer (4% SDS, 100 mM Tris, pH 8.0) with 4 × 1 minute sonication. Subsequently, the lysate was sterile filtered and protein concentration was approximated by measuring the absorption at 280 nm with 1 AU = 1 mg*ml^−1^.

### Sample preparation for mass spectrometry

100 µg protein sample was reduced with 10 mM DTT for 30 minutes and alkylated with 55 mM iodoacetamide for 45 minutes in an ultrasound water bath. To remove the detergent, proteins were precipitated. Therefore, 80% (v/v) acetone was added at −20 °C, incubated for 2 h and the resulting precipitate was pelleted by centrifugation and washed twice with 80% (v/v) acetone. Afterwards, the precipitated proteins are dissolved in 6 M Urea/2 M Thiourea, 10 mM Hepes, pH 8.0 and digested by addition of 1 µg LysC for 3 h. Subsequently, samples were diluted 1:5 in 50 mM sodium bicarbonate and digested by addition of 2 µg trypsin overnight. Finally, the resulting peptide mixtures are desalted by C18 StageTips and directly analyzed by MS.

### Mass spectrometry analysis with an Orbitrap mass spectrometer

Mass spectrometry analysis and data evaluation were carried utilizing a Thermo Scientific EASY nLC 1000 HPLC system directly coupled to an Orbitrap Elite™ quadrupole Orbitrap mass analyzer via a nano-electro spray source (Thermo Fischer Scientific) (Michalski *et al*. 2012), with the software Xcalibur acquiring the data. Peptides were loaded onto in-house packed columns (75 µM inner diameter, 20-cm length, 1.8 µM C18 particles) in MS Buffer A and separated for 70 min within a linear gradient from 5% MS buffer B to 60% MS buffer B at a flow rate of 250 nl/min with a column temperature set to 40 °C. The mass analyzer was operated in a data-dependent top15 mode with a survey scan range set to 300 to 1650 m/z and a resolution of 240,000 at 400 m/z. Selected peptides were subjected to collision induced dissociation with a normalized collision energy of 35. Repeated sequencing was limited by dynamically excluding sequences features for 30 seconds.

### Data analysis

We processed the raw data with MaxQuant^44^ (v. 1.5.3.14) and used the integrated search engine Andromeda^45^ to search MS/MS spectra against the *H. pylori* J99 UniprotKB Fasta database (1,488 forward entries; version from October 2015). The enzyme specificity was set to trypsin while allowing up to two miss cleavages and cleavage N-terminal to proline. We set the minimum length of peptides to be considered for identification to seven assuming carbamidomethyl of cysteines as fixed and methionine Oxidation (M) as well as acetylation of N-termini as variable modifications. A false discovery rate (FDR) cutoff of 1% was applied for both, the peptides and proteins.

We performed nonlinear retention time alignment of all measured samples in MaxQuant which allows us to transfer of peptide identifications in the absence of sequencing (MS1 only), within a maximum retention time window of 0.7 min (“Match between runs”). Protein intensities were normalized within MaxQuant (MaxLFQ,^21^) based on normalized extracted ion currents. We stringently filtered our data requiring at least two peptide ratios for protein quantification. In addition, common contaminants (n = 247) as well as proteins only identified with side modifications were strictly excluded from the analysis. Data analysis was performed within the PERSEUS framework^46^. Missing values were imputed based on distribution of all quantified proteins in the respective experiment (downshift 1.8, width 0.3).

**Quantified proteins (**Fig. 2A**):** The Maxquant protein groups intensity output was filtered for common contaminants, proteins only identified with side modification and reverse identifications. The bar denotes the median number of identified proteins for each group (quadruplicates), error bars show the standard deviation of the number of quantified proteins.

**Annotation coverage (**Fig. 2B**):** Annotations were matched the majority protein column for all quantified proteins (see quantified proteins). In addition, a gene list was created for all 1488 *H. pylori* proteins and similar and annotations (GOMF name, GOBP slim name, GOCC slim name, KEGG name, and Uniprot Keywords) were loaded for the Uniprot identifier in Perseus. All quantified proteins (see quantified proteins) were matched to this data frame according to the Uniprot identifier and percentage of coverage was calculated (see Supplementary Table coverage).

**Venn diagram (**Fig. 2C**):** Quantified proteins (see quantified proteins) were grouped accordingly requiring at least 3 valid values for a protein group to be counted.

**Annotation enrichment in Cluster 1 and 2 (**Fig. 2F**):** Enriched annotations for protein in Cluster 1 and Cluster 2 were calculated compared to all surfome samples (C10, C20, C30, T10, T20, T30, for both, PFA+ and PFA-) with a fisher exact test in Perseus. The results are plotted in an annotation volcano plot showing de-enriched and enriched annotations (see supplementary tab cluster C1 C2 enrichment).

**PCA:** The orthogonal component 1 and 2 differentiated between these two effects. For identifying groups of protein profiles that are release by secretion and proteins on the bacterial surface, we employed an unsupervised K-means hierarchical clustering (300 clusters) on ANOVA significant (S0: 1, FDR: 5%) profiles. Absolute abundances in the entire total proteome were calculated based on the size normalized protein intensities (iBAQ, (Schwanhäusser *et al*. 2011)) and plotted against their respective rank. Density distributions were calculated with R^47^ and scaled to similar intensities in order to improve comparison of distribution shifts along the dynamic range of the total proteome.

**Biotinylation**: We first assessed similarity of the samples in an unsupervised hierarchical k-means clustering. For both groups, biotinylation and control, we identified one sample of the quadruplicate to exhibit a very different protein pattern and removed these for further analysis. We next performed a Welch’s t- test and applied a 5% permutation based false discovery cut-off with an S0 correction of 1 on proteins that were detected with at least 2 valid values in at least one group. To benchmark the biotinylation approach against our trypsinization strategy we performed a 2D annotation enrichment on the T-test difference of biotinylation vs biotinylation control and trypsin treatment (+trypsin, 10 min, PFA positive) vs control (−trypsin, 10 min, PFA positive). Supplementary Table biotinylation 2D and Supplementary Fig. 2C shows all categorical annotations with a p-value <0.05.

### Candidate selection

#### Correlation analysis

To identify surface associated proteins from the 1153 quantified proteins of *H. pylori*, individual experiments were first numerically annotated according to the trypsin treatment and incubation time (10, 20 or 30). Since for control samples (no trypsin treatment) no surface shaving is expected irrespective of the time point, these samples were numerically annotated with 0. Missing values were imputed and replicates were aggregated to the median. Next, we performed a Spearman rank correlation analysis employing a permutation based FDR of 5% (250 randomizations) of protein abundance profiles to the numerical annotation (0, 10, 20 or 30). 146 proteins that significantly correlated with a coefficient of greater than 0.75 were kept. The replicates for these 146 profiles were averaged (median) and z-scored. We next employed an unsupervised k-means clustering to further filter the data for expected surface shaving profiles. We identified two clusters (c1, c2) containing 54 and 19 proteins (73 in sum), respectively.

#### Homology analysis

The first Uniprot identifier of the protein groups in c1 and c2 were extracted. With this Uniprot identifiers a blast search (“blastp”, non-redundant database, hit list size 2000, e-value threshold 10) was performed interrogating NCBI via a BioPython http://www.biopython.org/ (Bio.Blast, NCBIWWW)^48,49^. Uniprot identifier was searched (a) against all bacteria (taxonomy ID 2) excluding *H. pylori* (taxonomy ID 210), (b) Homo sapiens (taxonomy ID 9606) and (c) *H. pylori* (taxonomy ID 210). The search against *H. pylori* returned 72 of the submitted 73 identifiers missing Q9ZLC5.

The identity was calculated by dividing the hsp.identities by alignment.length. Filter: (a) compared to other bacterial proteins, identities >33% were excluded (excluding 37 candidates). (b) When compared to human proteins, identities >5% and e-value >1 −log10 were excluded (excluding 46 candidates). Both filters were applied reducing our candidate list to 22. To visualize the identity within *H. pylori* we extracted the best identity scores of remaining candidates compared to the proteomes of 264 *H. pylori* strains.

### Data resources

The mass spectrometry proteomics data have been deposited to the ProteomeXchange Consortium via the PRIDE^49,50^ partner repository with the dataset identifier PXD012566 (http://proteomecentral.proteomexchange.org).

## Supplementary information

Supplementary information
