# Supplementary material for: A mass spectrometry guided approach for the identification of novel vaccine candidates in gram-negative pathogens
Source: Sci Rep. 2019 Nov 22;9:17401. doi: 10.1038/s41598-019-53493-8 (PMC6874673; doi:10.1038/s41598-019-53493-8)
Supplement: Supplementary file 1 — Supplementary information [file 41598_2019_53493_MOESM1_ESM.pdf]

A mass spectrometry guided approach for the identification of novel vaccine candidates in gram-negative pathogens

Daniel Hornburg[2,5‡], Tobias Kruse[1,3‡], Florian Anderl[1,3], Christina Daschkin[1], Raphaela P. Semper[1,6], Kathrin Klar[3], Anna Guenther[4], Raquel Mejías-Luque[1,6], Nicole Schneiderhan-Marra[4], Matthias Mann[2], Felix Meissner[2‡*], Markus Gerhard[1,3,6‡*]

Affiliations:
[1] Institut für Medizinische Mikrobiologie, Immunologie und Hygiene, Technische Universität München; Munich, Germany.
[2] Max-Planck-Institute for Biochemistry, Martinsried, Germany
[3] ImevaX GmbH, Munich, Germany
[4] Naturwissenschaftliches und Medizinisches Institut, Tübingen, Germany
[5] present address: Stanford University, School of Medicine, USA
[6] German Center for infection research, partner site Munich

[‡] These authors contributed equally to this work
*Correspondence to: markus.gerhard@tum.de, meissner@biochem.mpg.de

Supplementary Information

Supplementary Figure 1

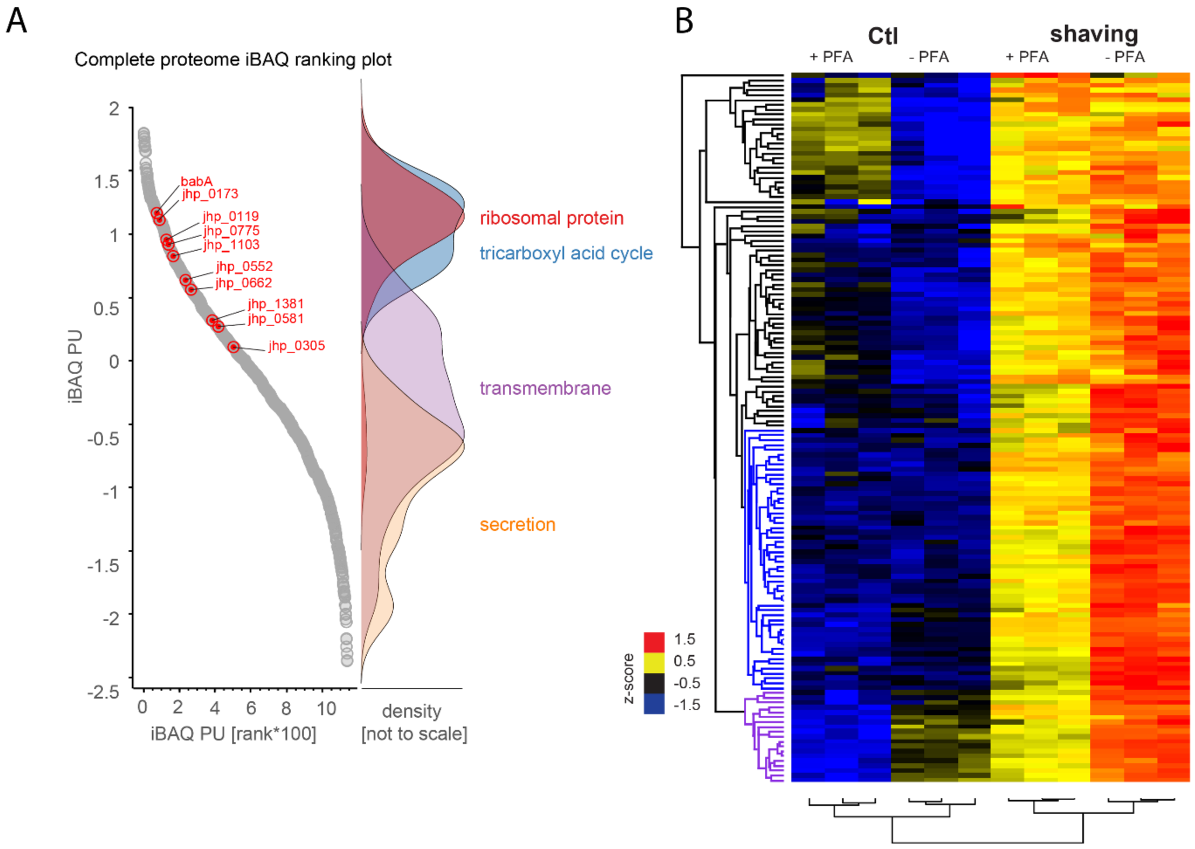

Supplementary Figure 1 | **Abundance profile of vaccine candidates in the entire *H. pylori* proteome** **A) Complete Proteome Analysis:** To visualize the composition of the total *H. pylori* (J99) (median of unfixed total proteome samples, median of at least 2 valid values) proteome, log10 protein abundances (size normalized intensities: iBAQ (iBAQ (Schwanhausser et al., 2011))) are plotted against their abundance rank. Quantified proteins span more than 4 orders of magnitude. Candidate proteins, known survival and adhesion factors are indicated in red. The density distribution (scale normalized) shows the distribution of proteins belonging to housekeeping molecular processes (ribosomal proteins and tricarboxylic acid cycle, red and blue) as well as transmembrane (pink) and secretion (yellow). **B) Surfome of *H. pylori*:** 146 proteins that correlate with the trypsin shaving time course. Median of replicates was z-scored and plotted after performing K-means hierarchical clustering.

Supplementary Figure 2

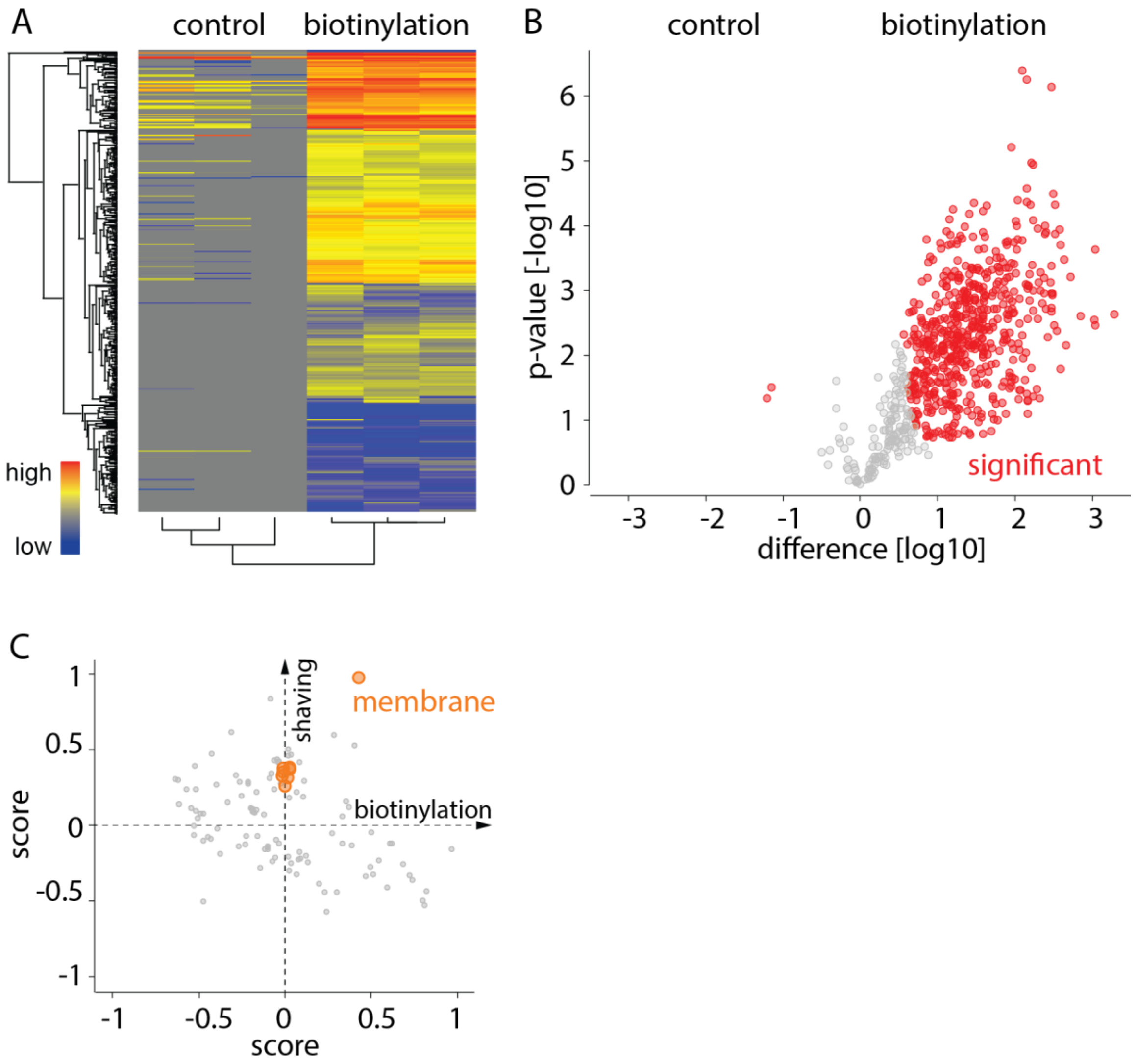

Supplementary Figure 2 |**Mapping of surface associated proteins via biotinylation.**

A) Heat map indicating 712 proteins identified by using biotinylation and biotin enrichment to map the surface of *H. pylori*. **B)** T-test comparison visualized as a volcano plot showing differential abundance of proteins in the biotinylation control compared to the biotinylation pulldown, 565 proteins were significantly enriched (Welch's t-test, FDR 5%, S0=1). **C)** Comparison of surfome shaving and biotinylation for enriched annotations: 2D enrichment of categorical annotations (see supplementary table biotinylation 2D) based on the t-test difference comparing biotinylation (x axis, biotinylation vs control) and one surfome shaving condition (trypsin treatment (PFA) after 10 min vs control (PFA) after 10 min). Positive scores denote enrichments (p-value < 0.05) and orange circles indicate membrane annotations.

Supplementary Figure 3

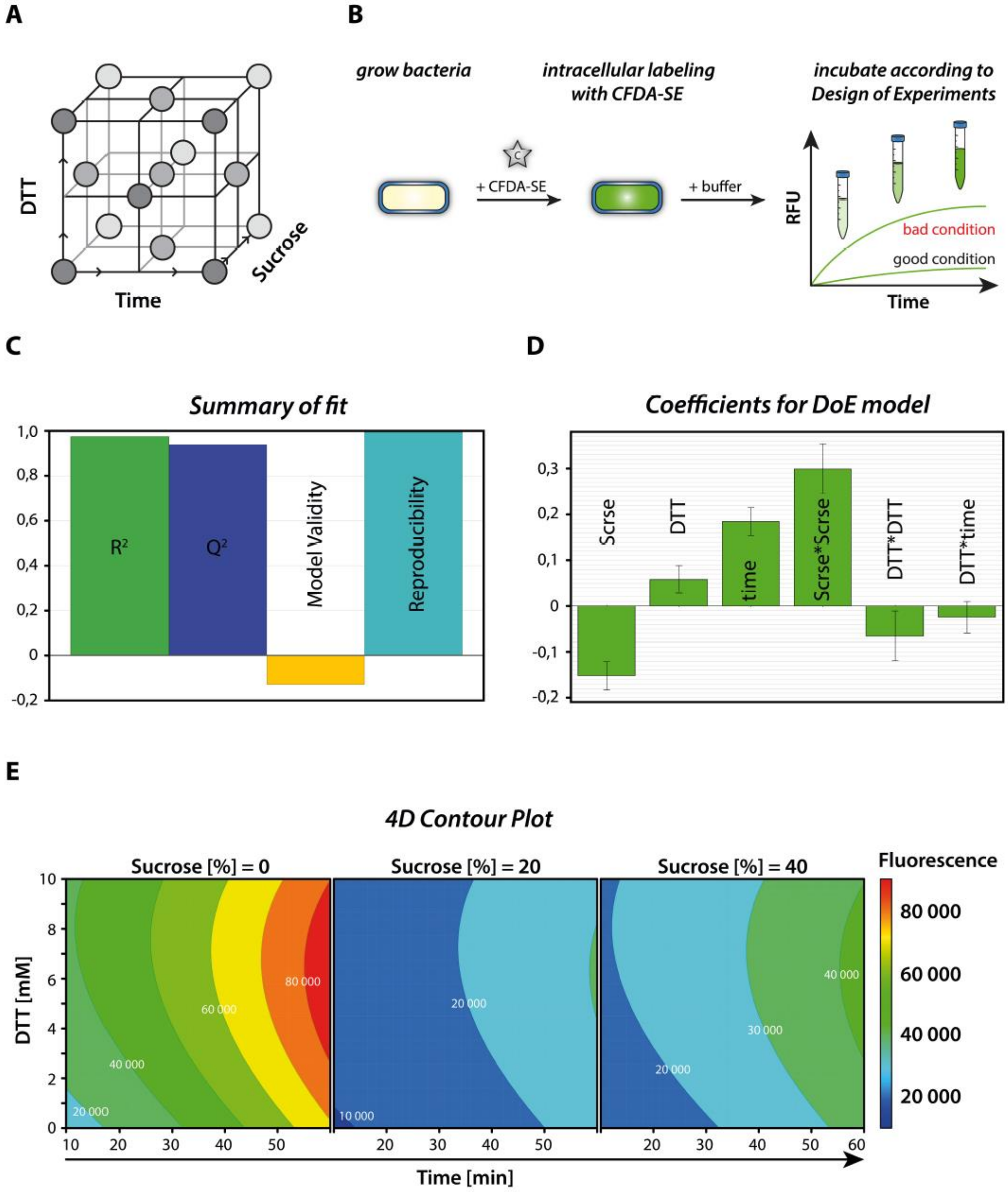

Supplementary Figure 3 | **Design of Experiment (DoE) to optimize the shaving conditions for gram-negative bacteria.**

To optimize the shaving buffer composition, fluorescently labeled *E. coli* were incubated in different buffer conditions according to a design of experiments (DoE) approach. Then, the fluorescence of the supernatant was measured and evaluated by the DoE software MODDE (Umetrics). The DoE objective was optimization with the recommended design (CCF, star distance = 1), running 19 experiments with 5 center points. Quadratic Modeling was used to quantify linear, pairwise and quadratic interactions of the variables. **A)** Concentrations of DTT, time and sucrose were varied, covering the entire experimental space in high and low combinations. **B)** Bacteria were labeled with CFDA-SE, and thus, proteins in the cytoplasm labeled fluorescently. Upon incubation, autolysis is measured by an increase of fluorescence intensity in the supernatant. **C)** The DoE data were fitted and evaluated, showing the quality parameters Q^2 (green), R^2 (blue), model validity (yellow) and

reproducibility (turquoise). **D)** After removing non-significant terms, the fitting equation is only comprised of variables influencing the readout significantly. Linear terms are represented by single expressions (e.g. DTT), pairwise interactions by multiplication of two separate terms (e.g. DTT*t) and quadratic interactions by multiplication of the same term (e.g. scrse*scrse). **E)** A 4-D contour plot visualization of the fitted fluorescence data after processing the DoE model.

Supplementary Figure 4

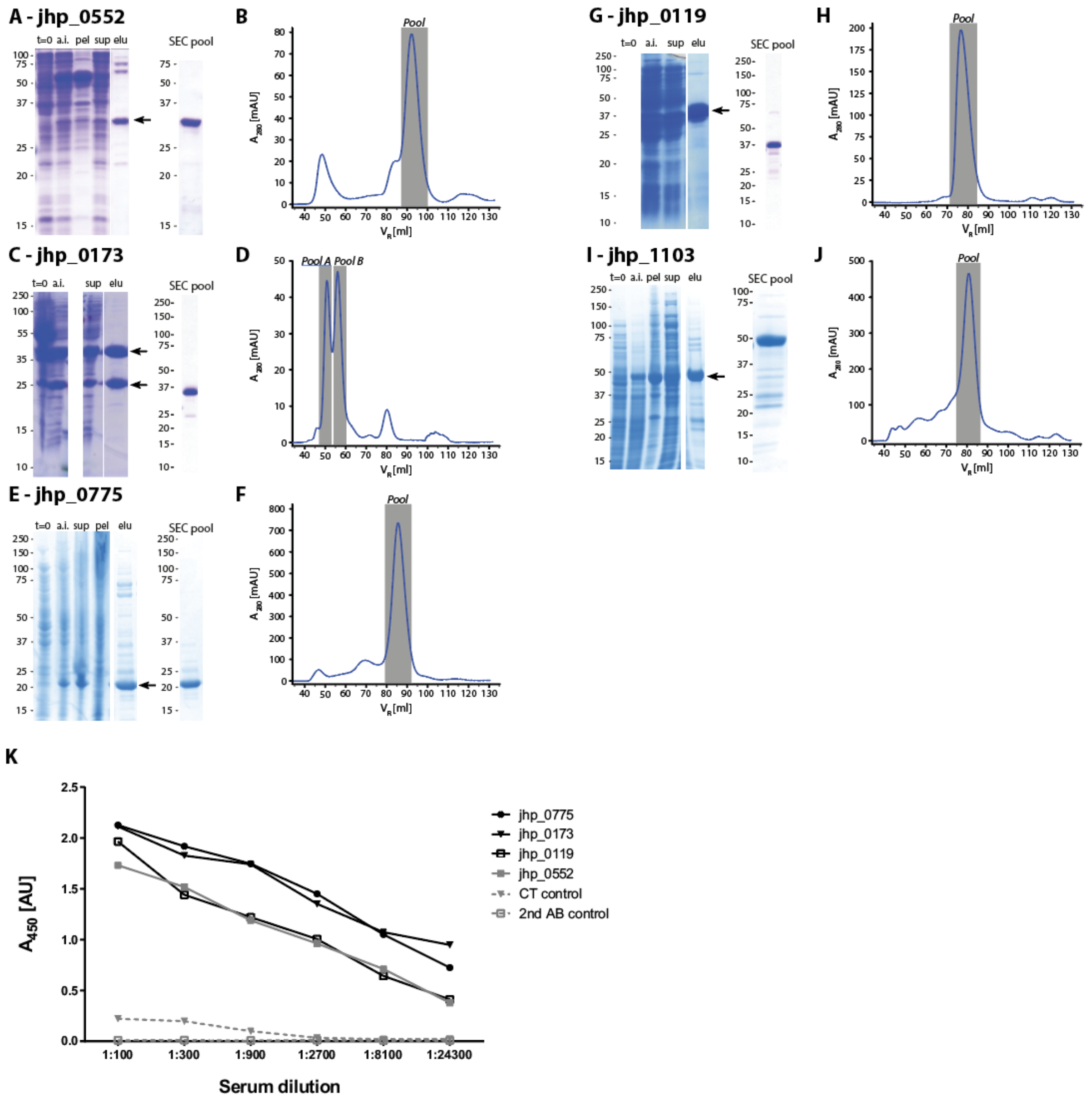

Supplementary Figure 4 | **Protein production and generation of protein specific antisera.** Proteins were recombinantly produced in *E. coli* BL21(DE3) and purified by NiNTA and size exclusion chromatography (SEC). SDS page analysis and SEC elution profiles are depicted for jhp_0552 (**A, B**), jhp_0173 (**C, D**), jhp_0775 (**E, F**), jhp_0119 (**G, H**) as well as jhp_1103 (**I, J**). SEC pooled fractions are highlighted in the chromatogram (grey) and the final pool analyzed by SDS-PAGE.

K) ELISA using antisera raised against the vaccine candidates. The IgG fraction was purified and used to stain the surface of CFDA-SE labeled *H. pylori*. The reactivity of the anti-vaccine candidate immune sera against their corresponding antigen is shown.

Supplementary Figure 5

A

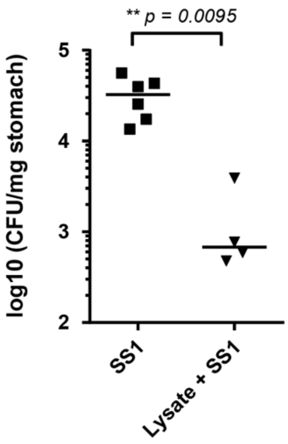

Supplementary Figure 5| **Immunization of *H. pylori* infected mice with *H. pylori* lysate.**

A) Mice were infected with *H. pylori* SS1 and immunized mice with cholera toxin (CT) alone or in combination with *H. pylori* lysate. Gastric *H. pylori* colonization was determined and depicted as CFU per mg stomach.

Supplementary Figure 6

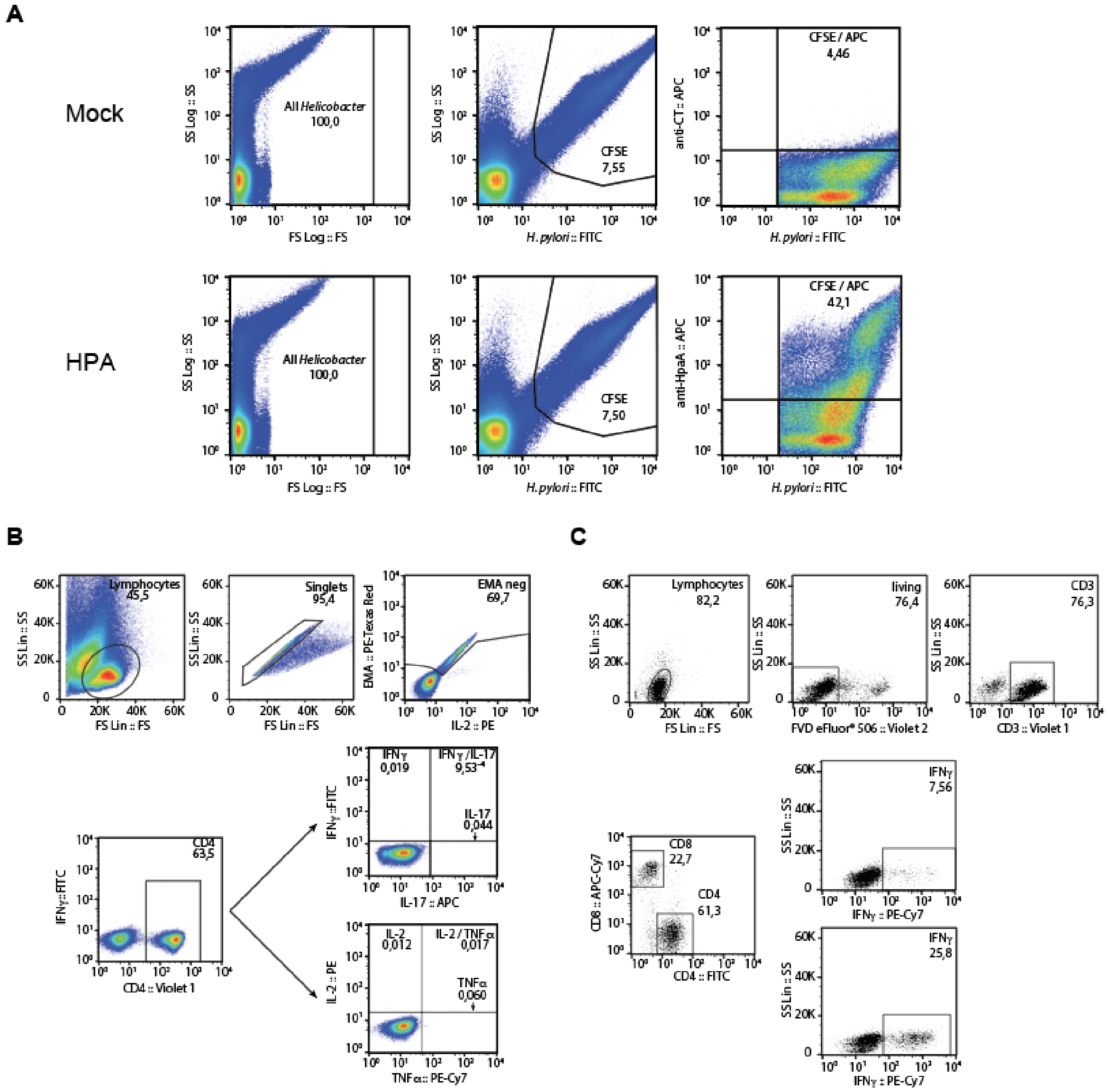

Supplementary Figure 6| **Gating strategies for flow cytometry analysis.**

A) Representative density plots of the gating strategy to detect CFDA-SE labeled *H. pylori* stained with antisera raised against vaccine candidates. Staining with antibodies derived from mock immunized (CT control) and HPA immunized (HPA) mice are shown. **B)** Mouse splenocytes were stimulated with jhp_0775, stained and analyzed by flow cytometry. Representative density plots from the ICS gating strategy are shown, gating on living single lymphocytes (upper panel) and measuring the frequencies of IFNγ and IL-17 (middle-right plot) as well as IL-2 and TNFα (lower-right plot) producing $CD4^+$ T cells (lower-left plot). **C)** Human PBMCs were stimulated with PMA/Ionomycin, stained and analyzed by flow cytometry. Representative dot plots from the ICS gating strategy are shown, gating on $CD3^+$ living lymphocytes (upper panel) and measuring the frequencies of IFNγ producing $CD8^+$ (middle-right plot) and $CD4^+$ cells (lower-right plot).

suppl HP homology of 22 final candidates

Gene name	HP 26695	HP J99	HP G27	HP P12	HP B38	HP 908	HP SJM180	HP PeCan4	HP India7
jhp_1276	0,97619	1	0,97619	0,964286	0,97619	0,97619	0,9761904	0,9401197	0,97619
jhp_0776	0,965854	1	0,965854	0,97561	0,965854	0,990244	0,9853659	0,9658536	0,965854
jhp_0718	0,923077	1	0,925926	0,927739	0,955711	0,979021	0,9370629	0,9324009	0,916667
jhp_0552	0,983229	1	0,964361	0,987421	0,981132	0,951782	0,932914	0,932914	0,960168
jhp_0530	0,900356	1	0,865772	0,836178	0,87372	0,992883	0,9726962	0,9209622	0,933333
jhp_0501	0,866044	1	0,900312	0,931464	0,890966	0	0,9320987	0,8425926	0
cagN	0,95098	1	0,95098	0,941177	0	0,960784	0,9869281	0,9339934	0,947712
orf17	0,992064	1	0,996032	0,984127	0	0,984127	0,9960318	0,9920635	0,984127
orf8	0,939709	1	0,933472	0,931393	0	0,968815	0,9355509	0,9480249	0,929019
orf6	0,956522	1	0,956522	0,947826	0	0,982609	0,9478261	0,9652174	0,965217
jhp_0444	0,917266	1	0,902878	0,884892	0,902878	0,953901	0,8127208	0,822695	0,899281
jhp_0369	0,926056	1	0,911439	0,929825	0,915493	0,982456	0,9333333	0,90625	0,929825
jhp_0173	0,876405	1	0,895911	0,914498	0,883895	0,977695	0,8884758	0,8689138	0,88015
jhp_0136	0,941177	1	0,941177	0,897059	0,911765	0,970588	0,9558824	0,9411765	0,941177
lpp20	1	1	0,994286	0,994286	0,988571	0,994286	0,9942857	0,9942857	0,977143
jhp_1355	0,97093	1	0,974359	0,974359	0,974359	1	0,974359	0,974359	0,964103
babB	0,891243	1	0,894817	0,922078	0,655496	0	0,898017	0,8799415	0,89548
jhp_1103	0,881435	1	0,892356	0,866044	0,712698	0,879418	0,8796875	0,8367029	0,73817
babA	0,93588	1	0,922043	0,922043	0,899464	0,812766	0,850067	0,8230563	0,895722
jhp_0775	0,985075	1	0,97537	0,9801	0,985222	0,995025	0,9950249	1	0,99005
Cag_14	0,562014	1	0,224324	0,8606	0,242021	0,978836	0,9600219	0,8387097	0,618533
jhp_0119	0,940559	1	0,923077	0,933798	0,947552	0,988142	0,9754386	0,916955	0,891608

suppl HP homology of 22 final candidates

Gene name	HP Lithuania75	HP Gambia94/24	HP 2018	HP 2017	HP ELS37	HP HUP-B14	HP NQ4200
jhp_1276	0,9761904	0,9821429	0,97619	0,97619	0,97619	0,9821429	0,9760479
jhp_0776	0,9658536	0,9804878	0,990244	0,990244	0,980488	0,9658536	0,9756098
jhp_0718	0,9300699	0,9627039	0,979021	0,979021	0,93911	0,9370629	0,9370629
jhp_0552	0,9559748	0,951782	0,951782	0,951782	0,991614	0,9832285	0,9827957
jhp_0530	0,9146758	0,9583333	0,992883	0,992883	0,921708	0,8958333	0,9010239
jhp_0501	0,8796296	0,9688473	0,941358	0,941358	0	0,904321	0,9259259
cagN	0,9477124	0,9738562	0,957516	0,957516	0,96732	0,9509804	0,9705882
orf17	0,984127	0,9801587	0,984127	0,984127	0,992064	0,9801587	0,9960318
orf8	0,929314	0,972973	0,970894	0,970894	0,933472	0,9313929	0,968815
orf6	0,9565217	0,9826087	0,982609	0,982609	0,973913	0,973913	0,9558824
jhp_0444	0,9028777	0,9539007	0,957447	0,957447	0,910394	0,9064748	0,9107143
jhp_0369	0,9357143	0,9964913	0,982456	0,982456	0,905263	0,9578947	0,9473684
jhp_0173	0,8981132	0,9739777	0,977695	0,977695	0,973283	0,9213483	0,8838952
jhp_0136	0,9264706	0,9705882	0,970588	0,970588	0,941177	0,9411765	0,9558824
lpp20	0,9885714	0,9885714	0,994286	0,994286	0,994286	0,9942857	0,9885714
jhp_1355	0,9538462	0,9846154	1	1	0,887755	0,9794872	0,9709302
babB	0,8895801	0,9458272	0	0,636232	0,898449	0,6798906	0,9164306
jhp_1103	0,8705148	0,7523659	0,877517	0,881956	0,884555	0,8685898	0,8439938
babA	0,922043	0,8847185	0,792453	0,571015	0,854817	0,9097127	0,8788282
jhp_0775	0,9900498	0,9950249	0,995025	0,995025	0,9801	0,9800995	0,9950249
Cag_14	0,5653306	0,8844169	0,996248	0,996248	0,628233	0,2275132	0,6765714
jhp_0119	0,9333333	0,9894737	0,988142	0,988142	0,965035	0,9719298	0,8986784

suppl HP homology of 22 final candidates

Gene name	HP NQ4099	HP NQ4228	HP NQ4044	HP NQ407(	HP NQ4053	HP NQ4161	HP NQ4110	HP Hp A-4
jhp_1276	0,9821429	0,9821429	0,9761904	0,982143	0,9479769	0,9821429	0,9821429	0,982143
jhp_0776	0,9902439	0,9658536	0,9707317	0,985366	0,9609756	0,9902439	0,9707317	1
jhp_0718	0,9277389	0,9557109	0,9627039	0,937355	0,9370629	0,948718	0,9440559	0,974359
jhp_0552	0,9392034	0,9643606	0,9832285	0,981132	0,9643606	0,9559748	0,9827957	0,953192
jhp_0530	0,9090909	0,9385666	0,9572954	0,912587	0,8771331	0,9679715	0,9252669	0,901024
jhp_0501	0,9074074	0,941358	0,8981481	0,91358	0,8981481	0,8981481	0,8878505	0,978193
cagN	0,9803922	0,9738562	0,9542484	0,980392	0	0	0	0,960656
orf17	0,9801587	0,984127	0,984127	0,992064	0	0	0	0,984127
orf8	0,9272349	0,9397089	0,9209979	0,933472	0	0	0	0,978723
orf6	0,9304348	0,9558824	0,9652174	0,930435	0	0	0	0,955882
jhp_0444	0,9139785	0,8956835	0,9045936	0,902878	0,8971631	0,9028777	0,9172662	0,573477
jhp_0369	0,9754386	0,9719298	0,9013606	0,975439	0,9298246	0,9754386	0,9214286	0,978947
jhp_0173	0,8951311	0,9033457	0,8949276	0,906367	0,8876405	0,9291045	0,8777778	0,977695
jhp_0136	0,9264706	0,9264706	0,9411765	0,955882	0,9264706	0,9117647	0,9411765	1
lpp20	0,9942857	0,9885714	0,9885714	0,988571	0,9942857	0,9942857	0,9942857	0,994286
jhp_1355	0,9641026	0,9883721	0,9794872	0,969231	0,9641026	0,9641026	0,9794872	0,989744
babB	0,6410999	0,9004393	0,9497817	0,905099	0,8942172	0,9022663	0,909732	0,918009
jhp_1103	0,8361934	0,8984127	0,8829953	0,886115	0,8875	0,8861154	0,7281399	0,879875
babA	0,8514056	0,8236131	0,8511502	0,866936	0,9086022	0,877027	0,7263752	0,887989
jhp_0775	0,9950249	1	0,9800995	0,975124	0,9800995	0,9950249	0,9850746	1
Cag_14	0,614552	0,6716332	0,9216	0,574392	0,2393617	0,2367021	0,2367021	0,634173
jhp_0119	0,951049	0,9531915	0,9230769	0,885106	0,9615384	0,9719298	0,9230769	0,987124

suppl HP homology of 22 final candidates

Gene name	HP Hp A-9	HP Hp A-5	HP Hp A-20	HP Hp A-17	HP Hp H-28	HP Hp H-24	HP Hp H-27	HP Hp H-29
jhp_1276	0,975	0,9761904	0,9702381	0,9761904	0,9761904	0,9761904	0,9642857	0,9821429
jhp_0776	0,9609756	0,9902439	0,9902439	0,9804878	0,9609756	0,9804878	0,9804878	0,9853659
jhp_0718	0,9254079	0,951049	0,974359	0,9533799	0,934732	0,974359	0,9300699	0,965035
jhp_0552	0,9811321	0,9638298	0,9622642	0,9685535	0,9361702	0,9531915	0,9622642	0,9787234
jhp_0530	0,8187919	0,9572954	0,9644128	0,9715303	0,9032258	0,9893239	0,9180887	0,9964413
jhp_0501	0,8981481	0,9813084	0,9719626	0,9750779	0,9003115	0,9719626	0,8487654	0,9750779
cagN	0,9575163	0,9640523	0,9607843	0	0,9771242	0,9705882	0	0,9738562
orf17	0,984127	0,984127	0,9801587	0,984127	0,9835391	0,984127	0	0,984127
orf8	0,9230769	0,97921	0,972973	0,972973	0,9272349	0,981289	0	0,970894
orf6	0,9565217	0,9826087	1	0,9705882	0,9478261	0,98	0	0,9826087
jhp_0444	0,913669	0,9539007	0,9503546	0,9052632	0,9208633	0,9680851	0,9064748	0,964539
jhp_0369	0,916955	0,9894737	0,9723184	0,9859649	0,9119718	0,9859649	0,9157895	0,9789473
jhp_0173	0,9144982	0,9739777	0,9851301	0,9739777	0,8764045	0,9739777	0,8801498	0,9851301
jhp_0136	0,9411765	0,9705882	0,9705882	0,9411765	0,9411765	0,9705882	0,9411765	0,9705882
lpp20	0,9942857	0,9942857	0,9942857	0,9942857	0,9942857	0,9942857	0,9942857	0,9885714
jhp_1355	0,9709302	0,9794872	0,9897436	0,9825581	0,9794872	0,994186	0,9794872	0,9883721
babB	0,9065156	0,9320113	0,8571429	0,6583679	0,9163059	0,9171348	0,9148629	0,9305949
jhp_1103	0,8858521	0,9402516	0,9107981	0,879257	0,8501872	0,9015625	0,7199367	0,9296875
babA	0,9193549	0,8929539	0,9203779	0,8976487	0,9006897	0,8927614	0,7056277	0,8921739
jhp_0775	0,9850746	0,9950249	0,9950249	1	0,9900498	0,9950249	0,9900498	0,9900498
Cag_14	0,5601249	0,6658522	0,9688184	0,9887429	0,8595642	1	0,2195767	0,6118996
jhp_0119	0,9358974	0,9871244	0,9871244	0,9742489	0,9427313	0,958042	0,9103943	0,9828326

suppl HP homology of 22 final candidates

Gene name	HP Hp H-30	HP Hp H-36	HP Hp H-41	HP Hp H-42	HP Hp H-43	HP Hp H-44	HP Hp H-45	HP Hp A-6
jhp_1276	0,9761904	0,9761904	0,9821429	0,9821429	0,9761904	0,9821429	0,9702381	0,9702381
jhp_0776	0,9804878	0,9902439	0,9902439	0,9853659	0,9756098	0,9902439	0,9756098	0,9804878
jhp_0718	0,969697	0,969697	0,9557109	0,97669	0,9440559	0,97669	0,9300699	0,97669
jhp_0552	0,9538784	0,9622642	0,9538784	0,9643606	0,9832285	0,9595745	0,9874214	0,9580713
jhp_0530	0,9537367	0,9644128	0,9893239	0,9846154	0,8327645	1	0,9288256	0,9893239
jhp_0501	0,9719626	0,9657321	0,9813084	0,9688473	0,8598131	0,9688473	0,9190031	0,9537037
cagN	0,9673203	0,9673203	0,9640523	0,9607843	0	0,9705882	0,9575163	0,9771242
orf17	0,9801587	0,984127	0,984127	0,984127	0	0,984127	0,9920635	0,984127
orf8	0,981289	0,981289	0,9771309	0,983368	0	0,9771309	0,9313929	0,983368
orf6	0,973913	0,9913043	0,9913043	0,9913043	0	0,96	0,9565217	0,9826087
jhp_0444	0,5770609	0,5770609	0,9574468	0,9716312	0,913669	0,7928572	0,9011407	0,7472924
jhp_0369	0,9824561	0,9824561	0,9929824	0,9894737	0,9157895	0,968421	0,9157895	0,9824561
jhp_0173	0,9776952	0,9739777	0,936803	1	0,8951311	0,9776952	0,8959108	0,9776952
jhp_0136	0,9705882	0,9705882	0,9411765	0,9705882	0,9117647	0,9705882	0,9411765	0,9411765
lpp20	0,9942857	0,9942857	0,9885714	0,9942857	0,9942857	0,9942857	0,9942857	0,9942857
jhp_1355	0,9897436	0,9846154	0,9897436	0,9825581	0,9948718	0,9897436	0,9846154	0,9794872
babB	0,9138418	0,7410586	0,9138418	0,9191919	0,9191919	0,9458272	0,6518219	0,9458272
jhp_1103	0,93125	0,9110764	0,9141966	0,9282371	0,8829953	0,921875	0,8798077	0,900156
babA	0,9055331	0,8665768	0,8953488	0,9157609	0,7085137	0,8636977	0,9122807	0,9341463
jhp_0775	0,9950249	0,9950249	0,9900498	0,9950249	0,9900498	0,9950249	0,9751244	0,9950249
Cag_14	0,9716312	0,6514393	0,6693002	0,9679204	0,2248677	0,6426814	0,6176471	0,9842271
jhp_0119	0,9656652	0,9871244	0,9785408	0,9871244	0,9405594	0,9871244	0,9234043	0,9828326

suppl HP homology of 22 final candidates

Gene name	HP Hp A-8	HP Hp A-16	HP Hp A-26	HP Hp H-4	HP Hp H-3	HP Hp H-6	HP Hp H-9	HP Hp H-10
jhp_1276	0,9702381	0,9761904	0,9702381	0,9821429	0,9761904	0,9821429	0,9700599	0,9761904
jhp_0776	0,9804878	0,9902439	0,9707317	0,9902439	0,9853659	0,9948186	0,9609756	0,9902439
jhp_0718	0,981352	0,972028	0,9370629	0,9606481	0,9533799	0,9533799	0,9440559	0,9557109
jhp_0552	0,9580713	0,9475891	0,9496855	0,9559748	0,9832285	0,9538784	0,9832285	0,9553192
jhp_0530	0,9893239	0,9893239	0,9146758	0,9537367	0,9822064	0,9644128	0,8703071	0,9608541
jhp_0501	0,9688473	0,9719626	0,9844237	0,9719626	0,9657321	0,94081	0,8734568	0,9501557
cagN	0,9607843	0,9738562	0	0,8477011	0,9640523	0,9673203	0,9639344	0,9673203
orf17	0,984127	0,9880952	0	0,984127	0,9960318	0,984127	0,984127	0,9871795
orf8	0,9771309	0,95842	0	0,97921	0,972973	0,9787234	0,9334719	0,9771309
orf6	0,9913043	0,9565217	0	0,973913	0,9913043	0,9852941	0,9565217	0,9826087
jhp_0444	0,9539007	0,9574468	0,913669	0,8982456	0,9609929	0,609319	0,9244604	0,9574468
jhp_0369	0,968421	0,9480969	0,9031142	0,9824561	0,9824561	0,9614035	0,899654	0,9824561
jhp_0173	0,9776952	0,8764045	0,8913858	1	0,9702602	0,8838952	0,9033457	0,9296296
jhp_0136	0,9705882	0,9411765	0,9411765	0,9558824	0,9705882	0,9705882	0,9264706	0,9411765
lpp20	0,9942857	0,9885714	0,9942857	0,9942857	0,9942857	0,9942857	0,9942857	0,9942857
jhp_1355	0,9846154	0,9948718	0,9794872	0,9794872	0,9897436	0,9846154	0,9794872	0,9897436
babB	0,6783708	0,6531714	0,8873457	0,9194729	0,9225807	0,6671177	0,6487214	0,671141
jhp_1103	0,9254658	0,909375	0,7199367	0,925	0,9294671	0,8814353	0,8861154	0,926677
babA	0,866573	0,8987854	0,691358	0,9183673	0,9260753	0,8794326	0,9138627	0,8456376
jhp_0775	0,9950249	0,9950249	0,9900498	0,9950249	1	0,9950249	0,9800995	0,9950249
Cag_14	0,6706731	0,919418	0,2195767	0,9774105	0,6344606	0,8956522	0,6148909	0,9924242
jhp_0119	0,9828326	0,9871244	0,9198312	0,9742489	0,9871244	0,9828326	0,9300699	0,9333333

suppl HP homology of 22 final candidates

Gene name	HP Hp H-18	HP Hp H-11	HP Hp H-21	HP Hp H-19	HP Hp H-23	HP Hp H-34	HP Hp P-2	HP Hp P-1
jhp_1276	0,9821429	0,9702381	0,9821429	0,9761904	0,9761904	0,9761904	0,9702381	0,9821429
jhp_0776	0,9902439	0,9609756	0,9902439	0,9853659	0,9853659	0,9902439	1	0,9756098
jhp_0718	0,972028	0,9277389	0,969697	0,9440559	0,962877	0,979021	0,974359	0,967366
jhp_0552	0,951782	0,9874214	0,9475891	0,9680851	0,9538784	0,9481328	0,966457	0,9496855
jhp_0530	0,9608541	0,8926175	0,9295302	0,9078498	0,9537367	0,9537367	0,9044369	0,9644128
jhp_0501	0,9719626	0,8919753	0,9688473	0,9844237	0,9657321	0,9563863	0,9719626	0,9688473
cagN	0,9705882	0,9607843	0	0,9575163	0,9705882	0	0,9640523	0,9640523
orf17	0,984127	0,9880952	0	0,984127	0,9801587	0	0,9920635	0,984127
orf8	0,985447	0,9251559	0	0,97921	0,972973	0	0,9771309	0,985447
orf6	0,9913043	0,9652174	0	0,9826087	0,9913043	0	0,9913043	0,94
jhp_0444	0,9539007	0,9172662	0,9609929	0,9503546	0,9609929	0,8098592	0,9535714	0,9574468
jhp_0369	0,9859649	0,9428571	0,9789473	0,9789473	0,968421	0,9614035	0,9894737	0,9614035
jhp_0173	0,9814126	0,8764045	0,9888476	0,9851301	0,9814126	0,9814126	0,9814126	0,9814126
jhp_0136	0,9558824	0,9411765	0,9705882	0,9411765	0,9705882	0,9705882	0,9705882	0,9705882
lpp20	0,9942857	0,9885714	0,9942857	0,9942857	1	1	0,9942857	0,9885714
jhp_1355	0,9794872	0,9692308	0,974359	1	0,9794872	1	0,9846154	0,9948718
babB	0,9083216	0,9018759	0,7616959	0,8655462	0,9110169	0,7618365	0,9341142	0,6565517
jhp_1103	0,878125	0,8982785	0,9203125	0,9068323	0,925	0,9297972	0,8923557	0,8907956
babA	0,8872283	0,9026128	0,847561	0,8739837	0,8924731	0,8936743	0,8857143	0,8593103
jhp_0775	0,9950249	0,9900498	0,9950249	0,9950249	1	0,9950249	1	0,9950249
Cag_14	0,6444308	0,6057563	0,2393617	0,6344561	0,9962476	0,2222222	0,9719251	0,5982783
jhp_0119	0,9785408	0,9333333	0,9871244	0,9785408	0,9785408	0,9828326	0,9871244	0,9871244

suppl HP homology of 22 final candidates

Gene name	HP Hp P-3	HP Hp P-8	HP Hp P-4	HP Hp P-11	HP Hp P-25	HP Hp H-24b	HP Hp H-24c	HP Hp P-1b
jhp_1276	0,9821429	0,9761904	0,9821429	0,9880952	0,9821429	0,9761904	0,9761904	0,9821429
jhp_0776	1	0,9804878	1	0,9902439	0,9804878	0,9804878	0,9804878	0,9756098
jhp_0718	0,969697	0,986014	0,951049	0,9463869	0,9264368	0,974359	0,974359	0,967366
jhp_0552	0,9574468	0,9516807	0,9811321	0,9433962	0,9601677	0,9531915	0,9531915	0,9510638
jhp_0530	0,9679715	0,9861111	0,9078498	0,9692833	0,9777778	0,9893239	0,9893239	0,9658703
jhp_0501	0,9688473	0,9750779	0,9595016	0,9351852	0,9795918	0,9719626	0,9719626	0,9688473
cagN	0,9542484	0	0,9738562	0,9705882	0,9738562	0,9705882	0,9705882	0,9640523
orf17	0,9801587	0,1862348	0,984127	0,984127	0,9753087	0,984127	0,984127	0,984127
orf8	0,9771309	0	0,975052	0,9646569	0,972973	0,981289	0,981289	0,985447
orf6	0,9705882	0	0,94	1	0,9826087	0,9913043	0,9913043	0,9558824
jhp_0444	0,9716312	0,9751773	0,7598566	0,9787234	0,9857143	0,9680851	0,9680851	0,9574468
jhp_0369	0,9719298	0,9653979	0,9859649	0,9894737	0,9929824	0,9859649	0,9859649	0,9614035
jhp_0173	0,9739777	0,9814126	0,9702602	0,9776952	0,9739777	0,9739777	0,9739777	0,9814126
jhp_0136	0,9705882	0,9705882	0,9411765	0,9411765	0,9705882	0,9705882	0,9705882	0,9705882
lpp20	0,9885714	0,9942857	0,9942857	0,9942857	0,9942857	0,9942857	0,9942857	0,9885714
jhp_1355	0,9897436	0,9897436	0,9794872	0,9948718	0,9794872	0,9948718	0,994186	0,994186
babB	0,9399707	0,9121813	0,9138418	0,9399707	0,9163059	0,9171348	0,9171348	0,9253294
jhp_1103	0,9157566	0,9262167	0,9203125	0,9063963	0,9359375	0,9015625	0,9015625	0,8907956
babA	0,7360515	0,8536586	0,8362235	0,6998536	0,8967828	0,6952247	0,8927614	0,8443843
jhp_0775	1	0,9950249	1	0,9950249	0,9950249	0,9950249	0,9950249	0,9950249
Cag_14	0,6821316	0,2393617	0,675243	0,9779006	0,6332518	0,9130435	0,9130435	0,6125555
jhp_0119	0,9828326	0,9785408	0,9871244	0,9828326	0,9871244	0,9615384	0,9529914	0,9871244

suppl HP homology of 22 final candidates

Gene name	HP Hp P-4c	HP Hp H-5b	HP Hp P-4d	HP Hp P-8b	HP Hp P-11b	HP Hp P-13b	HP Hp P-25c	HP Hp P-28b
jhp_1276	0,9821429	0,9821429	0,9821429	0,9761904	0,9880952	0,9702381	0,9821429	0,9702381
jhp_0776	1	0,9896373	1	0,9804878	0,9902439	0,9756098	0,9804878	0,9804878
jhp_0718	0,948718	0,972028	0,948718	0,986014	0,9463869	0,983683	0,9463869	0,972028
jhp_0552	0,9811321	0,966457	0,9811321	0,951782	0,9468085	0,951782	0,9601677	0,9468085
jhp_0530	0,9078498	0,9846154	0,9078498	0,9928825	0,9692833	0,9466192	0,9608541	0,9466192
jhp_0501	0,9595016	0,9657321	0,9595016	0,9750779	0,9351852	0,9750779	0,9388379	0,9750779
cagN	0,9738562	0,9673203	0,9738562	0	0,9705882	0,9673203	0,9738562	0
orf17	0,984127	0,9801587	0,984127	0,1862348	0,984127	0,984127	0,9801587	0
orf8	0,975052	0,981289	0,975052	0	0,9646569	0,972973	0,972973	0,981289
orf6	0,94	0,9913043	0,9826087	0	0,9852941	0,9913043	0,96	0,9913043
jhp_0444	0,7598566	0,9751773	0,7598566	0,9751773	0,9787234	0,964539	0,9609929	0,9716312
jhp_0369	0,9859649	0,9789473	0,9859649	0,9653979	0,9894737	0,9789473	0,9929824	0,9894737
jhp_0173	0,9702602	0,9814126	0,9702602	0,9814126	0,9776952	0,9776952	0,9739777	0,9814126
jhp_0136	0,9411765	0,9411765	0,9411765	0,9705882	0,9411765	0,9705882	0,9705882	1
lpp20	0,9942857	0,9942857	0,9942857	0,9942857	0,9942857	0,9942857	0,9942857	0,9942857
jhp_1355	0,9794872	0,974359	0,9794872	0,9897436	0,994186	0,9948718	0,9794872	0,9948718
babB	0,9355783	0,9278499	0,9355783	0,9121813	0,9266573	0,9267936	0,9163059	0,5468531
jhp_1103	0,9203125	0,9141966	0,9203125	0,9262167	0,9063963	0,91875	0,9359375	0,8876755
babA	0,8362235	0,8693333	0,8362235	0,9380165	0,7108604	0,8857527	0,8981233	0,8521257
jhp_0775	1	0,9950249	1	0,9950249	0,9950249	0,9950249	0,9950249	0,9900498
Cag_14	0,6511628	0,9675824	0,6637555	0,2393617	0,6458886	0,6232832	0,6326406	0,2354497
jhp_0119	0,9871244	0,9828326	0,9871244	0,9785408	0,9828326	0,9828326	0,9871244	0,9367089

suppl HP homology of 22 final candidates

Gene name	HP Hp P-25d	HP Hp M2	HP Hp M1	HP Hp M3	HP Hp M4	HP Hp M5	HP Hp M6	HP Hp P-41
jhp_1276	0,9821429	0,975	0,9761904	0,9761904	0,9761904	0,9761904	0,975	0,9821429
jhp_0776	0,9804878	0,9844559	0,9804878	0,9804878	0,9804878	0,9804878	0,9844559	0,9853659
jhp_0718	0,9463869	0,974359	0,974359	0,974359	0,974359	0,974359	0,974359	0,979021
jhp_0552	0,9601677	0,9531915	0,9531915	0,9531915	0,9531915	0,9531915	0,9548387	0,9914894
jhp_0530	0,9608541	0,9893239	0,9893239	0,9861111	0,9893239	0,9893239	0,9861111	0,9466192
jhp_0501	0,9388379	0,9719626	0,9719626	0,9719626	0,9719626	0,9719626	0,9719626	0,9657321
cagN	0,9738562	0,9705882	0,9705882	0,9705882	0,9705882	0,9705882	0,9705882	0,9673203
orf17	0,9801587	0,984127	0,984127	0,984127	0,984127	0,984127	0,984127	0,984127
orf8	0,972973	0,9808511	0,981289	0,981289	0,981289	0,9808511	0,9808511	0,972973
orf6	0,96	0,9913043	0,9913043	0,9913043	0,9913043	0,8016529	0,9913043	0,9652174
jhp_0444	0,9609929	0,9680851	0,9680851	0,9680851	0,9680851	0,964539	0,9680851	0,9157895
jhp_0369	0,9929824	0,9859649	0,9859649	0,9859649	0,9859649	0,9859649	1	0,9824561
jhp_0173	0,9739777	0,9739777	0,9739777	0,9739777	0,9739777	0,9739777	0,9739777	0,9776952
jhp_0136	0,9705882	0,9705882	0,9705882	0,9705882	0,9705882	0,9705882	0,9705882	0,9558824
lpp20	0,9942857	0,9942857	0,9942857	0,9942857	0,9942857	0,9942857	0,9942857	0,9885714
jhp_1355	0,9794872	0,9948718	0,9948718	0,9948718	0,9948718	0,9948718	0,9897436	0,9794872
babB	0,9163059	0,9388646	0,9284579	0,9388646	0,9171348	0,9388646	0,9171348	0,6580406
jhp_1103	0,9359375	0,9015625	0,9015625	0,9015625	0,9015625	0,9015625	0,8972713	0,9235569
babA	0,8679578	0,8927614	0,8914209	0,8927614	0,8803149	0,8927614	0,8927614	0,9168207
jhp_0775	0,9950249	0,9950249	0,9950249	0,9950249	0,9950249	0,9950249	0,9950249	1
Cag_14	0,6326406	0,9563636	0,9353796	0,9285714	0,6225681	1	0,9501134	0,9373368
jhp_0119	0,9871244	0,9529914	0,9615384	0,9615384	0,9656652	0,9529914	0,9529914	0,9785408

suppl HP homology of 22 final candidates

Gene name	HP Hp P-26	HP Hp P-30	HP Hp P-62	HP Hp P-3b	HP Hp P-2b	HP Hp M9	HP R030b	HP R018c
jhp_1276	0,9821429	0,9702381	0,9761904	0,9821429	0,9702381	0,9761904	0,9702381	0,9821429
jhp_0776	0,9756098	0,9658536	0,9756098	1	1	0,9804878	0,9853659	0,9792746
jhp_0718	0,979021	0,9324009	0,9393939	0,969697	0,974359	0,974359	0,941725	0,9277389
jhp_0552	0,951782	0,9853249	0,9853249	0,9580713	0,966457	0,9531915	0,9659575	0,9811321
jhp_0530	0,9555556	0,8668942	0,9217082	0,9679715	0,9044369	0,9893239	0,9857651	0,8657718
jhp_0501	0,9688473	0,8888889	0,9688473	0,9688473	0,9719626	0,9719626	0,9750779	0,8785047
cagN	0,9705882	0	0,9771242	0,9542484	0,9640523	0,9705882	0,9509804	0,9705882
orf17	0,984127	0,1821862	0,9801587	0,9801587	0,9920635	0,984127	0,984127	0,9880952
orf8	0,9771309	0	0,975052	0,9771309	0,9771309	0,981289	0,970894	0,9355509
orf6	0,9913043	0	0,9558824	0,9913043	0,9913043	0,98	0,9826087	0,9652174
jhp_0444	0,9680851	0,9	0,9539007	0,9716312	0,9535714	0,9680851	0,5812274	0,913669
jhp_0369	0,9719298	0,9418182	0,9134948	0,9719298	0,9894737	0,9859649	0,9859649	0,9122807
jhp_0173	0,9739777	0,9132075	0,9739777	0,9739777	0,9814126	0,9739777	0,9407408	0,9033457
jhp_0136	0,9705882	0,9558824	0,9705882	0,9705882	0,9705882	0,9705882	0,9411765	0,9411765
lpp20	0,9942857	0,9885714	0,9828572	0,9885714	0,9942857	0,9942857	0,9942857	0,9942857
jhp_1355	0,9897436	0,9593023	0,9825581	0,9897436	0,9825581	0,9948718	0,9794872	0,974359
babB	0,9264498	0,9220963	0,9447116	0,9448947	0,9067796	0,9388646	0,9036827	0,6505376
jhp_1103	0,9296875	0,7262658	0,925	0,9157566	0,8923557	0,9015625	0,4834532	0,8876755
babA	0,8825911	0,7041096	0,9246299	0,7027819	0,8713137	0,8927614	0,6798867	0,9260753
jhp_0775	0,9950249	0,9850746	0,9950249	1	1	0,9950249	0,9950249	0,9850746
Cag_14	0,9638554	0,2180851	0,9371221	0,6763006	0,6820012	0,9353796	0,6899942	0,5996366
jhp_0119	0,9871244	0,9184549	0,9656652	0,9828326	0,9871244	0,9615384	0	0,9300699

suppl HP homology of 22 final candidates

Gene name	HP R036d	HP R037c	HP R055a	HP R056a	HP Rif1	HP Rif2	HP GAM100Ai
jhp_1276	0,9702381	0,9761904	0,9761904	0,9821429	0,9761904	0,9761904	0,9593023
jhp_0776	0,9707317	0,9756098	0,9804878	0,9792746	0,9658536	0,9658536	1
jhp_0718	0,9300699	0,9230769	0,9207459	0,9277389	0,9230769	0,9230769	0,9705882
jhp_0552	0,9895178	0,9769392	0,9769392	0,9811321	0,9832285	0,9832285	0,9638298
jhp_0530	0,8422819	0,9145908	0,8703071	0,8657718	0,9003559	0,9003559	0,9112628
jhp_0501	0,9183673	0,894081	0,8753894	0,8785047	0,8660436	0,8660436	0,9626168
cagN	0,9411765	0	0,9607843	0,9705882	0,9509804	0,9509804	0,9705882
orf17	0,9880952	0	0,984127	0,9880952	0,9920635	0,9920635	0,9794239
orf8	0,9397089	0	0,9313929	0,9355509	0,9397089	0,9397089	0,9619565
orf6	0,9478261	0	0,9565217	0,9652174	0,9565217	0,9565217	0,973913
jhp_0444	0,9028777	0,9172662	0,8848921	0,913669	0,9172662	0,9172662	0,5698925
jhp_0369	0,9192982	0,899654	0,9017544	0,9122807	0,925	0,925	0,9754386
jhp_0173	0,8801498	0,8764045	0,8951311	0,9033457	0,8764045	0,8764045	0,9814126
jhp_0136	0,9558824	0,9411765	0,9411765	0,9411765	0,9411765	0,9411765	0,9705882
lpp20	0,9885714	0,9942857	0,9885714	0,9942857	1	1	0,9942857
jhp_1355	0,9794872	0,9794872	0,9709302	0,974359	0,9692308	0,9692308	0,9825581
babB	0,9134199	0,5857558	0,6478494	0,6505376	0,8912429	0,8912429	0,6380814
jhp_1103	0,8622848	0,884375	0,8921875	0,8665568	0,8814353	0,8814353	0,9056604
babA	0,8864865	0,6031977	0,9260753	0,9440389	0,9358799	0,9358799	0,5741279
jhp_0775	0,9900498	0,9900498	0,9900498	0,9850746	0,9850746	0,9850746	0,9054054
Cag_14	0,6039183	0,4519231	0,5946724	0,9634783	0,5620135	0,5620135	0,9964285
jhp_0119	0,9265735	0,9335665	0,8285714	0,9291338	0,9405594	0,9405594	0,9894737

suppl HP homology of 22 final candidates

Gene name	HP GAM101Biv	HP GAM114Ai	HP GAM201Ai	HP GAM118Bi	HP GAM210Bi	HP GAM121Aii
jhp_1276	0,9534883	0,9821429	0,9534883	0,9821429	0,9821429	0,9593023
jhp_0776	0,9609756	1	0,9740933	0,9896373	0,9804878	0,9844559
jhp_0718	0,981352	0,9656863	0,9681373	0,9779412	0,9658536	0,9803922
jhp_0552	0,9531915	0,9510638	0,9553192	0,9281314	0,9553192	0,926078
jhp_0530	0,8771331	0,9078498	0,8941979	0,9624574	0,9044369	0,9624574
jhp_0501	0,9376947	0,9688473	0,8785047	0,9595016	0,9657321	0,9657321
cagN	0	0,9738562	0	0,9705882	0,9738562	0,9705882
orf17	0	0,984127	0	0,984127	0,984127	0,984127
orf8	0	0,975052	0	0,6733524	0,975052	0,972973
orf6	0	0,9565217	0	0,9652174	1	0,9913043
jhp_0444	0,9468085	0,7021276	0,9574468	0,5698925	0,9680851	0,9539007
jhp_0369	0,9824561	0,9964913	0,9859649	0,9929824	0,9859649	0,9859649
jhp_0173	0,9219331	0,9813433	0,9851301	0,9776952	0,9962686	0,9776952
jhp_0136	0,9558824	0,9705882	0,9705882	0,9705882	0,9705882	0,9705882
lpp20	0,9828572	0,9885714	0,9942857	0,9942857	0,9942857	0,9942857
jhp_1355	0,9883721	0,9825581	0,9883721	0,9825581	0,9825581	0,9767442
babB	0,9423559	0,9270588	0,9312407	0,9239905	0,9226415	0,9391797
jhp_1103	0,7492114	0,928125	0,7484177	0,9328125	0,9294671	0,9375975
babA	0,8302752	0,8477366	0,7085202	0,8463252	0,8653136	0,6930693
jhp_0775	0,8963964	0,9054054	0,9852217	0,9054054	0,9054054	0,9054054
Cag_14	0,1878307	0,9929578	0,2195767	0,9946524	0,9763889	0,9606742
jhp_0119	0,979021	0,9894737	0,9638989	0,9825175	0,9894737	0,9859649

suppl HP homology of 22 final candidates

Gene name	HP GAM231Ai	HP GAM239Bi	HP GAM249T	HP GAM250AFi	HP GAM252T	HP GAM260ASi
jhp_1276	0,9593023	0,9593023	0,98125	0,98125	0,98125	0,9761904
jhp_0776	0,9609756	0,9560975	1	0,9896373	0,9896373	1
jhp_0718	0,9754902	0,9803922	0,9852941	0,9730392	0,9730392	0,9681373
jhp_0552	0,9531915	0,9702128	0,9510638	0,9553192	0,9553192	0,9489362
jhp_0530	0,8976109	0,9658703	0,8976109	0,9078498	0,9078498	0,9556314
jhp_0501	0,9376947	0,9314642	0,9719626	0,9719626	0,9719626	0,9595016
cagN	0	0	0,9673203	0,9673203	0,9673203	0,9705882
orf17	0	0	0,984127	0,9801587	0,9801587	0,984127
orf8	0	0	0,97921	0,975052	0,975052	0,975052
orf6	0	0	0,9826087	0,9913043	0,9913043	0,9652174
jhp_0444	0,9574468	0,925	0,9574468	0,964539	0,964539	0,9609929
jhp_0369	0,9859649	0,9824561	0,9929824	0,9894737	0,9894737	0,9859649
jhp_0173	0,9814126	0,9665428	0,96	0,9851301	0,9851301	0,9814126
jhp_0136	0,9705882	0,9705882	0,9558824	0,9558824	0,9558824	0,9264706
lpp20	0,9942857	0,9828572	0,9942857	1	1	0,9885714
jhp_1355	0,994186	0,9883721	0,994186	0,9883721	0,9883721	0,9825581
babB	0,8360882	0,9312039	0,8329897	0,9105883	0,9103774	0,937198
jhp_1103	0,692	0,7515823	0,9131147	0,9265625	0,9265625	0,9226891
babA	0,6157025	0,5707899	0,8571429	0,8251029	0,8247423	0,8267223
jhp_0775	0,8963964	0,9009009	0,9054054	0,9571428	0,9571428	0,9054054
Cag_14	0,2195767	0,2163588	0,9964413	0,9966997	0,9811617	0,9964222
jhp_0119	0,979021	0,979021	0,9894737	0,9894737	0,9894737	0,9855596

suppl HP homology of 22 final candidates

Gene name	HP GAM254Ai	HP GAM264Ai	HP GAM270ASi	HP GAM265BSii	HP GAM42Ai	HP GAM83T
jhp_1276	0,975	0,9593023	0,98125	0,9534883	0,9761904	0,9761904
jhp_0776	0,9609756	0,9792746	0,9844559	0,9792746	0,9948186	0,9637306
jhp_0718	0,9852941	0,9730392	0,9557109	0,9828432	0,9393939	0,9730392
jhp_0552	0,9475891	0,9496855	0,9531915	0,9446809	0,9489362	0,9744681
jhp_0530	0,9146758	0,9078498	0,9624574	0,8976109	0,8839591	0,9692833
jhp_0501	0,9657321	0,9314642	0,9719626	0,9719626	0,9719626	0,9252337
cagN	0,9673203	0	0,9673203	0,9705882	0,9705882	0
orf17	0,984127	0	0,984127	0,984127	0,984127	0
orf8	0,985447	0	0,981289	0,975052	0,983368	0
orf6	0,9652174	0	0,973913	0,9913043	0,9826087	0
jhp_0444	0,5985663	0,9550562	0,5698925	0,6129032	0,9574468	0,9214286
jhp_0369	0,9824561	0,9824561	0,9929824	0,9543859	0,9929824	0,9789473
jhp_0173	0,9776952	0,9776952	0,9814126	0,9814126	0,9739777	0,9701493
jhp_0136	0,9705882	0,9558824	0,9411765	0,9705882	0,9705882	0,9705882
lpp20	0,9885714	0,9942857	0,9942857	0,9885714	0,9885714	0,9885714
jhp_1355	0,994186	0,9825581	0,994186	0,9825581	0,994186	0,9883721
babB	0,6293604	0,9495327	0,9178404	0,922049	0,9313132	0,9230769
jhp_1103	0,9359375	0,7444795	0,9342723	0,925	0,9220779	0,7563291
babA	0,8370221	0,5803698	0,8517745	0,8815977	0,8624755	0,8200455
jhp_0775	0,9009009	0,9054054	0,9009009	0,9802955	0,9009009	0,9009009
Cag_14	0,982412	0,2222222	0,9821162	0,9809221	0,9946524	0,2420213
jhp_0119	0,9894737	0,9859649	0,9894737	0,9894737	0,9894737	0,9719298

suppl HP homology of 22 final candidates

Gene name	HP GAM96Ai	HP GAM93Bi	HP HP116Bi	HP HP250ASi	HP HP250ASii	HP HP250BFii	HP HP260AFi
jhp_1276	0,975	0,975	0,9593023	0,98125	0,9821429	0,98125	0,9761904
jhp_0776	0,9689119	0,9896373	0,9740933	0,9896373	0,9896373	0,9896373	1
jhp_0718	0,9705882	0,9779412	0,9656863	0,9730392	0,9730392	0,9730392	0,9681373
jhp_0552	0,966457	0,9702128	0,9531915	0,9553192	0,9531915	0,9553192	0,9489362
jhp_0530	0,8976109	0,9897611	0,8941979	0,9078498	0,9078498	0,9078498	0,9556314
jhp_0501	0,9012346	0,9719626	0,9626168	0,9719626	0,9719626	0,9719626	0,9595016
cagN	0,29683	0,9545454	0,9705882	0,9673203	0,9673203	0,9673203	0,9705882
orf17	0,6388889	0,984127	0,9801587	0,9801587	0,9801587	0,9801587	0,984127
orf8	0,6207585	0,9771309	0,9771309	0,975052	0,975052	0,975052	0,975052
orf6	0,3587786	0,9913043	0,8560606	0,9913043	0,9913043	0,9913043	0,9652174
jhp_0444	0,9142857	0,9577465	0,9539007	0,964539	0,964539	0,964539	0,9609929
jhp_0369	0,9789473	0,9894737	0,9859649	0,9894737	0,9894737	0,9894737	0,9859649
jhp_0173	0,9776952	0,9851301	0,9814126	0,9851301	0,9851301	0,9851301	0,9814126
jhp_0136	0,9705882	0,9705882	0,9705882	0,9558824	0,9558824	0,9558824	0,9264706
lpp20	0,9885714	0,9885714	0,9885714	1	1	1	0,9885714
jhp_1355	0,9883721	0,994186	0,9767442	0,9883721	0,9883721	0,9883721	0,9825581
babB	0,8577878	0,9174107	0,6369748	0,9105883	0,9105883	0,8993711	0,8855932
jhp_1103	0,7436709	0,908922	0,9234375	0,928125	0,928125	0,9265625	0,925
babA	0,8138614	0,8605634	0,9293194	0,8271605	0,8129176	0,8330134	0,8267223
jhp_0775	0,8963964	0,9009009	0,8963964	0,9571428	0,9901478	0,9571428	0,9054054
Cag_14	0,7414722	0,9967267	0,9775596	0,9966997	1	0,9966997	0,9964285
jhp_0119	0,979021	0,9859649	0,9755245	0,9894737	0,9894737	0,9894737	0,9859649

suppl HP homology of 22 final candidates

Gene name	HP HP250BFiV	HP HP260AFii	HP HP260BFii	HP OK113	HP GAMchJs114i	HP GAMchJs136i
jhp_1276	0,9821429	0,9761904	0,9534883	0,9580838	0,9534883	0,9593023
jhp_0776	0,9896373	1	0,9740933	0,9512195	0,9609756	0,9896373
jhp_0718	0,9730392	0,9681373	0,9754902	0,9324009	0,9803922	0,9779412
jhp_0552	0,9553192	0,9489362	0,9702128	0,9460581	0,9553192	0,9281314
jhp_0530	0,9078498	0,9556314	0,9078498	0,9032258	0,8976109	0,9692833
jhp_0501	0,9719626	0,9595016	0,845679	0,8584616	0,9314642	0,9688473
cagN	0,9673203	0,9705882	0	0,9281046	0	0,9705882
orf17	0,9801587	0,984127	0	0,9801587	0	0,984127
orf8	0,975052	0,975052	0	0,9313929	0	0,981289
orf6	0,9913043	0,9652174	0	0,9391304	0	0,9478261
jhp_0444	0,964539	0,9609929	0,863924	0,7142857	0,9539007	0,9539007
jhp_0369	0,9894737	0,9859649	0,9824561	0,9190141	0,9789473	0,9929824
jhp_0173	0,9851301	0,9814126	0,9814126	0,8959108	0,9219331	0,9851301
jhp_0136	0,9558824	0,9264706	0,9558824	0,9264706	0,9705882	0,9705882
lpp20	1	0,9885714	0,9885714	0,9885714	0,9885714	0,9885714
jhp_1355	0,9883721	0,9825581	0,9883721	0,9692308	0,9825581	0,9767442
babB	0,9156626	0,8855932	0,9417293	0,8716502	0,7213115	0,6367583
jhp_1103	0,9226891	0,9228188	0,6897233	0,8767551	0,7488152	0,921875
babA	0,8247423	0,8267223	0,8361905	0,8798921	0,5828313	0,8440861
jhp_0775	0,9901478	0,9054054	0,9802955	0,9900498	0,8918919	0,9054054
Cag_14	0,9968454	0,9964285	0,2248677	0,5924528	0,2248677	0,9856459
jhp_0119	0,9894737	0,9855596	0,968421	0,9106529	0,979021	0,9894737

suppl HP homology of 22 final candidates

Gene name	HP GAMchJs124i	HP CCHI 33	HP Hp H-1	HP UMB_G1	HP UM037	HP PZ5086	HP PZ5026
jhp_1276	0,9761904	0,9761904	0,96875	0,9821429	0,9761904	0,9702381	0,9821429
jhp_0776	0,9689119	0,9853659	0,9707317	0,9658536	0,9707317	0,9705882	0,9658536
jhp_0718	0,9779412	0,967366	0,969697	0,9300699	0,941725	0,9330254	0,9370629
jhp_0552	0,9559748	0,9601677	0,9538784	0,9790356	0,9643606	0,9454927	0,9496855
jhp_0530	0,8976109	0,9078498	0,9692833	0,9180887	0,8225256	0,890785	0,8976109
jhp_0501	0,9345794	0,9688473	0,9439253	0,8878505	0,903125	0,9046154	0,9104939
cagN	0	0,9738562	0,9705882	0	0,9575163	0,9607843	0,9705882
orf17	0	0,984127	0,984127	0	0,9801587	0,984127	0,9880952
orf8	0	0,983368	0,981289	0	0,9501039	0,9272349	0,95842
orf6	0	0,9913043	0,9913043	0	0,9396552	0,9826087	0,9478261
jhp_0444	0,9574468	0,5770609	0,9822695	0,9	0,913669	0,9163498	0,8892857
jhp_0369	0,9824561	0,9754386	0,9824561	0,9307958	0,9192982	0,9192982	0,968421
jhp_0173	0,9628253	0,9739777	0,9851301	0,8913858	0,9107807	0,9222222	0,8913858
jhp_0136	0,9558824	0,9705882	0,9705882	0,9411765	0,9411765	0,9558824	0,9264706
lpp20	0,9885714	0,9942857	0,9942857	0,9942857	0,9942857	1	1
jhp_1355	0,9767442	0,9948718	0,9948718	0,9794872	0,9589744	0,9897436	0,9641026
babB	0,9356322	0,9062049	0,9490085	0,924964	0,897511	0	0
jhp_1103	0,756714	0,9328125	0,9265625	0,7142857	0,8861154	0,8264151	0,8734375
babA	0,5783476	0,8843538	0,9216216	0,7085137	0,8915989	0	0
jhp_0775	0,9852217	0,9950249	0,9950249	0,9900498	0,9900498	0	0,9800995
Cag_14	0,2248677	0,6015958	0,720405	0,2248677	0,8625147	0,9578947	0,9655647
jhp_0119	0,9755245	0,9881423	0,9841897	0,9212598	0,9192982	0,9615384	0,9475524

suppl HP homology of 22 final candidates

Gene name	HP PZ5080	HP PZ5024	HP PZ5004	HP PZ5056	HP GAM117Ai	HP CG-IMSS-2012	HP X47-2AL
jhp_1276	0,9821429	0,9761904	0,9821429	0,9593023	0,98125	0,9761904	0,9761904
jhp_0776	0,9902439	0,9902439	0,9902439	0,9756098	0,9844559	0,9658536	0,9707317
jhp_0718	0,9254079	0,983683	0,974359	0,9393939	0,948718	0,9351852	0,9254079
jhp_0552	0,9853249	0,9350105	0,9433962	0,9371069	0,9553192	0,9853249	0,9832285
jhp_0530	0,828859	0,9180887	0,9044369	0,8839591	0,9078498	0	0,8805461
jhp_0501	0,9190031	0,9750779	0,9750779	0,9688473	0,9719626	0,9158878	0,8847352
cagN	0,9705882	0,9477124	0,9771242	0,9705882	0,9738562	0,9705882	0
orf17	0,984127	0,984127	0,984127	0,9880952	0,984127	0,984127	0
orf8	0,9334719	0,981289	0,975052	0,9521829	0,972973	0	0
orf6	0,9565217	1	1	0,7567568	0,9826087	0,8195488	0
jhp_0444	0,9103943	0,8658147	0,964539	0,9064748	0,964539	0,8848921	0,913669
jhp_0369	0,9508772	0,9377162	0,968421	0,9614035	0,9653979	0,9300699	0,9204152
jhp_0173	0,9296296	0,9702602	0,9702602	0,9259259	0,9814126	0,8768657	0,8764045
jhp_0136	0,9558824	0,9705882	0,9411765	0,9705882	0,9705882	0,9411765	0,9264706
lpp20	0,9885714	0,9942857	0,9942857	0,9942857	0,9942857	1	0,9885714
jhp_1355	0,9641026	0,9948718	0,9948718	0,9794872	0,9767442	0	0,9641026
babB	0,6809524	0	0	0	0,9285714	0,8632911	0,8815233
jhp_1103	0,7142857	0,9078125	0,9301242	0,8677166	0,9189723	0,89375	0,7203792
babA	0,6730159	0	0	0	0,8823529	0,9102564	0,7235543
jhp_0775	1	0,9882353	0	1	0,9054054	0,9800995	0,9850746
Cag_14	0,9658915	0,9752066	0,9773499	0,942647	0,9964602	0,9694794	0,2407407
jhp_0119	0,9719298	0,9824561	0,9824561	0,9543859	0,986014	0,9649123	0,9263158

suppl HP homology of 22 final candidates

Gene name	HP oki102	HP oki112	HP oki422	HP oki898	HP J166	HP NY40	HP 26695-1	HP PMSS1
jhp_1276	0,952381	0,952381	0,952381	0,952381	0,9702381	0,9821429	0,9761904	0,9761904
jhp_0776	0,9756098	0,9658536	0,9756098	0,9756098	0,9658536	0,9844559	0,9658536	0,9707317
jhp_0718	0,934732	0,9300699	0,9324009	0,9324009	0,9393939	0,9607843	0,9230769	0,9351852
jhp_0552	0,9769392	0,9748428	0,9419087	0,9439834	0,9832285	0,9748428	0,9832285	0,9832285
jhp_0530	0,887372	0,887372	0,887372	0,887372	0,8771331	0,8941979	0,8976109	0,8557047
jhp_0501	0,8691589	0	0,8691589	0,8691589	0,8785047	0,9259259	0,8660436	0,8816199
cagN	0,9477124	0,9379085	0,9444444	0,9444444	0,9607843	0,9803922	0,9509804	0,9640523
orf17	0,9801587	0,9801587	0,9801587	0,9880952	0,9880952	0,9801587	0,9920635	0,9960318
orf8	0,9334719	0,9313929	0,9334719	0,9334719	0,9334719	0,9272349	0,9397089	0,9334719
orf6	0,7414966	0,7414966	0,7414966	0,7414966	0,9565217	0,9565217	0,9565217	0,9304348
jhp_0444	0,7617329	0,9208633	0,9208633	0,9208633	0,9097473	0,910072	0,9172662	0,913669
jhp_0369	0,9044369	0,9273356	0,916955	0,916955	0,9190141	0,9333333	0,925	0,9122807
jhp_0173	0,8847584	0,8773234	0,8013698	0,8847584	0,8726591	0,8656716	0,8764045	0,9070632
jhp_0136	0,9411765	0,9411765	0,9411765	0,9411765	0,9558824	0,9411765	0,9411765	0,9411765
lpp20	0,9942857	0,9885714	0,9942857	0,9942857	0,9885714	0,9885714	1	1
jhp_1355	0,9692308	0,9692308	0,9692308	0,9692308	0,9794872	0,9692308	0,9692308	0,9641026
babB	0,6486486	0,6612466	0,8888889	0,8728814	0,9150141	0,9135978	0,8912429	0,9177489
jhp_1103	0,7246835	0,8845554	0,8810642	0,8814353	0,884375	0,8814353	0,8814353	0,8479624
babA	0,8662162	0,8766938	0,8605898	0,8864865	0,9123989	0,7637131	0,9358799	0,7171717
jhp_0775	0,9900498	0,9900498	0,9900498	0,9900498	0,9950249	0,9950249	0,9850746	0,9900498
Cag_14	0,5588998	0,5886487	0,5915931	0,6043406	0,5635703	0,9606189	0,5614945	0,8492308
jhp_0119	0,9370629	0,9300699	0,9265735	0,9265735	0,9440559	0,9685315	0,9405594	0,9475524

suppl HP homology of 22 final candidates

Gene name	HP SS1	HP HPAG1	HP NCTC 11637 = CCUG 17874 = ATCC 43504	HP Hp P-15	HP Hp P-23
jhp_1276	0,9761904	0,9761904	0,9761904	0,9761904	0,9821429
jhp_0776	0,9689119	0,9756098	0,9658536	0,9804878	0,9804878
jhp_0718	0,9351852	0,9254079	0,9254079	0,934732	0,9557109
jhp_0552	0,9832285	0,9853249	0,9769392	0,966457	0,9811321
jhp_0530	0,8976109	0,8839591	0	0,9288256	0,9323843
jhp_0501	0,8816199	0,9034268	0,9593496	0,9657321	0,8878505
cagN	0,9640523	0,9411765	0,9771242	0	0
orf17	0,9960318	0,984127	0,984127	0	0
orf8	0,9397089	0,9313929	0,9313929	0	0
orf6	0,9565217	0,973913	0,973913	0	0
jhp_0444	0,9172662	0,9208633	0,8928571	0,9388489	0,8953069
jhp_0369	0,925	0,9238755	0,925	0,9122807	0,9192982
jhp_0173	0,9070632	0,8847584	0,9033457	0,8801498	0,8801498
jhp_0136	0,9411765	0,9411765	0,9411765	0,9411765	0,9264706
lpp20	1	0,9942857	0,9942857	0,9885714	0,9942857
jhp_1355	0,9692308	0,974359	0,9692308	0,974359	0,9651163
babB	0,9177489	0,9094766	0,5876963	0,8808511	0,9615384
jhp_1103	0,8814353	0,8642746	0,5965583	0,708126	0,7435898
babA	0,7171717	0,9301075	0,8551402	0,729078	0,8341346
jhp_0775	0,9900498	0,9753695	0,9800995	0,9900498	0,9950249
Cag_14	0,9529042	0,9335347	0,9491525	0,3	0,2523364
jhp_0119	0,9475524	0,9265735	0,9265735	0,9263158	0,9263158

suppl HP homology of 22 final candidates

Gene name	HP Hp P-74	HP Hp P-15b	HP UM114	HP oki128	HP Hp P-16	HP 52	HP v225d	HP B8
jhp_1276	0,9702381	0,9761904	0,9761904	0,9356725	0,9702381	0,9520958	0,9461078	0,9642857
jhp_0776	0,9792746	0,9804878	0,9658536	0,9560975	0,9756098	0,9609756	0,9512195	0,9609756
jhp_0718	0,951049	0,934732	0,9189815	0,9300699	0,9440559	0,9324009	0,9277389	0,9254079
jhp_0552	0,9874214	0,9643606	0,9643606	0,9475891	0,9727463	0,9456067	0,932914	0,9811321
jhp_0530	0,8839591	0,9288256	0,9112628	0,9112628	0,9555556	0,9010239	0,9140893	0,8839591
jhp_0501	0,9034268	0,9657321	0,9	0,8193147	0,9003115	0,8878505	0,8765432	0,8785047
cagN	0	0	0,9379085	0	0	0,9477124	0,9346405	0,9512987
orf17	0	0	0,9880952	0	0	0,9801587	0,9880952	0,9880952
orf8	0	0	0,9313929	0	0	0,9355509	0,9313929	0,9334719
orf6	0	0	0,9391304	0	0	0,7619048	0,9565217	0,9565217
jhp_0444	0,9285714	0,9388489	0,9	0,8892857	0,8711111	0,6914893	0,8857143	0,9064748
jhp_0369	0,9157895	0,9122807	0,9236111	0,9236111	0,9157895	0,9236111	0,9260563	0,9190141
jhp_0173	0,8801498	0,8801498	0,8897339	0,8810409	0,8810409	0,8847584	0,8726591	0,8943396
jhp_0136	0,9411765	0,9411765	0,9411765	0,9264706	0,9264706	0,9411765	0,9264706	0,9411765
lpp20	0,9942857	0,9885714	0,9771429	0,9942857	0,9885714	0,9771429	0,9942857	0,9942857
jhp_1355	0,9794872	0,9767442	0,9692308	0,974359	0,9693878	0,9534883	0,9589744	0,9794872
babB	0,9150141	0,7847534	0,910473	0,7739899	0,9291785	0,8787024	0,8852691	0,6601672
jhp_1103	0,7246835	0,721519	0,8904538	0,7101911	0,7192429	0,885759	0,8203125	0,8828125
babA	0,7055631	0,7046071	0,8986486	0,8627187	0,703966	0,8699187	0,8455393	0,8789116
jhp_0775	0,9900498	0,9900498	0,9950249	0,9850746	0,9900498	0,9950249	0,9950249	0,9900498
Cag_14	0,2222222	0,3	0,9110473	0,2195767	0,2248677	0,56902	0,6142534	0,5738605
jhp_0119	0,9368421	0,9263158	0,9125874	0,9100346	0,9300699	0,9134948	0,899654	0,9440559

suppl HP homology of 22 final candidates

Gene name	HP 35A	HP F16	HP F30	HP 83	HP SNT49	HP CPY1124	HP CPY3281	HP CPY6311
jhp_1276	0,9520958	0,9640719	0,9461078	0,9520958	0,9761904	0,9520958	0,9580838	0,9520958
jhp_0776	0,9359606	0,9560975	0,9609756	0,9637306	0,9609756	0,9609756	0,9658536	0,9408867
jhp_0718	0,9393939	0,9324009	0,9324009	0,934732	0,9230769	0,9370629	0,9327146	0,934732
jhp_0552	0,9126016	0,9419087	0,9454927	0,9454927	0,9601677	0,9548387	0,9496855	0,9475891
jhp_0530	0,9072165	0,9139785	0,9103943	0,9692308	0,9097222	0,9139785	0,9072165	0,9106529
jhp_0501	0,8691589	0,8691589	0,8425926	0,8878505	0,909375	0,8753894	0,8660436	0,8785047
cagN	0,9379085	0,9281046	0,9444444	0,9248366	0,9575163	0,9248366	0,9248366	0,9379085
orf17	0,984127	0,984127	0,984127	0,9761904	0,9880952	0,984127	0,9801587	0,9801587
orf8	0,9334719	0,9334719	0,9272349	0,9313929	0,9334719	0,9334719	0,929314	0,9355509
orf6	0,9565217	0,9478261	0,9478261	0,9565217	0,9478261	0,9565217	0,9565217	0,9652174
jhp_0444	0,7285714	0,7127659	0,7071428	0,6950355	0,9107143	0,7	0,7214286	0,7178571
jhp_0369	0,915493	0,9225352	0,9190141	0,915493	0,9306569	0,9260563	0,9447236	0,9119718
jhp_0173	0,8959108	0,8523985	0,8810409	0,8921933	0,8913858	0,8810409	0,8959108	0,8921933
jhp_0136	0,9411765	0,9411765	0,9705882	0,9411765	0,9411765	0,9558824	0,9411765	0,9411765
lpp20	0,9885714	0,9942857	0,9942857	0,9885714	0,9885714	0,9942857	0,9942857	0,9942857
jhp_1355	0,9692308	0,9538462	0,9641026	0,9692308	0,9692308	0,9593023	0,9641026	0,9534883
babB	0,8866856	0,8533145	0,8866856	0,8866856	0,9036827	0,8797737	0,8492958	0,8895184
jhp_1103	0,8765625	0,866562	0,8761755	0,8439938	0,8890625	0,8507937	0,88125	0,8746082
babA	0,8790323	0,8829072	0,8893387	0,6784703	0,9039242	0,8960864	0,8663968	0,864682
jhp_0775	0,8963964	0,9900498	0,9950249	0,9950249	0,9850746	0,9900498	0,9900498	0,9900498
Cag_14	0,6332777	0,626392	0,5815161	0,928344	0,6299388	0,9042857	0,6015303	0,633602
jhp_0119	0,9100346	0,899654	0,916955	0,9134948	0,9020979	0,9029536	0,8987342	0,9156118

suppl HP homology of 22 final candidates

Gene name	HP R32b	HP R046Wa	HP OK310	HP Hp A-11	HP UM032	HP UM299	HP UM066	HP UM298
jhp_1276	0,9642857	0,9702381	0,9520958	0,9583333	0,9640719	0,9640719	0,9520958	0,9640719
jhp_0776	0,9707317	0,9707317	0,9609756	0,9707317	0,9658536	0,9658536	0,9658536	0,9658536
jhp_0718	0,9230769	0,9324009	0,934732	0,9207459	0,9277389	0,9277389	0,934732	0,9277389
jhp_0552	0,9340426	0,9832285	0,9481328	0,9853249	0,9433962	0,9433962	0,9643606	0,9433962
jhp_0530	0,9555556	0,8758389	0,90681	0,9181495	0,8976109	0,8976109	0,9140893	0,8976109
jhp_0501	0,9003115	0,9283489	0,8753894	0,8971962	0,9252337	0,9252337	0,8611111	0,9252337
cagN	0,9477124	0	0,9281046	0,9771242	0,9477124	0,9477124	0,9313725	0,9477124
orf17	0,984127	0	0,9920635	0,9920635	0,9880952	0,9880952	0,9880952	0,9880952
orf8	0,9334719	0	0,9355509	0,9313929	0,9313929	0,9313929	0,929314	0,9313929
orf6	0,9652174	0,9655172	0,9391304	0,92	0,9478261	0,9478261	0,9478261	0,9478261
jhp_0444	0,9280576	0,9028777	0,7214286	0,9208633	0,7198582	0,7198582	0,6950355	0,7198582
jhp_0369	0,9119718	0,9357143	0,9190141	0,9178572	0,915493	0,915493	0,9119718	0,915493
jhp_0173	0,8689138	0,8988764	0,8801498	0,8951311	0,8959108	0,8959108	0,8880597	0,8959108
jhp_0136	0,8970588	0,9264706	0,9558824	0,9264706	0,9558824	0,9558824	0,9411765	0,9558824
lpp20	0,9942857	0,9885714	0,9942857	0,9942857	0,9942857	0,9942857	0,9942857	0,9942857
jhp_1355	0,9589744	0,974359	0,9589744	0,974359	0,9484536	0,9484536	0,9538462	0,9484536
babB	0,6638298	0,6657646	0,7446198	0,9121813	0,8726208	0,8726208	0,8838527	0,8726208
jhp_1103	0,8736349	0,878125	0,8689548	0,8965517	0,8622848	0,8622848	0,8549142	0,8622848
babA	0,6737589	0,9350474	0,8748299	0,8972973	0,8690958	0,8690958	0,8893387	0,8690958
jhp_0775	0,9850746	0,9900498	0,9950249	0,9950249	1	1	1	1
Cag_14	1	0,5111111	0,5677385	0,6084774	0,5818477	0,5818477	0,6271564	0,5818477
jhp_0119	0,9330708	0,9291338	0,9204152	0,9335665	0,9125874	0,9125874	0,8927336	0,9125874

suppl HP homology of 22 final candidates

Gene name	HP UM023	HP UM111	HP UM084	HP UM067	HP UM085	HP FD423	HP FD430	HP FD506
jhp_1276	0,9520958	0,9461078	0,9702381	0,9702381	0,9461078	0,9642857	0,9476744	0,9520958
jhp_0776	0,9408867	0,9463415	0,9609756	0,9658536	0,9609756	0,9560975	0,9609756	0,9707317
jhp_0718	0,9370629	0,9370629	0,9259259	0,9259259	0,941725	0,9212963	0,9259259	0,9300699
jhp_0552	0,9481328	0,9460581	0,9643606	0,9643606	0,9475891	0,9706499	0,966457	0,9481328
jhp_0530	0,9209622	0	0,9146758	0,9078498	0,9140893	0,9078498	0,9146758	0,9175258
jhp_0501	0,8722742	0,878125	0,8965517	0,90625	0,8878505	0,909375	0,896875	0,8875
cagN	0,9346405	0,9346405	0,9313725	0,9575163	0,9379085	0,9444444	0,9509804	0,9248366
orf17	0,9801587	0,984127	0,984127	0,9880952	0,9880952	0,9920635	0,9880952	0,9880952
orf8	0,9313929	0,9355509	0,9355509	0,9290188	0,9331942	0,9290188	0,9313929	0,9269311
orf6	0,9478261	0,9565217	0,9652174	0,9391304	0,9652174	0,973913	0,9478261	0,9565217
jhp_0444	0,7107143	0,8964286	0,9035714	0,9035714	0,9172662	0,9142857	0,9035714	0,9035714
jhp_0369	0,9260563	0,9260563	0,9285714	0,9157895	0,915493	0,9392857	0,9263158	0,9225352
jhp_0173	0,8810409	0,8951311	0,8838952	0,8695652	0,8884758	0,869403	0,8689138	0,8951311
jhp_0136	0,9411765	0,9558824	0,9411765	0,9411765	0,9411765	0,9411765	0,9117647	0,9558824
lpp20	0,9885714	0,9885714	0,9885714	0,9828572	0,9942857	0,9885714	0,9828572	0,9942857
jhp_1355	0,9589744	0,9641026	0,9538462	0,9692308	0,9589744	0,9692308	0,9538462	0,974359
babB	0,8728814	0,8759019	0,886236	0,9335864	0,8929889	0,8918539	0,9214146	0,8617021
jhp_1103	0,8640625	0,6990446	0,8602826	0,9183673	0,8375	0,4578652	0,878125	0,8697017
babA	0,8923284	0,8690958	0,9041835	0,9027027	0,8815612	0,9063772	0,8855172	0,8528428
jhp_0775	0,9950249	0,9950249	0,9900498	1	1	1	0,9863014	1
Cag_14	0,94	0,6332777	0,5641558	0,5802048	0,9076479	0,8571429	0,8400504	0,9128386
jhp_0119	0,9031142	0,9335665	0,9090909	0,9090909	0,9100346	0,8986014	0,9125874	0,9300699

suppl HP homology of 22 final candidates

Gene name	HP FD535	HP FD703	HP FD719	HP GC26	HP BM012A	HP BM012S	HP CPY1313
jhp_1276	0,9476744	0,9642857	0,9702381	0,9580838	0,9702381	0,9702381	0,9520958
jhp_0776	0,9707317	0,9707317	0,9658536	0,9609756	0,9560975	0,9560975	0,9507389
jhp_0718	0,9207459	0,9187935	0,9236111	0,9300699	0,9324009	0,9324009	0,934732
jhp_0552	0,9580713	0,9832285	0,9580713	0,9460581	0,9790356	0,9790356	0,9475891
jhp_0530	0,9146758	0,9044369	0,9146758	0,9180887	0,9112628	0,9112628	0,9103943
jhp_0501	0,9090909	0,90625	0,909375	0,8722742	0,8909658	0,8909658	0,8816199
cagN	0,9248366	0,9542484	0,9444444	0,9248366	0,9411765	0,9411765	0,9444444
orf17	0,9880952	0,9880952	0,9880952	0,984127	0,9920635	0,9920635	0,9880952
orf8	0,9290188	0,9352818	0,9311064	0,9272349	0,929314	0,929314	0,9355509
orf6	0,9565217	0,9652174	0,973913	0,9565217	0,9478261	0,9478261	0,9565217
jhp_0444	0,8956835	0,9071429	0,9064748	0,7142857	0,910072	0,910072	0,7214286
jhp_0369	0,9428571	0,9225352	0,9428571	0,9119718	0,9100346	0,9100346	0,9260563
jhp_0173	0,9056604	0,8913858	0,8951311	0,8843284	0,8981132	0,8981132	0,8847584
jhp_0136	0,9411765	0,9411765	0,9264706	0,9264706	0,9411765	0,9411765	0,9558824
lpp20	0,9942857	0,9885714	0,9828572	0,9942857	0,9885714	0,9885714	0,9942857
jhp_1355	0,974359	0,9538462	0,9692308	0,9589744	0,9641026	0,9641026	0,9593023
babB	0,6572199	0,7632712	0,9192635	0,8833652	0,9134199	0,9150141	0,8578053
jhp_1103	0,7165605	0,8644986	0,8871473	0,847352	0,7235387	0,7235387	0,8640625
babA	0,910162	0,6728838	0,8975741	0,8566879	0,9030955	0,8934579	0,884097
jhp_0775	0,9915966	0,9950249	0,9915254	1	0,9900498	0,9900498	0,9900498
Cag_14	0,6144414	0,6185003	0,9298508	0,9125326	0,5670539	0,5670539	0,7215713
jhp_0119	0,9160839	0,9055944	0,9090909	0,9230769	0,9335665	0,9335665	0,916955

suppl HP homology of 22 final candidates

Gene name	HP SouthAfrica7	HP SouthAfrica20	HP CPY1662	HP Shi470	HP 51	HP Sat464	HP F32
jhp_1276	0,9583333	0,9642857	0,9520958	0,9461078	0,9520958	0,9461078	0,9702381
jhp_0776	0,9707317	0,9792746	0,9560975	0,9512195	0,9408867	0,9609756	0,9458128
jhp_0718	0,9351852	0,8913044	0,9324009	0,934732	0,9300699	0,9393939	0,934732
jhp_0552	0,9460581	0,9685535	0,9622642	0,932914	0,951782	0,9308176	0,9475891
jhp_0530	0	0,8637993	0,9072165	0,9027778	0,9044369	0,8958333	0,90681
jhp_0501	0,8006231	0,84	0,8816199	0,8364198	0,8847352	0,845679	0,8722742
cagN	0	0	0,9313725	0,9313725	0,9248366	0	0,9444444
orf17	0	0	0,984127	0,9801587	0,984127	0,9801587	0,9801587
orf8	0	0,1232877	0,9272349	0,9313929	0,929314	0,8840864	0,929314
orf6	0	0	0,9411765	0,9304348	0,9652174	0,9478261	0,9565217
jhp_0444	0,8521127	0,8556338	0,7214286	0,8964286	0,6879433	0,8964286	0,725
jhp_0369	0,9247312	0,9366197	0,929078	0,9190141	0,9166667	0,9225352	0,915493
jhp_0173	0,8444445	0,862963	0,8876405	0,8884758	0,8921933	0,8884758	0,8614232
jhp_0136	0,9264706	0,9264706	0,9264706	0,9411765	0,9411765	0,9411765	0,9558824
lpp20	0,9828572	0,9885714	0,9942857	0,9942857	0,9885714	0,9885714	0,9885714
jhp_1355	0,9641026	0,948718	0,9593023	0,974359	0,9538462	0,974359	0,9692308
babB	0,7481805	0,822542	0,8852691	0,8707386	0,8787024	0,8828697	0,9134616
jhp_1103	0,7149681	0,7135843	0,875	0,8237129	0,8746082	0,8140625	0,8890625
babA	0,8828571	0,8365123	0,8650472	0,8438762	0,8812416	0,8425303	0,8672087
jhp_0775	0,9850746	0,9900498	0,9950249	1	0,9950249	1	0,9950249
Cag_14	0,5483871	0,1094891	0,6317421	0,5943905	0,6088083	0,63758	0,8016983
jhp_0119	0,8710802	0,8638298	0,9105058	0,9100346	0,9134948	0,9031142	0,9230769

suppl HP homology of 22 final candidates

Gene name	HP F57	HP Shi417	HP Shi169	HP Shi112	HP CPY1962	HP CPY6081	HP CPY6261	HP CPY6271
jhp_1276	0,9520958	0,9461078	0,9461078	0,9461078	0,9580838	0,9580838	0,9583333	0,9580838
jhp_0776	0,9458128	0,9609756	0,9560975	0,9481865	0,9609756	0,9458128	0,9560975	0,9560975
jhp_0718	0,9324009	0,934732	0,9324009	0,9324009	0,9370629	0,4355895	0,9300699	0,9324009
jhp_0552	0,9454927	0,9266247	0,9287212	0,9371069	0,9454927	0,9475891	0,9350105	0,9580713
jhp_0530	0,9103943	0,8986014	0,911032	0,9044369	0,9140893	0,9692308	0,9333333	0,9032258
jhp_0501	0,8722742	0,8395061	0,8364198	0,8549383	0,8722742	0,8785047	0,8909658	0,8753894
cagN	0,9313725	0,9313725	0,9281046	0,9281046	0,9313725	0,9281046	0,9444444	0,9183006
orf17	0,984127	0,9801587	0,9761904	0,9722222	0,984127	0,9880952	0,984127	0,984127
orf8	0,929314	0,9230769	0,9355509	0,9397089	0,929314	0,9313929	0,9209979	0,929314
orf6	0,9478261	0,9304348	0,9304348	0,9304348	0,9318182	0,9565217	0,9478261	0,92
jhp_0444	0,8047619	0,8162544	0,8928571	0,8928571	0,7107143	0,6843972	0,6950355	0,7178571
jhp_0369	0,9260563	0,915493	0,915493	0,915493	0,9225352	0,9190141	0,915493	0,9225352
jhp_0173	0,8884758	0,8830189	0,8773234	0,8830189	0,8996283	0,8921933	0,8921933	0,8847584
jhp_0136	0,9264706	0,9411765	0,9411765	0,9264706	0,9411765	0,9411765	0,9705882	0,9411765
lpp20	0,9885714	0,9942857	0,9942857	0,9942857	0,9942857	0,9885714	0,9885714	0,9942857
jhp_1355	0,9651163	0,9692308	0,9692308	0,9692308	0,9651163	0,9589744	0,9692308	0,9641026
babB	0,8775216	0,8799415	0,6449276	0,6448326	0,8909348	0,8690228	0,8810198	0,8388889
jhp_1103	0,8734375	0,8244514	0,8275862	0,8221529	0,8673947	0,878125	0,8673947	0,8435055
babA	0,8897849	0,8317631	0,8232931	0,8506666	0,9041835	0,8792867	0,8539007	0,8208662
jhp_0775	0,9900498	1	1	0,9900498	0,9900498	0,9900498	0,9900498	0,9800995
Cag_14	0,6037841	0,6349942	0,5890633	0,5906876	0,9126984	0,8732395	0,6141976	0,6311221
jhp_0119	0,9134948	0,899654	0,9065744	0,899654	0,886076	0,8902954	0,8983051	0,9113924

suppl HP homology of 22 final candidates

Gene name	HP A45	HP UM038	HP UM065	HP UM077	HP FD568	HP FD577	HP FD662	HP oki154
jhp_1276	0,9821429	0,9341317	0,9401197	0,9122807	0,9520958	0,9520958	0,9702381	0,9580838
jhp_0776	0,9609756	0,9560975	0,9458128	0,9658536	0,9609756	0,9609756	0,9658536	0,9560975
jhp_0718	0,9254079	0,9300699	0,9324009	0,9300699	0,934732	0,934732	0,9236111	0,9277389
jhp_0552	0,9622642	0,9392034	0,93361	0,939834	0,9643606	0,9460581	0,9601677	0,9454927
jhp_0530	0,9555556	0,9106529	0,9140893	0,9072165	0,9249147	0,9146758	0,9146758	0
jhp_0501	0,8380063	0,8722742	0,8816199	0,875	0,8785047	0,8629283	0,9	0,8255452
cagN	0	0,9183006	0,9379085	0,9346405	0,9313725	0,9346405	0,9411765	0
orf17	0	0,9880952	0,984127	0,9880952	0,984127	0,9880952	0,9880952	0
orf8	0	0,929314	0,9313929	0,929314	0,9334719	0,9251559	0,9290188	0
orf6	0	0,9478261	0,9565217	0,9565217	0,9652174	0,9652174	0,9565217	0
jhp_0444	0,9172662	0,8956835	0,6914893	0,9071429	0,7035714	0,7056738	0,8964286	0,9035714
jhp_0369	0,9122807	0,9119718	0,9084507	0,915493	0,9119718	0,9260563	0,9285714	0,9330986
jhp_0173	0,8876405	0,8921933	0,8847584	0,8996283	0,8847584	0,8880597	0,8913858	0,8847584
jhp_0136	0,9264706	0,9411765	0,9705882	0,9411765	0,9361702	0,9705882	0,9411765	0,9264706
lpp20	0,9828572	0,9942857	0,9885714	0,9885714	0,9828572	0,9885714	0,9828572	0,9942857
jhp_1355	0,974359	0,9538462	0,9692308	0,9641026	0,9641026	0,9641026	0,9538462	0,9692308
babB	0,9072753	0,7266776	0,8871201	0,8694267	0,8966005	0,8740458	0,9065156	0,9004329
jhp_1103	0,7299843	0,8826291	0,8309859	0,8203125	0,8234375	0,8627145	0,8712871	0,7101911
babA	0,7004279	0,8799392	0,6943662	0,8909883	0,8839428	0,8803763	0,8899456	0,8667564
jhp_0775	0,9900498	1	0,9950249	0,9850746	1	0,9863014	0,9950249	0,9900498
Cag_14	0,4888889	0,9291883	0,9106901	0,6021146	0,6087924	0,927242	0,9139942	0,2195767
jhp_0119	0,958042	0,916955	0,899654	0,916955	0,9300699	0,9065744	0,9300699	0,9134948

suppl HP homology of 22 final candidates

Gene name	HP oki673	HP oki828	HP Cuz20	HP Puno120	HP Puno135	HP PeCan18	HP Aklavik117
jhp_1276	0,9580838	0,9580838	0,9580838	0,9821429	0,9401197	0,9821429	0,9640719
jhp_0776	0,9560975	0,9560975	0,9512195	0,9658536	0,9609756	0,9902439	0,9609756
jhp_0718	0,9277389	0,9277389	0,9324009	0,9370629	0,9277389	0,9627039	0,9300699
jhp_0552	0,9496855	0,9475891	0,9308176	0,9350105	0,932914	0,9685535	0,9287212
jhp_0530	0,9106529	0,9106529	0,9072165	0,9078498	0,9106529	0,9658703	0,9027778
jhp_0501	0,8224299	0,8255452	0,8611111	0,8703704	0,8487654	0,9657321	0,8302469
cagN	0	0	0,9281046	0,9339934	0,9240924	0,9379085	0
orf17	0	0	0,9880952	0,9801587	0,9801587	0,984127	0
orf8	0	0	0,9355509	0,929314	0,9376299	0,983368	0
orf6	0	0	0,9304348	0,9478261	0,9391304	0,973913	0,1142857
jhp_0444	0,9035714	0,9035714	0,8892857	0,8510638	0,7833935	0,9071429	0,6308244
jhp_0369	0,915493	0,929078	0,9260563	0,9190141	0,9119718	0,9824561	0,9260563
jhp_0173	0,8847584	0,9029851	0,8943396	0,8773234	0,8773234	0,8838952	0,8401487
jhp_0136	0,9264706	0,8630137	0,9411765	0,9411765	0,9411765	0,9705882	0,9558824
lpp20	0,9942857	0,9942857	0,9942857	0,9942857	0,9942857	0,9942857	0,9942857
jhp_1355	0,9692308	0,9692308	0,9692308	0,9692308	0,9692308	0,9948718	0,9641026
babB	0,6675676	0,6581427	0,822335	0,8828697	0,8711566	0,9224012	0,7403561
jhp_1103	0,7085987	0,7117835	0,830721	0,8141026	0,8127925	0,9233177	0,7245223
babA	0,8679505	0,8696237	0,8211382	0,8364611	0,8485255	0,8598131	0,8623482
jhp_0775	0,9900498	0,9850746	1	0,9950249	1	0,9950249	0,9950249
Cag_14	0,2195767	0,2195767	0,861461	0,5917504	0,5961435	0,612684	0,7012987
jhp_0119	0,916955	0,916955	0,899654	0,9134948	0,9134948	0,9824561	0,9100346

suppl HP homology of 22 final candidates

Gene name	HP NQ4216	HP Hp H-16	HP Hp A-14	HP Hp P-13	HP R038b	HP GAM103Bi	HP GAM105Ai
jhp_1276	0,9821429	0,9821429	0,9761904	0,96875	0,9761904	0,9593023	0,98125
jhp_0776	0,9853659	0,9804878	0,9756098	0,987013	0,9792746	1	1
jhp_0718	0,9463869	0,9440559	0,941725	0,983683	0,9393939	0,9779412	0,9779412
jhp_0552	0,9268817	0,9595745	0,9853249	0,9569892	0,9827957	0,9553192	0,9322382
jhp_0530	0,9125874	0,9537367	0,9060403	0,9846154	0,8805461	0,9010239	0,9846154
jhp_0501	0,9444444	0,9844237	0,8785047	0,9750779	0,8691589	0,9688473	0,9719626
cagN	0,9738562	0,9705882	0	0,9673203	0	0,9738562	0,9738562
orf17	0,984127	0,9801587	0	0,984127	0,1836735	0,984127	0,984127
orf8	0,9334719	0,9723404	0	0,9723404	0	0,983368	0,970894
orf6	0,9558824	0,96	0	0,9772727	0	0,9913043	0,973913
jhp_0444	0,9139785	0,9539007	0,8992805	0,964539	0,9208633	0,964539	0,9539007
jhp_0369	0,922807	0,9192982	0,9263158	0,9789473	0,9074074	0,9964913	0,9789473
jhp_0173	0,9185185	0,9814126	0,8838952	0,9776952	0,9144982	0,9739777	0,9851301
jhp_0136	0,9558824	0,9558824	0,9411765	0,9705882	0,9411765	0,9705882	0,9705882
lpp20	0,9942857	0,9942857	0,9942857	0,9942857	1	0,9942857	0,9942857
jhp_1355	0,9709302	0,9846154	0,974359	0,994186	0,9767442	0,9883721	0,9767442
babB	0,8298969	0,9121523	0,6739131	0,9267936	0,9040903	0,8702461	0,6495845
jhp_1103	0,859375	0,9407176	0,7302839	0,91875	0,7161084	0,9140625	0,9232026
babA	0,8398876	0,8775235	0,7119565	0,8857527	0,7207334	0,8552036	0,9058172
jhp_0775	0,9900498	0,9950249	0,9900498	0,9950249	0,9900498	0,9054054	0,9054054
Cag_14	0,6144414	0,6414662	0,2195767	0,9953271	0,2340426	0,9829457	0,9863014
jhp_0119	0,9824561	0,9894737	0,9230769	0,9828326	0,9370629	0,9894737	0,9894737

suppl HP homology of 22 final candidates

Gene name	HP GAM115Ai	HP GAM112Ai	HP GAM119Bi	HP GAM120Ai	HP GAM244Ai	HP GAM245Ai
jhp_1276	0,9593023	0,9761904	0,975	0,9593023	0,9593023	0,975
jhp_0776	0,9896373	0,9948186	0,9756098	0,9740933	1	1
jhp_0718	0,948718	0,9393939	0,9803922	0,9440559	0,9757282	0,948718
jhp_0552	0,9510638	0,9489362	0,9574468	0,9574468	0,9531915	0,9531915
jhp_0530	0,9078498	0,8839591	0,9112628	0,9846154	0,9112628	0,890785
jhp_0501	0,9657321	0,9719626	0,9719626	0,9750779	0,9439253	0,9626168
cagN	0,9640523	1	0,9640523	0,9640523	0	0,9738562
orf17	0,984127	0,9801587	0,9801587	0,9801587	0	0,9801587
orf8	0,970894	0,983368	0,975052	0,975052	0	0,972973
orf6	0,9826087	0,9826087	0,9652174	1	0	0,9826087
jhp_0444	0,964539	0,9574468	0,9700375	0,9357143	0,9245283	0,5698925
jhp_0369	0,9929824	0,9929824	0,9929824	0,9723184	0,9578947	0,9929824
jhp_0173	0,9776952	0,9739777	0,9850746	0,9776952	0,9813433	0,9851301
jhp_0136	0,9705882	0,9705882	0,9705882	0,9264706	0,9264706	0,9705882
lpp20	1	0,9885714	0,9885714	1	0,9885714	0,9942857
jhp_1355	0,9767442	0,994186	0,9709302	0,9825581	0,9825581	0,994186
babB	0,8932714	0,9405941	0,9322034	0,9017094	0,936	0,9084821
jhp_1103	0,9002079	0,9248827	0,8833593	0,9375975	0,7393365	0,9296875
babA	0,8568075	0,8685613	0,846678	0,8577154	0	0,842572
jhp_0775	0,9054054	0,9009009	0,8963964	0,8963964	0,9009009	0,9054054
Cag_14	0,9964539	0,9949833	0,993311	0,9911972	0,2248677	0,991342
jhp_0119	0,9894737	0,9894737	0,9824561	0,9894737	0,9824561	0,9894737

suppl HP homology of 22 final candidates

Gene name	HP GAM246Ai	HP GAM250T	HP GAM252Bi	HP GAM260Bi	HP GAM260BSi	HP GAM263BFi
jhp_1276	0,9761904	0,9821429	0,9821429	0,9821429	0,9534883	0,98125
jhp_0776	0,9948186	0,9896373	0,9896373	0,9804878	0,9740933	0,9740933
jhp_0718	0,9681373	0,9730392	0,9730392	0,9785523	0,9754902	0,9681373
jhp_0552	0,9425532	0,9553192	0,9553192	0,9643606	0,9702128	0,926078
jhp_0530	0,9624574	0,9078498	0,9078498	0,9624574	0,9078498	0,9624574
jhp_0501	0,9688473	0,9719626	0,9719626	0,9719626	0,845679	0,9688473
cagN	0,9771242	0,9673203	0,9673203	0,9607843	0	0,9705882
orf17	0,984127	0,9801587	0,9801587	0,984127	0	0,9801587
orf8	0,981289	0,975052	0,975052	0,972973	0	0,97921
orf6	0,9826087	0,9913043	0,9913043	0,9826087	0	0,973913
jhp_0444	0,964539	0,964539	0,964539	0,9539007	0,863924	0,9539007
jhp_0369	0,9929824	0,9894737	0,9894737	0,9929824	0,9824561	0,9894737
jhp_0173	0,9814126	0,9851301	0,9851301	0,9851301	0,9814126	0,9739777
jhp_0136	0,9705882	0,9558824	0,9558824	0,9705882	0,9558824	0,9705882
lpp20	0,9885714	1	1	0,9942857	0,9885714	0,9942857
jhp_1355	0,9825581	0,9883721	0,9883721	0,9767442	0,9883721	0,9767442
babB	0,8992974	0,9149485	0,9105883	0,8827434	0,9536232	0,923833
jhp_1103	0,9188768	0,9265625	0,9265625	0,9391575	0,7472354	0,915625
babA	0,82881	0,8106904	0,8251029	0,8407225	0,7	0,8398169
jhp_0775	0,9009009	0,9901478	0,9901478	0,9009009	0,9802955	0,8963964
Cag_14	0,9934211	0,9966997	0,9966997	0,9957447	0,2248677	0,9795159
jhp_0119	1	0,9894737	0,9894737	0,9894737	0,968421	0,9894737

suppl HP homology of 22 final candidates

Gene name	HP GAM268Bii	HP GAM80Ai	HP GAM71Ai	HP GAM83Bi	HP GAMchJs106B	HP HP250AFiii
jhp_1276	0,975	0,98125	0,98125	0,9761904	0,9593023	0,98125
jhp_0776	1	1	0,9637306	0,9637306	0,9896373	0,9896373
jhp_0718	0,9681373	0,9812332	0,9828432	0,9730392	0,9865952	0,9730392
jhp_0552	0,9489362	0,9553192	0,9489362	0,9744681	0,9748428	0,9553192
jhp_0530	0,9846154	0,9078498	0,8941979	0,9692833	0,8976109	0,9078498
jhp_0501	0,9595016	0,9657321	0,9252337	0,9252337	0,9439253	0,9719626
cagN	0,9705882	0,9673203	0	0	0	0,9673203
orf17	0,984127	0,984127	0	0	0	0,9801587
orf8	0,975052	0,97921	0	0	0	0,975052
orf6	0,9652174	0,9652174	0	0	0	0,9913043
jhp_0444	0,9609929	0,6353791	0,9625468	0,9214286	0,9574468	0,964539
jhp_0369	0,9859649	0,9859649	0,9688581	0,9789473	0,9723184	0,9894737
jhp_0173	0,9814126	0,9813433	0,9628253	0,9701493	0,9888476	0,9851301
jhp_0136	0,9264706	0,9411765	0,9705882	0,9705882	0,9705882	0,9558824
lpp20	0,9885714	0,9942857	0,9885714	0,9885714	0,9828572	1
jhp_1355	0,9825581	0,994186	0,9883721	0,9883721	0,9825581	0,9883721
babB	0,8853503	0,9181669	0,9416059	0,9265823	0,6481481	0,9105883
jhp_1103	0,9174455	0,9329173	0,7379095	0,7563291	0,7551343	0,9228188
babA	0,8263598	0,8830645	0,6028985	0,8232759	0,838843	0,8251029
jhp_0775	0,9054054	0,9054054	0,9852217	0,9009009	0,8963964	0,9901478
Cag_14	0,9964222	0,9946524	0,2161458	0,2420213	0,2195767	0,9966997
jhp_0119	0,9855596	0,9859649	0,968421	0,9719298	0,979021	0,9894737

suppl HP homology of 22 final candidates

Gene name	HP HP250AFii	HP HP250AFiV	HP HP250BFiii	HP HP250BFi	HP HP250BSi	HP HP260ASii	HP HP260Bi
jhp_1276	0,9821429	0,9821429	0,9821429	0,9821429	0,98125	0,975	0,98125
jhp_0776	0,9896373	0,9896373	0,9896373	0,9896373	0,9896373	1	0,9804878
jhp_0718	0,9730392	0,9730392	0,9730392	0,9730392	0,9730392	0,9681373	0,9785523
jhp_0552	0,9553192	0,9553192	0,9553192	0,9553192	0,9531915	0,9489362	0,9643606
jhp_0530	0,9078498	0,9078498	0,9078498	0,9078498	0,9078498	0,9556314	0,9624574
jhp_0501	0,9719626	0,9719626	0,9719626	0,9719626	0,9719626	0,9595016	0,9719626
cagN	0,9673203	0,9673203	0,9673203	0,9673203	0,9673203	0,9705882	0,9607843
orf17	0,9801587	0,9801587	0,9801587	0,9801587	0,9801587	0,984127	0,984127
orf8	0,975052	0,975052	0,975052	0,975052	0,975052	0,975052	0,972973
orf6	0,9913043	0,9913043	0,9913043	0,9913043	0,9913043	0,9652174	0,9826087
jhp_0444	0,964539	0,964539	0,964539	0,964539	0,964539	0,9609929	0,9539007
jhp_0369	0,9894737	0,9894737	0,9894737	0,9894737	0,9894737	0,9859649	0,9929824
jhp_0173	0,9851301	0,9851301	0,9851301	0,9851301	0,9851301	0,9814126	0,9851301
jhp_0136	0,9558824	0,9558824	0,9558824	0,9558824	0,9558824	0,9264706	0,9705882
lpp20	1	1	1	1	1	0,9885714	0,9942857
jhp_1355	0,9883721	0,9883721	0,9883721	0,9883721	0,9883721	0,9825581	0,9767442
babB	0,9103774	0,9105883	0,9093024	0,8936651	0,9105883	0,8855932	0,9390787
jhp_1103	0,9263323	0,9265625	0,9265625	0,9265625	0,9244967	0,9228188	0,9391575
babA	0,8247423	0,8251029	0,8349328	0,8251029	0,8230453	0,8267223	0,8298246
jhp_0775	0,9901478	0,9901478	0,9901478	0,9571428	0,9901478	0,9054054	0,9009009
Cag_14	0,9811617	0,9858934	0,9966997	0,9964285	0,9966997	0,9964285	0,9890411
jhp_0119	0,9894737	0,9894737	0,9894737	0,9894737	0,9894737	0,9859649	0,9894737

suppl HP homology of 22 final candidates

Gene name	HP GAMchJs117Ai	HP Hp A-27	HP B128	HP 98-10	HP Aklavik86	HP SouthAfrica50	HP XZ274
jhp_1276	0,9593023	0,9642857	0,9642857	0,9580838	0,9580838	0,9642857	0,9461078
jhp_0776	0,9609756	0,9707317	0,9609756	0,9533679	0,9073171	0,9804878	0,9585492
jhp_0718	0,9828432	0,9230769	0,9254079	0,9324009	0,8530806	0,9088785	0,9236111
jhp_0552	0,9531915	0,9874214	0,9811321	0,9433962	0,9054622	0,9622642	0,9748428
jhp_0530	0,9419795	0,8255033	0,9538462	0,9145908	0,8532423	0,9555556	0
jhp_0501	0,9376947	0,8958333	0,8785047	0,8722742	0,8518519	0,84	0
cagN	0	0,9542484	0,9512987	0,9281046	0	0	0,9477124
orf17	0	0,9801587	0,9880952	0,984127	0	0	0,9801587
orf8	0	0,9313929	0,9334719	0,9313929	0	0	0,929314
orf6	0	0,9431818	0,9565217	0,9565217	0,1061224	0	0,9204546
jhp_0444	0,9574468	0,910072	0,9064748	0,7107143	0,6379929	0,8521127	0,6843972
jhp_0369	0,9859649	0,9285714	0,9190141	0,9225352	0,9263158	0,9726776	0,6593407
jhp_0173	0,9813433	0,9026217	0,8943396	0,8810409	0,8167939	0,9215686	0,8876405
jhp_0136	0,9558824	0,9411765	0,9411765	0,9264706	0,9264706	0,9264706	0,9558824
lpp20	0,9942857	0,9942857	0,9942857	0,9942857	0,875	0,9815951	0,9942857
jhp_1355	0,994186	0,9767442	0,9767442	0,9534883	0,8082902	0,9473684	0
babB	0,8892922	0,890873	0,6343179	0	0,7606061	0,6402684	0,8668555
jhp_1103	0,7539432	0,7156398	0,8571429	0,8734375	0,7791602	0,7093596	0,8642746
babA	0,8508287	0,9179416	0,5710267	0	0,5818182	0,8013423	0,8869448
jhp_0775	0,8963964	0,9850746	0,9900498	0,9900498	0,9701493	0,9850746	0,952381
Cag_14	0,2169312	0,9385066	0,9731286	0,9692898	0,2222222	0,3382353	0,2195767
jhp_0119	0,979021	0,9300699	0,9440559	0,894958	0,7260274	0,8680851	0,9027237

suppl HP homology of 22 final candidates

Gene name	HP NCTC 11639	HP NCTC 11638	HP 17874	Mean	Median	Standard deviation	candidates
jhp_1276	0	0	0	0,9698205	0,9761904	0,01264208	
jhp_0776	0	0	0	0,9751106	0,9756098	0,01516785	
jhp_0718	0	0	0	0,9481373	0,9440559	0,03890266	
jhp_0552	0	0	0	0,9584161	0,9553192	0,01644781	+
jhp_0530	0	0	0	0,9241456	0,9112628	0,03852428	
jhp_0501	0	0	0	0,9232218	0,9314642	0,04684715	
cagN	0	0	0	0,9539809	0,9640523	0,04911292	
orf17	0	0	0	0,9681488	0,984127	0,110493	
orf8	0	0	0	0,9482275	0,9619565	0,06818441	
orf6	0	0	0	0,9484594	0,9652174	0,101003	
jhp_0444	0,2730924	0	0	0,8776104	0,913669	0,113657	
jhp_0369	0	0	0	0,9513015	0,9578947	0,03697531	
jhp_0173	0	0	0	0,9310722	0,9215686	0,04727668	+
jhp_0136	0	0	0	0,9506054	0,9411765	0,01846857	
lpp20	0,9942857	0	0,9885714	0,9921326	0,9942857	0,008482707	
jhp_1355	0	0	0	0,9766062	0,9794872	0,01727135	
babB	0	0	0	0,8585936	0,8992974	0,09947606	
jhp_1103	0	0	0	0,8582215	0,8819562	0,08066645	+
babA	0	0	0	0,8398964	0,8651804	0,08360647	
jhp_0775	0	0	0	0,9763989	0,9900498	0,03385496	+
Cag_14	0	0	0	0,6989317	0,6658522	0,273308	
jhp_0119	0	0	0	0,9505576	0,9597902	0,03750733	+

suppl HP homology of 22 final candidates

Gene name	<33% in bacteria conservation	<5% conservation and -log10 evalue < 1 in human	Protein name
jhp_1276	+	+	
jhp_0776	+	+	
jhp_0718	+	+	
jhp_0552	+	+	
jhp_0530	+	+	
jhp_0501	+	+	
cagN	+	+	
orf17	+	+	
orf8	+	+	
orf6	+	+	
jhp_0444	+	+	
jhp_0369	+	+	
jhp_0173	+	+	
jhp_0136	+	+	
lpp20	+	+	LPP20 lipoprotein
jhp_1355	+	+	
babB	+	+	
jhp_1103	+	+	
babA	+	+	
jhp_0775	+	+	
Cag_14	+	+	
jhp_0119	+	+	

suppl HP homology of 22 final candidates

Gene name	Name	Gene name
jhp_1276	Q9ZJM9	jhp_1276
jhp_0776	Q9ZL06	jhp_0776
jhp_0718	Q9ZL62	jhp_0718
jhp_0552	Q9ZLM7	jhp_0552
jhp_0530	Q9ZLP8	jhp_0530
jhp_0501	Q9ZLS6	jhp_0501
cagN	Q9ZLU0	cagN
orf17	Q9ZLU7	orf17
orf8	Q9ZLV5	orf8
orf6	Q9ZLV7	orf6
jhp_0444	Q9ZLY2	jhp_0444
jhp_0369	Q9ZM54	jhp_0369
jhp_0173	Q9ZMP5	jhp_0173
jhp_0136	Q9ZMT2	jhp_0136
lpp20	P0A0V1	lpp20
jhp_1355	Q9ZJF7	jhp_1355
babB	Q9ZJY3	babB
jhp_1103	Q9ZK39	jhp_1103
babA	Q9ZKV2	babA
jhp_0775	Q9ZL07	jhp_0775
Cag_14	Q9ZLV0	14
jhp_0119	Q9ZMU8	jhp_0119

suppl protein table

Intensity CF_10_1
Intensity CF_10_2
Intensity CF_10_3
Intensity CF_10_4
Intensity CF_20_1
Intensity CF_20_2
Intensity CF_20_3
Intensity CF_20_4
Intensity CF_30_1
Intensity CF_30_2
Intensity CF_30_3
Intensity CF_30_4
Intensity CU_10_1
Intensity CU_10_2
Intensity CU_10_3
Intensity CU_10_4
Intensity CU_20_1
Intensity CU_20_2
Intensity CU_20_3
Intensity CU_20_4
Intensity CU_30_1
Intensity CU_30_2
Intensity CU_30_3
Intensity CU_30_4
Intensity PF_1
Intensity PF_2
Intensity PF_3
Intensity PF_4
Intensity PU_1
Intensity PU_2
Intensity PU_3
Intensity PU_4
Intensity TF_10_1
Intensity TF_10_2
Intensity TF_10_3
Intensity TF_10_4
Intensity TF_20_1
Intensity TF_20_2
Intensity TF_20_3
Intensity TF_20_4
Intensity TF_30_1
Intensity TF_30_2
Intensity TF_30_3
Intensity TF_30_4
Intensity TU_10_1
Intensity TU_10_2
Intensity TU_10_3
Intensity TU_10_4
Intensity TU_20_1
Intensity TU_20_2

Intensity TU_20_3
Intensity TU_20_4
Intensity TU_30_1
Intensity TU_30_2
Intensity TU_30_3
Intensity TU_30_4
iBAQ CF_10_1
iBAQ CF_10_2
iBAQ CF_10_3
iBAQ CF_10_4
iBAQ CF_20_1
iBAQ CF_20_2
iBAQ CF_20_3
iBAQ CF_20_4
iBAQ CF_30_1
iBAQ CF_30_2
iBAQ CF_30_3
iBAQ CF_30_4
iBAQ CU_10_1
iBAQ CU_10_2
iBAQ CU_10_3
iBAQ CU_10_4
iBAQ CU_20_1
iBAQ CU_20_2
iBAQ CU_20_3
iBAQ CU_20_4
iBAQ CU_30_1
iBAQ CU_30_2
iBAQ CU_30_3
iBAQ CU_30_4
iBAQ PF_1
iBAQ PF_2
iBAQ PF_3
iBAQ PF_4
iBAQ PU_1
iBAQ PU_2
iBAQ PU_3
iBAQ PU_4
iBAQ TF_10_1
iBAQ TF_10_2
iBAQ TF_10_3
iBAQ TF_10_4
iBAQ TF_20_1
iBAQ TF_20_2
iBAQ TF_20_3
iBAQ TF_20_4
iBAQ TF_30_1
iBAQ TF_30_2
iBAQ TF_30_3
iBAQ TF_30_4

iBAQ TU_10_1
iBAQ TU_10_2
iBAQ TU_10_3
iBAQ TU_10_4
iBAQ TU_20_1
iBAQ TU_20_2
iBAQ TU_20_3
iBAQ TU_20_4
iBAQ TU_30_1
iBAQ TU_30_2
iBAQ TU_30_3
iBAQ TU_30_4
LFQ intensity CF_10_1
LFQ intensity CF_10_2
LFQ intensity CF_10_3
LFQ intensity CF_10_4
LFQ intensity CF_20_1
LFQ intensity CF_20_2
LFQ intensity CF_20_3
LFQ intensity CF_20_4
LFQ intensity CF_30_1
LFQ intensity CF_30_2
LFQ intensity CF_30_3
LFQ intensity CF_30_4
LFQ intensity CU_10_1
LFQ intensity CU_10_2
LFQ intensity CU_10_3
LFQ intensity CU_10_4
LFQ intensity CU_20_1
LFQ intensity CU_20_2
LFQ intensity CU_20_3
LFQ intensity CU_20_4
LFQ intensity CU_30_1
LFQ intensity CU_30_2
LFQ intensity CU_30_3
LFQ intensity CU_30_4
LFQ intensity PF_1
LFQ intensity PF_2
LFQ intensity PF_3
LFQ intensity PF_4
LFQ intensity PU_1
LFQ intensity PU_2
LFQ intensity PU_3
LFQ intensity PU_4
LFQ intensity TF_10_1
LFQ intensity TF_10_2
LFQ intensity TF_10_3
LFQ intensity TF_10_4
LFQ intensity TF_20_1
LFQ intensity TF_20_2

LFQ intensity TF_20_3
LFQ intensity TF_20_4
LFQ intensity TF_30_1
LFQ intensity TF_30_2
LFQ intensity TF_30_3
LFQ intensity TF_30_4
LFQ intensity TU_10_1
LFQ intensity TU_10_2
LFQ intensity TU_10_3
LFQ intensity TU_10_4
LFQ intensity TU_20_1
LFQ intensity TU_20_2
LFQ intensity TU_20_3
LFQ intensity TU_20_4
LFQ intensity TU_30_1
LFQ intensity TU_30_2
LFQ intensity TU_30_3
LFQ intensity TU_30_4
GOBP slim name
GOCC slim name
GOMF name
KEGG name
Keywords
Peptides
Unique peptides
Sequence coverage [%]
Mol. weight [kDa]
Majority protein IDs
Gene name

suppl table biotinylation

Column	Type	Name	Size	Score	P value	Benj. Hoch	Mean	Median
Welch's T-t	Welch's T-t	LFQ intensi	565	0,985431	8,83E-76	8,83E-76	1,414705	1,349841
Welch's T-t	GOMF nam	transferase	18	-0,46878	0,000677	0,41383	0,659402	0,710377
Welch's T-t	Keywords	S-adenosyl-	10	-0,60598	0,00099	0,211823	0,53908	0,5945
Welch's T-t	GOMF nam	oxidoreduc	82	0,222842	0,001018	0,311104	1,428967	1,393399
Welch's T-t	GOMF nam	methyltran	12	-0,54929	0,001093	0,222524	0,561365	0,534377
Welch's T-t	GOMF nam	antioxidant	8	0,659801	0,001319	0,201426	1,9914	1,967279
Welch's T-t	GOBP slim	macromole	19	-0,42037	0,001755	0,256185	0,713293	0,651388
Welch's T-t	Keywords	Methyltran	9	-0,6055	0,001783	0,190815	0,494034	0,373154
Welch's T-t	GOMF nam	oxidoreduc	5	0,771429	0,002929	0,357878	2,24421	2,320288
Welch's T-t	Keywords	Chaperone	16	0,429418	0,003288	0,234568	1,664257	1,667485
Welch's T-t	GOBP slim	protein fol	10	0,520513	0,004671	0,340983	1,812826	1,848557
Welch's T-t	GOMF nam	cis-trans is	4	0,771893	0,00771	0,785112	2,041758	2,090672
Welch's T-t	GOMF nam	peptidyl-pr	4	0,771893	0,00771	0,672953	2,041758	2,090672
Welch's T-t	Keywords	Rotamase	4	0,771893	0,00771	0,412473	2,041758	2,090672
Welch's T-t	Keywords	Membrane	67	-0,19588	0,008258	0,353429	0,993224	0,996539
Welch's T-t	GOBP slim	glycerol et	3	0,879643	0,008501	0,413691	2,253869	2,207802
Welch's T-t	GOMF nam	protein me	3	-0,87776	0,008642	0,660055	0,116343	0,248043
Welch's T-t	GOBP slim	RNA proces	12	-0,41929	0,012679	0,462765	0,754836	0,57505
Welch's T-t	Keywords	Electrontra	12	0,411667	0,01439	0,513241	1,616263	1,569683
Welch's T-t	Keywords	Cellmembr	53	-0,20079	0,014929	0,456392	0,975768	0,996539
Welch's T-t	KEGG nam	Flagellar as	13	-0,39232	0,015272	0,862885	0,819492	0,848347
Welch's T-t	Keywords	Redox-activ	5	0,626591	0,015669	0,419135	1,894857	1,796419
Welch's T-t	Keywords	Lipidbiosyn	12	0,404762	0,016114	0,383148	1,616197	1,633733
Welch's T-t	Keywords	Lipidmetab	12	0,404762	0,016114	0,344833	1,616197	1,633733
Welch's T-t	GOMF nam	tetrapyrrol	9	0,464201	0,016617	0,922972	1,665639	1,518157
Welch's T-t	GOBP slim	response to	4	0,692797	0,01678	0,489982	2,178491	2,229213
Welch's T-t	GOMF nam	oxidoreduc	6	0,5661	0,016851	0,858002	1,855561	1,682776
Welch's T-t	GOMF nam	peroxidase	6	0,5661	0,016851	0,792002	1,855561	1,682776
Welch's T-t	Keywords	Peroxidase	6	0,5661	0,016851	0,327831	1,855561	1,682776
Welch's T-t	Keywords	Transmeml	45	-0,21206	0,017167	0,306141	0,971239	1,064005
Welch's T-t	GOCC slim	plasma me	53	-0,19002	0,021245	0,552373	0,986039	1,072012
Welch's T-t	GOBP slim	DNA modif	6	-0,54533	0,021322	0,518832	0,535785	0,320809
Welch's T-t	KEGG nam	Toluene de	3	0,764927	0,022118	0,833111	2,003444	1,976561
Welch's T-t	GOBP slim	generation	15	-0,33429	0,026611	0,555025	0,797638	0,746731
Welch's T-t	Keywords	Oxidoreduc	49	0,186198	0,029486	0,48538	1,409156	1,365668
Welch's T-t	Keywords	Disulfidebo	10	0,398291	0,030416	0,464926	1,594849	1,496785
Welch's T-t	GOBP slim	cell cycle	6	-0,49528	0,036533	0,666721	0,688579	0,4894
Welch's T-t	GOBP slim	cell divisior	6	-0,49528	0,036533	0,592641	0,688579	0,4894
Welch's T-t	Keywords	Nucleotide	89	-0,13644	0,037165	0,530226	1,058212	1,054768
Welch's T-t	Keywords	Tricarboxyl	7	0,452077	0,039396	0,526927	1,79078	1,924252
Welch's T-t	GOBP slim	cellular hor	4	0,59322	0,040584	0,592523	2,064362	2,276902
Welch's T-t	KEGG nam	Butanoate	9	0,381697	0,048907	0,921084	1,6325	1,924594
Welch's T-t	GOCC slim	membrane	78	-0,13646	0,04903	0,637384	1,048056	1,085968
Welch's T-t	Keywords	Stressrespc	7	0,429787	0,050177	0,63164	1,77024	2,209612
Welch's T-t	Keywords	Diaminopir	6	0,462229	0,051007	0,606419	1,567547	1,58817
Welch's T-t	KEGG nam	Folate bios	3	-0,63893	0,055953	0,903244	0,505492	0,370437
Welch's T-t	Keywords	Cellcycle	9	-0,37	0,056255	0,633609	0,807742	0,502505
Welch's T-t	Keywords	Celldivision	9	-0,37	0,056255	0,601929	0,807742	0,502505
Welch's T-t	Keywords	rRNAproce	3	-0,63705	0,05668	0,577591	0,52867	0,695599

Welch's T-t	GOBP slim	cell motility	8	-0,39063	0,05723	0,759602	0,818823	0,836146
Welch's T-t	GOBP slim	cellular cor	8	-0,39063	0,05723	0,696302	0,818823	0,836146
Welch's T-t	KEGG name	Selenocom	4	0,541667	0,061512	0,868856	1,801476	1,841867
Welch's T-t	Keywords	Cellinnerm	37	-0,18198	0,06211	0,604164	0,983229	1,075962
Welch's T-t	Keywords	Tryptophar	6	-0,43909	0,063775	0,593386	0,710405	0,664713
Welch's T-t	Keywords	Fattyacidbi	4	0,534605	0,064977	0,579374	1,765741	1,891406
Welch's T-t	Keywords	Fattyacidm	4	0,534605	0,064977	0,556199	1,765741	1,891406
Welch's T-t	GOBP slim	RNA modifi	7	-0,40142	0,067372	0,756644	0,76875	0,591565
Welch's T-t	KEGG name	Carbon fixa	19	0,2425	0,071078	0,89243	1,501785	1,42201
Welch's T-t	GOBP slim	RNA metab	45	-0,15995	0,07225	0,753465	1,019329	1,116182
Welch's T-t	GOBP slim	heterocycle	74	-0,12751	0,072311	0,703823	1,065894	1,046828
Welch's T-t	KEGG name	Nitrogen m	9	0,34819	0,072412	0,818258	1,512824	1,469255
Welch's T-t	Keywords	Zinc	23	-0,21928	0,073358	0,603796	0,956083	0,872905
Welch's T-t	GOBP slim	cellular am	5	-0,46421	0,073403	0,669803	0,702353	0,651388
Welch's T-t	Keywords	Nuclease	3	-0,59097	0,077071	0,610859	0,612331	0,718507
Welch's T-t	GOBP slim	nucleobase	116	-0,10339	0,077835	0,668463	1,086144	1,169352
Welch's T-t	KEGG name	Two-compo	17	0,248921	0,079248	0,814089	1,452434	1,504244
Welch's T-t	KEGG name	Citrate cycl	15	0,264275	0,079635	0,749893	1,54408	1,709472
Welch's T-t	Keywords	Bacterialfla	6	-0,41076	0,082891	0,633525	0,790623	0,682093
Welch's T-t	KEGG name	Valine, leuc	4	0,497175	0,086123	0,748604	1,74872	1,749902
Welch's T-t	KEGG name	Tryptophar	3	0,569346	0,088523	0,714506	1,840074	2,138139
Welch's T-t	KEGG name	RNA degrac	5	-0,44102	0,08897	0,670243	0,738996	0,488208
Welch's T-t	KEGG name	Lipopolysac	10	-0,3114	0,090574	0,639678	0,819777	0,789574
Welch's T-t	GOBP slim	prosthetic g	3	-0,55806	0,095021	0,770726	0,646343	0,527048
Welch's T-t	Keywords	Glycolysis	8	-0,34055	0,097357	0,718428	0,801317	0,760038
Welch's T-t	Keywords	Transmemb	37	-0,16084	0,099191	0,707562	1,017734	1,085848
Welch's T-t	GOBP slim	pathogenes	5	-0,42348	0,102424	0,787048	0,79959	0,851653
Welch's T-t	GOCC slim	small ribos	5	-0,42008	0,105206	0,911787	0,69797	0,699175
Welch's T-t	GOBP slim	tRNA proce	9	-0,31089	0,1087	0,793512	0,867812	0,591565
Welch's T-t	GOBP slim	tRNA meta	28	-0,17794	0,110181	0,76602	0,991034	1,190688
Welch's T-t	GOBP slim	nitrogen cc	192	-0,0776	0,111706	0,741322	1,116353	1,191479
Welch's T-t	Keywords	GTP-bindin	11	-0,27869	0,112413	0,776015	0,899004	0,685563
Welch's T-t	GOBP slim	cellular nitr	175	-0,07971	0,11295	0,716984	1,112712	1,178702
Welch's T-t	GOBP slim	DNA replica	9	0,30678	0,113453	0,690175	1,453221	1,480083
Welch's T-t	GOBP slim	one-carbor	5	-0,4082	0,115418	0,674042	0,752035	0,70089
Welch's T-t	GOBP slim	nucleoside	19	-0,21045	0,11725	0,658402	0,963989	1,052112
Welch's T-t	GOCC slim	cell part	295	-0,06823	0,120609	0,783958	1,135948	1,175433
Welch's T-t	KEGG name	Cysteine ar	11	0,272468	0,120667	0,802078	1,418006	1,545048
Welch's T-t	GOBP slim	oligosaccha	3	-0,51857	0,12082	0,653321	0,47784	0,229017
Welch's T-t	Keywords	Ubiquinone	4	-0,44421	0,125182	0,837156	0,709759	0,658222
Welch's T-t	Keywords	Pyrimidinel	9	-0,29635	0,126255	0,818745	0,875867	1,126378
Welch's T-t	Keywords	Queuosinel	4	-0,44209	0,126993	0,79931	0,722562	0,743685
Welch's T-t	Keywords	Elongation	4	0,439266	0,12944	0,791431	1,861357	2,042499
Welch's T-t	Keywords	ATP-bindin	77	-0,10523	0,131228	0,780077	1,083629	1,159154
Welch's T-t	KEGG name	Pentose ph	11	0,263909	0,13278	0,833565	1,457156	1,698979
Welch's T-t	Keywords	Lysinebiosy	7	0,32847	0,134453	0,777647	1,449618	1,587493
Welch's T-t	Keywords	Kinase	14	-0,23148	0,137734	0,775659	0,954222	1,003872
Welch's T-t	KEGG name	Arginine ar	15	0,223529	0,138191	0,821873	1,425896	1,405078
Welch's T-t	Keywords	Aromaticar	9	-0,28718	0,13841	0,759478	0,875182	0,818616
Welch's T-t	KEGG name	Epithelial c	3	-0,48942	0,143155	0,808828	0,611547	0,24476

Welch's T-t	Keywords	Heme	8	0,294389	0,151836	0,812324	1,472192	1,441913
Welch's T-t	KEGG name	Peptidogly	4	-0,41243	0,154539	0,831569	0,776098	0,554189
Welch's T-t	GOBP slim	macromole	138	-0,07708	0,159348	0,830884	1,114875	1,190688
Welch's T-t	Keywords	2Fe-2S	3	0,464034	0,165079	0,861631	1,685093	1,965696
Welch's T-t	KEGG name	Taurine and	4	0,398305	0,169155	0,868844	1,660999	1,710122
Welch's T-t	Keywords	Decarboxyl	8	-0,28125	0,170964	0,8711	0,886157	0,885658
Welch's T-t	GOBP slim	macromole	5	0,351627	0,175067	0,881371	1,566546	1,611809
Welch's T-t	GOBP slim	protein cor	5	0,351627	0,175067	0,851992	1,566546	1,611809
Welch's T-t	Keywords	Transferase	91	-0,08694	0,180053	0,89608	1,091243	1,154905
Welch's T-t	KEGG name	Phenylalan	3	0,437706	0,190389	0,935391	1,647007	1,621209
Welch's T-t	Keywords	Metallopro	3	-0,43488	0,193262	0,939956	0,770855	0,647106
Welch's T-t	GOBP slim	multi-orgar	7	-0,2843	0,195155	0,919119	0,939147	0,851653
Welch's T-t	Keywords	Pyruvate	6	-0,30642	0,195791	0,931093	0,919141	0,729725
Welch's T-t	GOBP slim	homeostat	8	0,26456	0,197792	0,902428	1,60767	1,521809
Welch's T-t	GOBP slim	polysaccha	9	-0,24925	0,198435	0,877925	0,93535	0,710336
Welch's T-t	GOBP slim	response to	22	0,158893	0,204133	0,876571	1,395769	1,243411
Welch's T-t	Keywords	Repeat	17	0,17901	0,206896	0,962518	1,325611	1,406724
Welch's T-t	KEGG name	Fatty acid b	8	0,258168	0,208844	0,983308	1,293165	1,570829
Welch's T-t	Keywords	Virulence	4	-0,36229	0,211082	0,961098	0,861597	0,953211
Welch's T-t	Keywords	4Fe-4S	12	-0,20881	0,214464	0,956153	0,982888	0,672885
Welch's T-t	GOBP slim	protein mo	6	-0,29367	0,215042	0,897034	0,826102	1,090996
Welch's T-t	GOBP slim	cellular aro	29	-0,13435	0,220028	0,892335	1,054992	0,994518
Welch's T-t	Keywords	Nucleotidyl	12	-0,20619	0,220269	0,961992	0,947854	0,959814
Welch's T-t	Keywords	Aminotrans	6	0,289896	0,221	0,945881	1,461458	1,503983
Welch's T-t	Keywords	LipidAbiosy	3	0,408557	0,221622	0,929942	1,561078	1,471703
Welch's T-t	GOBP slim	biosyntheti	209	-0,05746	0,226833	0,895069	1,12526	1,181076
Welch's T-t	Keywords	DNAreplica	7	0,263627	0,229637	0,945044	1,421937	1,437346
Welch's T-t	GOBP slim	nucleotide-	6	-0,28376	0,230933	0,887267	0,918956	0,904346
Welch's T-t	GOBP slim	cell wall or	6	-0,26912	0,255886	0,957932	0,915327	0,739526
Welch's T-t	GOBP slim	cellular cell	6	-0,26912	0,255886	0,933984	0,915327	0,739526
Welch's T-t	GOBP slim	cellular pro	347	-0,0491	0,257	0,915172	1,156755	1,200639
Welch's T-t	GOBP slim	secretion	3	-0,37189	0,265914	0,924367	0,788757	0,469011
Welch's T-t	GOMF nam	zinc-export	1	0	1	1	1,423049	1,423049
Welch's T-t	GOBP slim	translation	1	0	1	1	1,497211	1,497211
Welch's T-t	GOCC slim	tricarboxyli	1	0	1	1	2,169277	2,169277
Welch's T-t	KEGG name	Vibrio chol	2	0,330986	1	1	1,546974	1,546974
Welch's T-t	Keywords	Zymogen	1	0	1	1	0,631787	0,631787

suppl table proteins showing expected cluster for surface association (Fig2)

Protein IDs	Majority pr	Gene name
P64102	P64102	jhp_1339
Q9ZJ38	Q9ZJ38	jhp_1479
Q9ZJ66	Q9ZJ66	secD
Q9ZJ91	Q9ZJ91	hsdM_3
Q9ZJC9	Q9ZJC9	jhp_1383
Q9ZJD1	Q9ZJD1	jhp_1381
Q9ZJE0	Q9ZJE0	jhp_1372
Q9ZJL3	Q9ZJL3	lon
Q9ZJL8	Q9ZJL8	clpX
Q9ZJM9	Q9ZJM9	jhp_1276
Q9ZJP5	Q9ZJP5	exbD
Q9ZJQ2	Q9ZJQ2	dnaJ
Q9ZJT5	Q9ZJT5	rpoA
Q9ZJV3	Q9ZJV3	pflA
Q9ZJY9	Q9ZJY9	carA
Q9ZK82	Q9ZK82	atpC
Q9ZKB7	Q9ZKB7	jhp_1022
Q9ZKM2	Q9ZKM2	ftsZ
Q9ZKM3	Q9ZKM3	ftsA
Q9ZKQ1	Q9ZKQ1	accD
Q9ZKQ3	Q9ZKQ3	jhp_0882
Q9ZKY5	Q9ZKY5	pgbB
Q9ZL06	Q9ZL06	jhp_0776
Q9ZL29	Q9ZL29	motB
Q9ZL35	Q9ZL35	fliL
Q9ZL62	Q9ZL62	jhp_0718
Q9ZL83	Q9ZL83	rny
Q9ZLD9	Q9ZLD9	gyrA
Q9ZLM7	Q9ZLM7	jhp_0552
Q9ZLP8	Q9ZLP8	jhp_0530
Q9ZLP9	Q9ZLP9	tonB_1
Q9ZLQ8	Q9ZLQ8	jhp_0520
Q9ZLS6	Q9ZLS6	jhp_0501
Q9ZLT1	Q9ZLT1	cagA
Q9ZLU0	Q9ZLU0	cagN
Q9ZLU7	Q9ZLU7	orf17
Q9ZLV3	Q9ZLV3	virD4
Q9ZLV5	Q9ZLV5	orf8
Q9ZLV7	Q9ZLV7	orf6
Q9ZLY2	Q9ZLY2	jhp_0444
Q9ZM23	Q9ZM23	dnaJ_1
Q9ZM24	Q9ZM24	jhp_0399
Q9ZM54	Q9ZM54	jhp_0369
Q9ZM66	Q9ZM66	ftsH
Q9ZM93	Q9ZM93	lepA
Q9ZMA2	Q9ZMA2	jhp_0320
Q9ZMI7	Q9ZMI7	jhp_0233
Q9ZML8	Q9ZML8	jhp_0200
Q9ZMP5	Q9ZMP5	jhp_0173

Q9ZMP9	Q9ZMP9	jhp_0169
Q9ZMT2	Q9ZMT2	jhp_0136
Q9ZMT3	Q9ZMT3	fixP
Q9ZMY7	Q9ZMY7	jhp_0075
Q9ZN48	Q9ZN48	jhp_0011
P0A0V1	P0A0V1	lpp20
Q9ZJA0	Q9ZJA0	jhp_1413
Q9ZJF6	Q9ZJF6	jhp_1356
Q9ZJF7	Q9ZJF7	jhp_1355
Q9ZJG2	Q9ZJG2	jhp_1350
Q9ZJY3	Q9ZJY3	babB
Q9ZK39	Q9ZK39	jhp_1103
Q9ZK87	Q9ZK87	jhp_1054
Q9ZKG1	Q9ZKG1	jhp_0975
Q9ZKV2	Q9ZKV2	babA
Q9ZL07	Q9ZL07	jhp_0775
Q9ZLC5	Q9ZLC5	dnaX
Q9ZLJ9	Q9ZLJ9	jhp_0580
Q9ZLN5	Q9ZLN5	jhp_0543
Q9ZLV0	Q9ZLV0	14
Q9ZLW8	Q9ZLW8	pgbA
Q9ZM26	Q9ZM26	fur
Q9ZMU8	Q9ZMU8	jhp_0119
Q9ZMX0	Q9ZMX0	jhp_0095

Welch's T-tes	Welch's T-tes	Type	Size	P value	Benj. Hoch. F	Names
#!{Type}E	E	C	N	N	N	T
#!{C:Group1}	LFQ intensity negBU					
0.9660678	-0.1575424	Welch's T-tes	590	0	0	+
-0.08416786	0.8374318	Welch's T-tes	170	0	0	+
0.9660678	-0.1575424	Welch's T-tes	590	0	0	LFQ intensity posBU_LFQ intensity negBU
-0.08416786	0.8374318	Welch's T-tes	170	0	0	LFQ intensity TF_10_LFQ intensity CF_10
0.0245417	0.3727679	GOCC slim na	94	1,00E+09	0.00030131	membrane
0.01561018	-0.2013623	GOMF name	516	8,00E+09	0.05807625	catalytic activity
-0.1197737	-0.1566667	GOBP slim na	270	0.00014242	0.02235995	cellular nitrogen compound metabolic process
0.3298151	-0.1147477	GOMF name	92	0.00015604	0.05664420	oxidoreductase activity
-0.00839250	0.3521655	Keywords	79	0.00016451	0.03734517	Membrane
-0.05085107	0.4387659	Keywords	50	0.00017771	0.02017120	Ribonucleoprotein
-0.06742676	0.4356533	GOCC slim na	51	0.00017819	0.00267287	ribonucleoprotein complex
0.0297379	0.3825779	GOCC slim na	62	0.00018224	0.00182241	plasma membrane
-0.2863262	0.3332616	GOMF name	60	0.00018519	0.04481682	nucleoside-triphosphatase activity
-0.4485081	-0.0750212	GOBP slim na	41	0.00020156	0.01582312	macromolecule modification
-0.1053493	-0.154885	GOBP slim na	291	0.00023118	0.01209892	nitrogen compound metabolic process
-0.0133808	0.3777673	Keywords	64	0.00025365	0.01919340	Cellmembrane
0.02203882	0.1834454	GOCC slim na	387	0.00025643	0.00192326	cell part
-0.04485813	0.4190996	GOMF name	52	0.00026373	0.04786788	structural molecule activity
-0.03853912	0.4286148	GOMF name	49	0.00027920	0.04054038	structural constituent of ribosome
-0.03853912	0.4286148	KEGG name	49	0.00027920	0.03322525	Ribosome
-0.03853912	0.4286148	Keywords	49	0.00027920	0.01584481	Ribosomalprotein
-0.1691597	0.2249149	GOMF name	148	0.00033505	0.04054127	nucleic acid binding
0.07388116	-0.2216374	GOBP slim na	214	0.00034381	0.01349470	small molecule metabolic process
-0.0937693	0.3180181	GOCC slim na	86	0.00047153	0.00282918	macromolecular complex
0.03299925	0.4697382	Keywords	35	0.00048170	0.02186962	rRNA-binding
0.01426741	0.4634871	GOMF name	36	0.00054512	0.05653735	rRNA binding
-0.4775	-0.1009722	GOBP slim na	30	0.00055626	0.01746676	RNA processing

0.00150364	0.3697373	GOBP slim na	54	0.00088458[illegible]	0.02314664[illegible]	translation
-0.5311795	-0.06273478	GOBP slim na	22	0.00138142[illegible]	0.03098334[illegible]	RNA modification
-0.2184632	0.2908342	GOMF name	64	0.00142011[illegible]	0.12887572[illegible]	pyrophosphatase activity
-0.00317124[illegible]	0.3862195	GOCC slim na	44	0.00168113[illegible]	0.00840569[illegible]	intracellular non-membrane-bounded organelle
-0.00317124[illegible]	0.3862195	GOCC slim na	44	0.00168113[illegible]	0.00720487[illegible]	intracellular organelle
-0.00317124[illegible]	0.3862195	GOCC slim na	44	0.00168113[illegible]	0.00630426[illegible]	organelle
-0.5098031	0.05177202	Keywords	25	0.00177481[illegible]	0.06714723[illegible]	S-adenosyl-L-methionine
-0.2163026	0.2757773	GOMF name	66	0.00184830[illegible]	0.14909677[illegible]	hydrolase activity, acting on acid anhydrides
-0.2163026	0.2757773	GOMF name	66	0.00184830[illegible]	0.13418709[illegible]	hydrolase activity, acting on acid anhydrides,
0.2396336	-0.5689739	Keywords	19	0.00189888[illegible]	0.06157825[illegible]	Pyridoxalphosphate
-0.08336412	0.3463102	Keywords	55	0.00208642[illegible]	0.05920224[illegible]	RNA-binding
-0.1970909	0.113888	GOMF name	165	0.00218968[illegible]	0.14451942[illegible]	purine ribonucleoside triphosphate binding
-0.1970909	0.113888	GOMF name	165	0.00218968[illegible]	0.13247613[illegible]	purine ribonucleotide binding
-0.1970909	0.113888	GOMF name	165	0.00218968[illegible]	0.12228566[illegible]	ribonucleotide binding
-0.1983677	0.1144772	Keywords	151	0.00295576[illegible]	0.07455097[illegible]	Nucleotide-binding
-0.1677739	0.1055434	GOBP slim na	221	0.00301379[illegible]	0.05914581[illegible]	macromolecule metabolic process
-0.4743058	0.08003511	GOMF name	26	0.00318738[illegible]	0.16528884[illegible]	methyltransferase activity
-0.1891011	0.1098666	GOMF name	166	0.00320044[illegible]	0.15490163[illegible]	purine nucleotide binding
-0.1626604	-0.06980032	GOBP slim na	189	0.00323861[illegible]	0.05649590[illegible]	nucleobase-containing compound metabolic process
-0.3353489	0.1525011	GOBP slim na	47	0.00417759[illegible]	0.06558820[illegible]	DNA metabolic process
-0.4932382	0.0798272	Keywords	22	0.00471348[illegible]	0.10699603[illegible]	Methyltransferase
-0.3969207	-0.02022443	GOMF name	32	0.00493068[illegible]	0.22372978[illegible]	transferase activity, transferring one-carbon groups
-0.2556977	0.1963151	GOMF name	63	0.00505816[illegible]	0.21601322[illegible]	DNA binding
-0.06120854	-0.2089121	GOBP slim na	104	0.00558530[illegible]	0.07971758[illegible]	heterocycle metabolic process
0.01859179	0.5066399	GOBP slim na	19	0.00569508[illegible]	0.07451074[illegible]	protein transport
-0.1021729	-0.1367073	GOMF name	159	0.00697397[illegible]	0.28128365[illegible]	transferase activity
0.1327317	-0.243421	GOBP slim na	78	0.00703306[illegible]	0.08493776[illegible]	cellular amino acid metabolic process
-0.1874635	0.091427	GOMF name	146	0.00711860[illegible]	0.27200555[illegible]	adenyl ribonucleotide binding
-0.1874635	0.091427	GOMF name	146	0.00711860[illegible]	0.25840527[illegible]	ATP binding
0.001382132	0.354268	GOCC slim na	38	0.00720197[illegible]	0.02400658[illegible]	ribosome

0.7367617	-0.3612525	GOMF name	8	0.007531379	0.260370565	antioxidant activity
-0.2671507	0.2920791	GOMF name	37	0.007781182	0.256779007	ATPase activity
0.06350872	0.2223005	GOBP slim na	83	0.007903297	0.088629840	protein metabolic process
-0.2270665	-0.06823516	GOBP slim na	77	0.008181818	0.085636364	RNA metabolic process
-0.01592005	0.3244915	Keywords	45	0.008201479	0.169248706	Cellinnermembrane
-0.03988557	-0.1204057	GOBP slim na	469	0.008255066	0.081002837	metabolic process
0.01592831	0.3180377	Keywords	44	0.008959440	0.169482751	Transmembranehelix
-0.1025948	-0.1443448	Keywords	135	0.008990352	0.156985383	Transferase
-0.5458462	0.1197265	GOMF name	15	0.009645653	0.304467140	S-adenosylmethionine-dependent methyltransferase activity
-0.5716873	0.2380128	GOMF name	13	0.009658833	0.292179721	endonuclease activity
-0.178638	0.08707967	GOMF name	147	0.010158548	0.295004240	adenyl nucleotide binding
-0.4028926	0.2707551	GOMF name	22	0.010483479	0.292731000	nuclease activity
0.1199892	-0.1965133	GOBP slim na	105	0.011009080	0.101672093	organic acid metabolic process
-0.1831603	0.09214523	Keywords	132	0.011398105	0.184812144	ATP-binding
0.08581772	-0.2189017	GOBP slim na	93	0.011774020	0.102695624	amine metabolic process
0.06510154	-0.3193684	GOMF name	43	0.011834898	0.318227258	lyase activity
-0.02822437	0.2761701	GOBP slim na	57	0.012478341	0.103110504	establishment of localization
-0.02822437	0.2761701	GOBP slim na	57	0.012478341	0.097954979	transport
0.4663932	-0.3314188	KEGG name	15	0.012644243	0.752332470	Arginine and proline metabolism
0.4915555	-0.2720684	KEGG name	15	0.013219162	0.524360130	Citrate cycle (TCA cycle)
-0.06302368	0.2416175	GOMF name	75	0.013243260	0.343378821	RNA binding
0.2719956	-0.04939734	Keywords	57	0.013978367	0.211539299	Oxidoreductase
-0.5230755	0.3945585	Keywords	11	0.014501622	0.205741768	Nuclease
0.03365446	-0.2455978	GOBP slim na	68	0.016176324	0.120937285	nucleobase-containing small molecule metabolic process
-0.6183674	0.1418367	GOBP slim na	10	0.016430198	0.117251871	rRNA processing
-0.3791724	-0.1832419	GOBP slim na	18	0.016956707	0.115747962	tRNA processing
0.7989848	-0.4919797	GOMF name	5	0.016969360	0.424819160	oxidoreductase activity, acting on a sulfur group of donors
-0.4325615	-0.08457772	GOBP slim na	17	0.019012659	0.124374480	DNA repair
-0.4325615	-0.08457772	GOBP slim na	17	0.019012659	0.119399501	response to DNA damage stimulus
-0.05680381	-0.09482919	GOBP slim na	433	0.019178053	0.115805937	cellular metabolic process

0.0792181	0.4207819	Keywords	18	0.019627793	0.262088768	Proteintransport
-0.4745935	-0.50271	Keywords	6	0.020603839	0.259837310	Queuosinebiosynthesis
-0.001496638	0.2620141	Keywords	52	0.021437881	0.256126269	Transmembrane
0.3679381	0.1245361	Keywords	20	0.022373814	0.253942797	Chaperone
-0.6242362	0.3039715	Keywords	8	0.023905840	0.258410755	Zinc-finger
0.722561	-0.3252032	KEGG name	6	0.024791863	0.737557941	Glutathione metabolism
0.01954757	-0.294346	Keywords	39	0.025701658	0.265194383	Lyase
0.399729	0.5335366	Keywords	6	0.026145653	0.258046233	Lipoprotein
0.6044852	-0.1170008	GOMF name	9	0.026179659	0.633547764	heme binding
0.6044852	-0.1170008	GOMF name	9	0.026179659	0.613110739	tetrapyrrole binding
0.4973624	-0.04621683	Keywords	13	0.027365161	0.258828814	Electrontransport
0.3869478	-0.129687	KEGG name	21	0.029116379	0.692969823	Carbon fixation pathways in prokaryotes
0.8047667	-0.5263692	GOBP slim na	4	0.029132515	0.169400184	response to oxidative stress
0.2251624	-0.436718	GOMF name	15	0.030158711	0.684225774	pyridoxal phosphate binding
0.2251624	-0.436718	GOMF name	15	0.030158711	0.663491660	vitamin B6 binding
0.08020661	-0.1717562	GOBP slim na	110	0.030500493	0.171020622	cellular ketone metabolic process
-0.2316051	-0.1210332	Keywords	39	0.030604727	0.277890929	Zinc
0.2987954	-0.4397292	KEGG name	13	0.030977981	0.614396631	Pentose phosphate pathway
-0.1483403	-0.2807214	GOBP slim na	26	0.031016689	0.167917937	nucleoside metabolic process
-0.6378433	0.3062055	Keywords	7	0.031152968	0.271989382	Endonuclease
0.507551	-0.3204082	KEGG name	10	0.031562278	0.536558729	Nitrogen metabolism
0.1009483	0.193293	Keywords	56	0.032452064	0.272837724	Transport
0.81643	-0.4295132	GOMF name	4	0.034339013	0.733238928	cis-trans isomerase activity
0.81643	-0.4295132	GOMF name	4	0.034339013	0.712289244	peptidyl-prolyl cis-trans isomerase activity
0.81643	-0.4295132	Keywords	4	0.034339013	0.278391285	Rotamase
-0.3137625	0.6177881	GOCC slim na	7	0.035875456	0.107626369	small ribosomal subunit
-0.1269727	0.07194263	GOMF name	196	0.036248748	0.731016421	nucleotide binding
0.6842818	-0.2560976	GOMF name	6	0.036552558	0.717220471	oxidoreductase activity, acting on peroxide as acceptor
0.6842818	-0.2560976	GOMF name	6	0.036552558	0.698346248	peroxidase activity
0.6842818	-0.2560976	Keywords	6	0.036552558	0.286118302	Peroxidase

-0.5179592	0.2414286	GOMF name	10	0.037495782	0.697998416	helicase activity
-0.5179592	0.2414286	Keywords	10	0.037495782	0.283718089	Helicase
0.1927298	-0.3831733	GOMF name	18	0.038256100	0.694348228	vitamin binding
0.3296165	0.06488018	GOBP slim na	23	0.038325678	0.200571050	response to chemical stimulus
-0.07394013	-0.2345791	GOBP slim na	42	0.039493113	0.200013510	cellular aromatic compound metabolic process
0.5442293	-0.2313965	KEGG name	9	0.039963568	0.594458083	Butanoate metabolism
-0.4299898	0.4732688	GOMF name	8	0.042877074	0.759237956	N-methyltransferase activity
-0.5222449	0.09918367	Keywords	10	0.043110637	0.315681123	rRNAprocessing
0.4291498	0.9787449	GOMF name	2	0.043222023	0.747123543	iron ion transmembrane transporter activity
0.1074879	0.2949448	Keywords	24	0.043634741	0.309533950	Repeat
0.620404	-0.1187327	Keywords	7	0.044079988	0.303216893	Tricarboxylicacidcycle
-0.5322233	-0.003058104	GOMF name	9	0.045805166	0.773361648	RNA methyltransferase activity
0.3519316	0.1600684	Keywords	15	0.045872554	0.306266762	Signal
-0.2729469	-0.1365597	GOMF name	24	0.046364905	0.765020940	zinc ion binding
0.5894309	-0.4109079	Keywords	6	0.046833366	0.303747833	Diaminopimelatebiosynthesis
0.28	0.6004061	Keywords	5	0.047314886	0.298346644	Palmitate
